# Safety and Comparability of Controlled Human *Plasmodium falciparum* Infection by Mosquito Bite in Malaria-Naïve Subjects at a New Facility for Sporozoite Challenge

**DOI:** 10.1371/journal.pone.0109654

**Published:** 2014-11-18

**Authors:** Angela K. Talley, Sara A. Healy, Olivia C. Finney, Sean C. Murphy, James Kublin, Carola J. Salas, Susan Lundebjerg, Peter Gilbert, Wesley C. Van Voorhis, John Whisler, Ruobing Wang, Chris F. Ockenhouse, D. Gray Heppner, Stefan H. Kappe, Patrick E. Duffy

**Affiliations:** 1 Malaria Clinical Trials Center, Seattle Biomedical Research Institute, Seattle, Washington, United States of America; 2 Laboratory for Malaria Immunology and Vaccinology, National Institute of Allergy and Infectious Diseases, National Institutes of Health, Bethesda, Maryland, United States of America; 3 Department of Laboratory Medicine, University of Washington Medical Center, Seattle, Washington, United States of America; 4 Fred Hutchinson Cancer Research Center, Seattle, Washington, United States of America; 5 United States Naval Medical Research Unit Number 6, Lima, Peru; 6 Department of Medicine, University of Washington Medical Center, Seattle, Washington, United States of America; 7 United States Military Malaria Vaccine Program, Walter Reed Army Institute of Research, Silver Spring, Maryland, United States of America; Mahidol-Oxford Tropical Medicine Research Unit, Thailand

## Abstract

**Background:**

Controlled human malaria infection (CHMI) studies which recapitulate mosquito-borne infection are a critical tool to identify protective vaccine and drug candidates for advancement to field trials. In partnership with the Walter Reed Army Institute of Research, the CHMI model was established at the Seattle Biomedical Research Institute's Malaria Clinical Trials Center (MCTC). Activities and reagents at both centers were aligned to ensure comparability and continued safety of the model. To demonstrate successful implementation, CHMI was performed in six healthy malaria-naïve volunteers.

**Methods:**

All volunteers received NF54 strain *Plasmodium falciparum* by the bite of five infected *Anopheles stephensi* mosquitoes under controlled conditions and were monitored for signs and symptoms of malaria and for parasitemia by peripheral blood smear. Subjects were treated upon diagnosis with chloroquine by directly observed therapy. Immunological (T cell and antibody) and molecular diagnostic (real-time quantitative reverse transcriptase polymerase chain reaction [qRT-PCR]) assessments were also performed.

**Results:**

All six volunteers developed patent parasitemia and clinical malaria. No serious adverse events occurred during the study period or for six months post-infection. The mean prepatent period was 11.2 days (range 9–14 days), and geometric mean parasitemia upon diagnosis was 10.8 parasites/µL (range 2–69) by microscopy. qRT-PCR detected parasites an average of 3.7 days (range 2–4 days) earlier than blood smears. All volunteers developed antibodies to the blood-stage antigen merozoite surface protein 1 (MSP-1), which persisted up to six months. Humoral and cellular responses to pre-erythrocytic antigens circumsporozoite protein (CSP) and liver-stage antigen 1 (LSA-1) were limited.

**Conclusion:**

The CHMI model was safe, well tolerated and characterized by consistent prepatent periods, pre-symptomatic diagnosis in 3/6 subjects and adverse event profiles as reported at established centers. The MCTC can now evaluate candidates in the increasingly diverse vaccine and drug pipeline using the CHMI model.

**Trial Registration:**

ClinicalTrials.gov NCT01058226

## Introduction

In the absence of defined immune correlates of protection and consistently predictive animal models, controlled human malaria infections (CHMI) have become the most effective means of assessing early-stage efficacy of candidate pre-erythrocytic and erythrocytic vaccines and anti-malarial drugs. Under this model, malaria parasite-infected mosquitoes are allowed to bite human volunteers to inoculate them with *Plasmodium* sporozoites under controlled conditions. To date, CHMI has mostly been performed using *Plasmodium falciparum*-infected mosquitoes [1]–[8], although a few *Plasmodium vivax* studies have recently taken place as well [9], [10]. In all trials, the subjects are monitored closely for development of patent blood-stage parasitemia and treated with standard doses of anti-malarial medications defined by the known sensitivity profile of the parasite. The parasite densities reported from CHMI studies can be used for modeling parasite growth kinetics [11], [12].

CHMI studies have been conducted in the United States of America (USA) and elsewhere for decades. Reviews of the published literature indicate the model is safe, reproducible and well-tolerated by subjects participating in clinical challenge trials with *P. falciparum* [6], [7], [13]. Over the last 20 years, over 900 subjects have been experimentally infected or “challenged” with malaria at the Walter Reed Army Institute of Research (WRAIR), with an outstanding safety record and no occurrence of complicated malaria or Serious Adverse Events (SAEs). Importantly, the CHMI model plays a critical role in early phase testing of candidate interventions in order to prioritize only the most promising candidates for further product development efforts.

Despite an expanding pipeline of candidate malaria interventions in development, the number of clinical research centers capable of conducting CHMI is limited [14]. To expand the global capacity for CHMI studies, Seattle Biomedical Research Institute (Seattle BioMed) established the Malaria Clinical Trials Center (MCTC) as an innovative center for the integration of basic science and clinical research with an immediate mandate to support the development of an effective malaria vaccine. This study (ClinicalTrials.gov NCT01058226) was performed to demonstrate the successful implementation of the CHMI model at Seattle BioMed, prior to conducting efficacy trials of candidate malaria vaccines.

## Methods

### Objectives

The primary objective of the study was to demonstrate the safety, tolerability and infectivity of CHMI in a newly-established facility. The secondary objectives were to assess the malaria-specific immune response following CHMI in healthy malaria-naïve adults. Additionally, we sought to evaluate the detection and quantification of prepatent parasitemia by real-time quantitative reverse transcriptase polymerase chain reaction (qRT-PCR).

### Study Site

The study was conducted at the MCTC at Seattle BioMed in Seattle, WA. The institute also houses the Center for Mosquito Production and Malaria Infection Research (CeMPMIR), an entomology facility that was upgraded for the production of malaria sporozoites and malaria-infected mosquitoes under phase-appropriate current Good Manufacturing Practices (cGMPs) to support MCTC trials. The Clinical and Translational Research Laboratory (CTRL) was simultaneously established to support specimen processing and to perform standardized assays according to Good Clinical Laboratory Practices (GCLPs) for clinical trials.

Seattle BioMed and WRAIR established a Cooperative Research and Development Agreement to institute the CHMI model at the MCTC. WRAIR research and clinical staff from the US Military Malaria Vaccine Program served in an advisory capacity throughout preparation and conduct of the trial to ensure alignment with WRAIR practices and participant safety.

### Study design

**Figure 1:**
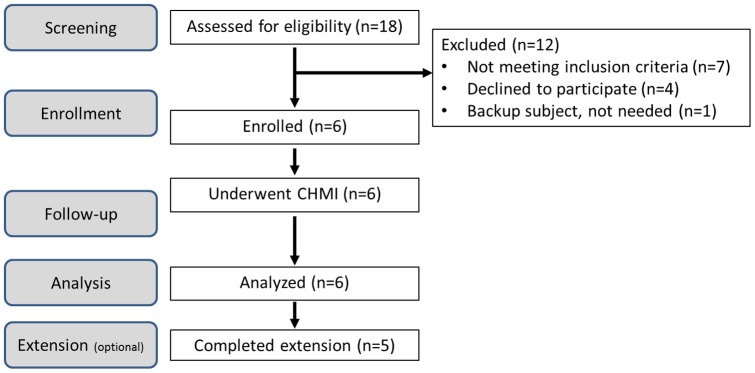
Study flow diagram. Eighteen subjects were screened for eligibility to participate in the trial and 7 healthy volunteers were considered eligible and willing to participate. On the day of enrollment, 6 subjects were enrolled and one backup subject was discharged from the study. The six subjects underwent CHMI and completed the 56 day study. Five subjects returned for optional long term safety and immunology follow up assessments at 3 and 6 months post-challenge.

This was a prospective, open-label, single intervention study of six healthy malaria-naïve adult volunteers undergoing CHMI. All subjects were enrolled as a single cohort and followed the same study schedule (**Figure 1**). On the day of enrollment (Day 0), six eligible subjects underwent CHMI (‘challenge’) according to standard procedures [5]. Briefly, subjects were infected with *P. falciparum* sporozoites by the bites of five infected *Anopheles stephensi* mosquitoes under controlled conditions. Volunteers were closely monitored in the post-challenge period and treated with standard oral doses of chloroquine phosphate upon diagnosis of malaria parasitemia by positive thick blood films. Periodic clinical assessments, physical examinations and laboratory monitoring were performed for evaluation of protocol-defined safety, infectivity and immunology endpoints.

### Ethical conduct

This study was conducted in accordance with the International Conference of Harmonization (ICH) Good Clinical Practices (GCP) and applicable Food and Drug Administration (FDA) regulations. Based on evolving regulatory standards for human challenge studies, the P. falciparum sporozoite was considered an investigational product and for the first time, the CHMI procedures and study protocol were conducted under an investigational new drug application (IND) filed with the FDA. Prior to study initiation, the protocol and study-related documents were approved by the Western Institutional Review Board. The study was registered on ClinicalTrials.gov (Identifier NCT01058226; see http://clinicaltrials.gov/show/NCT01058226). The protocol for this trial and supporting CONSORT checklist are available as supporting information; see **Checklist S1** and **Protocol S1**.

### Study population

Subjects were recruited using IRB-approved messaging from the greater Seattle area. Subjects provided written acknowledgment of informed consent and were required to pass an assessment of understanding quiz covering key study concepts and risks related to participation. Adult males and non-pregnant females aged 18–50 years were eligible to participate in the study if they were in good general health as demonstrated by detailed medical history review, physical examination and laboratory assessment performed within 56 days of enrollment. Subjects were required to have a low risk of coronary heart disease based on the NHANES 1 screening criteria [15] and a normal or essentially normal electrocardiogram as read by an independent cardiologist. Laboratory assessment of eligibility included measurements of hemoglobin (Hb), white blood cells (WBC), platelets, serum creatinine, alanine aminotransferase (ALT), aspartate aminotransferase (AST), total bilirubin and alkaline phosphatase, along with urine dipstick or full urinalysis, and screening for active infection with HIV-1/-2, hepatitis B and hepatitis C viruses. Subjects with a prior diagnosis of malaria or recent travel to a malaria-endemic area were excluded, as were those who had previously received an investigational malaria vaccine, recent malaria chemoprophylaxis or antibiotics with anti-malarial properties. Inclusion and exclusion criteria are available in the IRB-approved protocol (**Protocol S1**).

### Investigational product (*P. falciparum* sporozoites)

Wild-type NF54 strain *P. falciparum*-infected *A. stephensi* mosquitoes for use in CHMI were reared in compliance with phase-appropriate cGMPs in the secure CeMPMIR entomology facility according to standard procedures adapted from WRAIR. The *A. stephensi* mosquitoes were a laboratory-reared strain originally received from WRAIR. Mosquitoes were infected with *P. falciparum* by standard membrane feeding methods on parasite cultures derived from the WRAIR master cell bank (MCB) containing a sufficient proportion of mature gametocytes. Prior to the CHMI, sensitivity testing of the parasite MCB to the anti-malarial drugs chloroquine, doxycycline, atovaquone and quinine was performed. The results confirmed the sensitivity of the NF-54 parasite MCB to these drugs at published values and were virtually identical to the sensitive type strain, *P. falciparum* 3D7, a clone of NF-54. On the day of challenge, five infected mosquitoes were placed in a screen-covered carton and allowed to feed on the subject's forearm for five minutes. Following the feed, mosquitoes were evaluated to confirm both an adequate blood meal (as evidenced by the presence of a blood meal in the abdomen) and the presence of adequate numbers of sporozoites in their salivary glands (by individual mosquito dissections). The sporozoite load was rated microscopically according to a semi-quantitative scale: 0 (no sporozoites observed), +1 (1–10), +2 (11–100), +3 (101–1000) and +4 (>1000) [5]. Only mosquitoes with a salivary gland rating of +2 or greater were considered infective. Feeding iterations were repeated using additional mosquitoes as needed to ensure each subject received a total of five infective bites.

### Post-challenge monitoring and follow up

Subjects were closely monitored for 30–45 minutes post-challenge for acute reactogenicity and were issued a symptom diary and thermometer to record symptoms and oral temperatures daily for five days. Subjects were evaluated in clinic on Day 1 post-challenge, contacted by phone daily on Days 2-4 and evaluated again in clinic daily on Days 5–8 post-challenge. From Day 9 post-challenge, subjects were housed in a local hotel with study staff for close observation during the time they were expected to develop patent parasitemia and symptoms of clinical malaria infection. Clinical assessments conducted at each visit included symptom review, vital signs and physical examination. Peripheral blood smears were examined for detection of malaria parasites at least daily beginning on Day 5 post-infection and continuing through diagnosis and treatment. Blood samples for safety endpoint assays were collected prior to challenge, on the day of the first positive blood smear and on Days 35 and 56 post-challenge. Blood samples for immunology endpoint assays were collected at baseline, on the day of the first positive blood smear, on Days 1, 5, 35 and 56 post-challenge.

Malaria treatment decisions were based on diagnosis of patent (microscopic) parasitemia by a positive peripheral blood smear. Subjects were treated upon detection of patent parasitemia with a standard oral regimen of oral chloroquine phosphate (600 mg chloroquine base initially, followed by 300 mg chloroquine base at 6, 24 and 48 hours later) under direct observation. Subjects continued their overnight hotel stays until completion of treatment and confirmation of three consecutive daily negative blood smears, after which they were followed weekly for eight weeks. Subjects were invited to return for optional long-term immunology assessments at three and six months post-infection.

Primary safety endpoints were assessed by clinical and laboratory evaluations following the challenge and clinically significant changes from baseline status were reported as adverse events (AEs). Solicited AEs were captured on a diary card and/or by direct questioning during the 28 days post-challenge. A solicited AE was defined as a predetermined event that may reflect safety concerns related to the investigational product or events that could be reasonably expected to occur as part of the intervention. These included local and systemic signs and symptoms related to the challenge and/or clinical malaria. (**Table 1**). While not previously established to be related to mild malaria infection, for the purpose of ensuring safety in this initial challenge study, cardiac AEs were solicited by symptom review including chest pain. Local and systemic reactogenicity related to the challenge was collected from the day of challenge (Day 0) through Day 5 post-challenge. Systemic symptoms related to clinical malaria were collected from Day 5 through Day 28 post-challenge. All other AEs reported at any time during the study (Day 0 to Day 56 post-challenge) were recorded as unsolicited AEs, including any worsening or exacerbation of pre-existing conditions. All subjects were contacted by phone six months post-malaria challenge to assess for the occurrence of SAEs, chronic illnesses, or other medically significant conditions. AEs were graded by the Investigator for severity and for relationship to the investigational product. Severity was graded according to a protocol-defined toxicity grading scale, adapted from toxicity grading scales used in similar trials (**Protocol S1**). AEs were coded using the Medical Dictionary for Regulatory Activities (MedDRA) and reported using System Organ Class and Preferred Term.

**Table 1:** Incidence of adverse events during the 28 days following CHMI.

		Highest Grade			
Adverse Event	N (%)	Mild	Moderate	Severe^1^	Mean duration^2^ days (range)
**Solicited Local** 3					
Pruritus	3 (50)	2	1		3.3 (2–5)
Pain	1 (16.7)	1			1 (1–1)
**Solicited Systemic** 4					
Abdominal Pain	3 (50)	3			1.25 (1–2)
Arthralgia	4 (66.7)	4			2 (1–3)
Chills	3 (50)	1	1	1	1.67 (1–2)
Diarrhea	1 (16.7)	1			1 (1–1)
Fever	4 (66.7)	3	1		1 (1–1)
Headache	5 (83.3)	4		1	2 (1–4)
Low Back Pain	2 (33.3)		2		6.5 (4–9)
Malaise	4 (66.7)	3		1	2.25 (1–6)
Myalgia	5 (83.3)	2	2	1	2.8 (1–4)
Nausea	4 (66.7)	1	3		1.5 (1–2)
Vomiting	1 (16.7)	0	1		1 (1–1)
Chest Pain	0 (0)				0 (0)
**Unsolicited** 5					
Decreased Appetite	1 (16.7)	1			4 (4–4)
Dizziness	1 (16.7)	1			2 (2–2)
Insomnia	1 (16.7)	1			1 (1–1)
Cough	2 (33.3)	2			3 (3–3)
Exertional Dyspnea	1 (16.7)	1			1(1–1)

1 A single subject accounted for all severe AEs which appeared on the day of positive blood smear, and all of which decreased in severity within 24 hours of treatment.

2 Per episode (number of episodes/subjects reporting episodes).

3 Only symptoms reported from day 0 through day 5 are included.

4 Only symptoms reported from day 6 through day 28 and determined by the Investigator to be malaria-related are included.

5 Collected throughout the 56 day study.

### Malaria diagnosis

Development of patent parasitemia was monitored by microscopic evaluation of peripheral blood smears. All subjects undergoing CHMI were expected to develop patent parasitemia. Blood smears were assessed by microscopy daily from Day 5 post-infection through diagnosis and after treatment until three consecutive daily blood smears were negative. Additional blood smears were prepared at other times as needed for diagnosis in symptomatic subjects. The prepatent period was defined as the time from CHMI to microscopic diagnosis, whereas the incubation period was the time from CHMI to the onset of malaria-related symptoms.

Malaria microscopy was conducted according to standard challenge microscopy procedures adapted from WRAIR. Giemsa-stained thick blood smears were prepared in duplicate from 10 µL of blood sample spread over a 1×2 cm rectangle and examined under a high power oil immersion objective (100X) by microscopists certified to read thick blood smears for CHMI studies. Smears were considered positive if at least two unambiguous parasites per slide were identified by a study microscopist and confirmed by the lead microscopist. For asymptomatic or treated subjects, up to five passes were read for an area equivalent to ∼290–320 total high-power fields (hpf), allowing for detection of a parasite density of approximately three parasites/µL before declaring a blood smear negative. For symptomatic volunteers, up to 15 passes (∼870–960 hpf) were scanned before declaring a smear to be negative. Quantification of microscopic parasite densities on slides from the day of diagnosis was performed by two independent microscopists who examined a minimum total of 1 µL of blood for each subject. Average parasitemia density was calculated from the number of parasites observed divided by the volume of blood examined microscopically (using conversion of volume per hpf) (**Table S1**).

At the conclusion of the trial, blood samples were tested using a first-generation *P. falciparum* 18S rRNA qRT-PCR assay by the University of Washington Department of Laboratory Medicine as described [16]. Samples tested by qRT-PCR included whole blood obtained twice daily from Day 5 until the first positive blood smear as well as blood collected daily after the first positive blood smear until hotel discharge and a final sample at Day 28. Briefly, 50 µL aliquots of whole blood were preserved in 2 mL of NucliSens lysis buffer (bioMérieux) and stored at −80°C until testing. Samples were extracted on a semi-automated instrument (EasyMag, bioMérieux), and total nucleic acids were subjected to qRT-PCR to detect the *P. falciparum* A-type 18S rRNA and a competitive internal control [16]. The limit of quantification for the qRT-PCR assay was 20 parasites/mL of whole blood; some samples below this threshold could be classified as ‘low positive’ by melting curve analysis as described [16]. Time to detection of parasites by molecular means was calculated from day of challenge to the day that parasites were detected quantitatively by qRT-PCR at ≥20 parasites/mL.

### Immune responses

Blood sampling for immunology endpoints occurred prior to challenge (baseline) and at regular intervals after challenge including Day 1 and Day 5 (corresponding to the liver stage of parasite development), the day of the first positive blood smear (corresponding to the blood stage) and post-treatment at Days 35 and 56 following challenge. In addition, subjects had the option to provide separate written consent to participate in a long-term immunology follow-up assessment with collection of serum samples on at three and six months post-challenge.

Levels of antibodies against liver- and blood-stage antigens including circumsporozoite protein (CSP), apical membrane antigen 1 (AMA-1) and the 42 kDa fragment of merozoite surface protein 1 (MSP-1_42_) were evaluated by enzyme-linked immunosorbent assay (ELISA) as described [17]. The antigens were produced by the National Institutes of Health Malaria ELISA Laboratory. ELISA plates (Immulon 4 HBX microtiter plates, Thermo Scientific) were coated with the above antigens (10 µg per complete 96-well plate), blocked with a non-reactive blocking buffer (5% non-fat dry milk) and incubated with 100 µL of volunteer samples or malaria-positive sera (pool of 20 sera from malaria-exposed Tanzanian adults) or malaria-naïve sera (pool of 16 sera from malaria-naïve US donors) for two hours at room temperature; all sera were pre-diluted to 1∶200 in 5% milk. Negative controls consisted of two wells per plate that did not receive any sera, but were otherwise subjected to all other ELISA steps. Overall ELISA results were accepted if the negative control wells had an average optical density (OD) ≤0.1 with the %CV ≤30% and positivity cutoffs were determined as described [17]. Individual samples were retested with dilution if ODs exceeded 3.5 or if the %CV exceeded 30% for a given sample.

Cell-mediated immune responses to CSP and liver-stage antigen 1 (LSA-1) were evaluated by interferon-gamma (IFNγ) ELISpot assay using peripheral blood mononuclear cells (PBMCs) as previously described [18]. Briefly, MultiScreen IP filter plates (Millipore) were coated overnight with mouse anti-human IFNγ capture antibody (clone 1-D1K, 10 µg/mL, Mabtech), washed and blocked. PBMCs (200,000/well) were incubated with media only (negative control), CSP- or LSA-1-derived peptide pools (1 µg/mL), cytomegalovirus (CMV) peptide controls (1 µg/mL) or phytohemagglutinin (PHA, 1 µg/mL, positive control) in 125 µL RPMI supplemented with 10% fetal bovine serum, 2 mM L-glutamine, 100 U/mL penicillin and 100 µg/mL streptomycin for 18–22 hours at 37°C. Media only wells (no cells) were used as a plate control. After incubation, plates were washed and incubated for 2–4 hours with biotinylated mouse anti-human IFNγ antibody (clone 7-b6-1, 1 µg/mL, Mabtech) at room temperature. Plates were again washed and incubated with alkaline-phosphatase-conjugated anti-biotin antibody (1∶750, Vector Labs) for 2–3 hours at room temperature. Plates were developed for 5–10 min using NBT/BCIP (nitro blue tetrazolium/5-bromo-4-chloro-3-indolyl-phosphate) substrate according to manufacturer instructions (Pierce). Plates were dried overnight and counted on an ImmunoSpot reader (C.T.L., Shaker Heights, OH). Sample results were accepted if the mean negative control wells spot count for a given sample was ≤20 spots/well, if the PHA control was ≥400 spots/well and if the mean spot count in media only wells (no cells) per plate was ≤6 spots/well. Positivity criteria was ≥55 spots per 10^6^ PBMCs and ≥4-fold above the mean of the negative control wells as described [18], [19].

### Statistics

The study was an open-label single intervention trial to demonstrate the reproducibility of CHMI at a new center. Primary and secondary analyses involved summaries and descriptive statistics for safety, efficacy (infectivity) and immunology endpoints. Comparative analyses of the primary (microscopy) and exploratory (qRT-PCR) infectivity endpoints was undertaken for the safety population and is presented as the difference in the cumulative distribution of the number of days between challenge and the first positive test (blood smear minus qRT-PCR) using a Kaplan-Meier estimator. Analyses of immunology endpoints (by ELISA and ELISpot) were performed for all subjects for whom data was available at a specific time point. Qualitative assay data analysis was performed by tabulating the frequency of positive responses for each assay at each timepoint an assessment was performed.

## Results

### Subject disposition

A total of 18 subjects were screened from January – March 2010 for eligibility to enroll in the study (**Figure 1**). Seven subjects were screen failures based on eligibility criteria, four subjects withdrew consent and six of the remaining seven eligible subjects were enrolled in the trial, including four male and two female subjects aged 19–28 years (mean 23.5 years). All six subjects were included in the safety and efficacy analyses. No subject discontinued the trial. All six subjects completed the trial through the six month follow up phone call. Additionally, five of the six subjects consented to participate in the optional blood draws for immunology samples at 3 and 6 months post-challenge.

### Malaria challenge

Six subjects successfully completed the experimental infection, receiving a minimum of five invective bites. One subject inadvertently received a total of six infective bites, rather than the five defined in the protocol. This deviation was reviewed by the Medical Monitor and Safety Monitoring Committee. After reviewing safety data, both agreed that the additional infective bite did not put the subject at increased risk. As noted in **Table 2**, all subjects required more than one feeding iteration in order to receive a total of five infective mosquito bites. The average number of feeding iterations was 4.6 (range 2–7). In this initial study, the overall prevalence of sporozoite containing mosquitoes was 50% (**Figure S1**).

**Table 2:** Subject infectivity summary.

Subject ID	Feeding Iterations^1^	Time to qRT-PCR positive^2^ (days)	Prepatent period^3^ (days)	Incubation period^4^ (days)	Peak parasite density by method^5^ (parasites/mL)
A	4	7.0	11.0	7	9460 RTPCR
					12450 BS
B	2	7.0	11.0	12	50170 RTPCR
					53700 BS
C	4	7.0	11.0	8	120680 RTPCR
					39000 BS
D	7	7.0	11.0	11	5350 RTPCR
					7270 BS
E	4	7.0	9.0	6	3730 RTPCR
					5150 BS
F	7	10.0	14.0	14	12880 RTPCR
					2330 BS
Mean	4.7	7.5	11.2	9.7	33712 RTPCR
					19983 BS
Median	4	7.0	11	9.5	11170 RTPCR
					9860 BS
SD	2.0	1.2	1.6	3.1	45956 RTPCR
					21206 BS

1 Number of rounds of mosquito feeding exposures required to achieve a total of 5 infective bites, as demonstrated by evidence of a blood meal in the mosquito abdomen and a post-feed sporozoite salivary gland score of +2 or higher. Subject D received a total of 6 infective bites while all other subjects received 5 bites.

2 Number of days from CHMI to qRT-PCR-positive.

3 Number of days from CHMI to peripheral blood smear-positive.

4 Number of days from CHMI to symptomatic.

5 BS, blood smear.

### Safety

**Figure 2:**
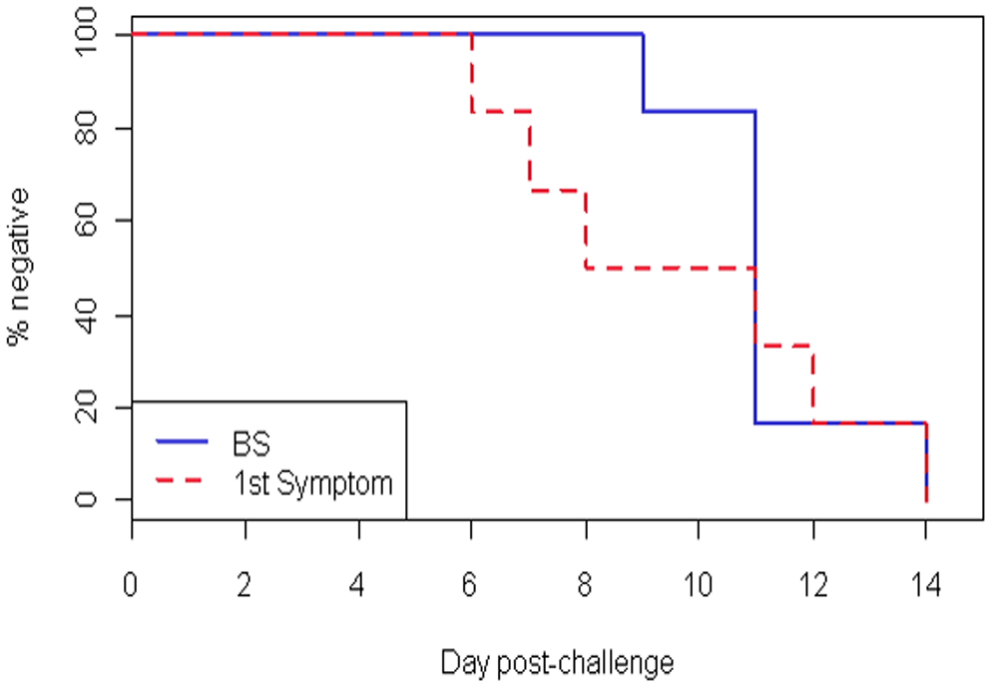
Comparison of prepatent and incubation periods. Kaplan-Meier survival curve showing the percentage of subjects without patent parasitemia by blood smear (blue line) or without symptoms (dashed red line) following challenge.

No SAEs were reported for any subject through Day 56 of the trial or during six months following the CHMI. The incidence of solicited and unsolicited AEs reported as related to the malaria challenge or infection is summarized in **Table 1**. The mean incubation period (time to first malaria symptom) was 9.7 days (range 6–14 days; 95% CI 6.4–13.0 days; **Table 2**). Three of six subjects (50%) experienced at least one post-challenge local and/or systemic reactogenicity symptom between the day of challenge (Day 0) and Day 5 post-CHMI. Similarly, 50% of subjects reported at least one symptom of clinical malaria prior to the diagnosis of patent parasitemia. Two subjects (33.3%) had onset of clinical symptoms on the same day of blood smear diagnosis and one subject (16.7%) had no systemic symptoms until after diagnosis of patent parasitemia (**Figure 2**).

During the acute reactogenicity period (Day 0 to Day 5 post-challenge), the most common AE was pruritus localized to the site of inoculation, occurring in 3 (50%) subjects. No other AE occurred in >1 subject during this period. All subjects experienced at least one systemic AE during the 28 days post-challenge. The most frequently reported systemic AEs related to malaria were headache and myalgia each occurring in 5 (83.3%) subjects, followed by fever, malaise, nausea and arthralgia each occurring in 4 (66.7%) subjects (**Table 1**). Most AEs were classified as mild or moderate in severity. One subject developed severe chills, headache, malaise and myalgia on the day of first positive blood smear. The symptoms peaked in severity on the day of diagnosis and decreased in severity within 24 hours of treatment initiation. The constellation of symptoms in this subject was consistent with uncomplicated clinical malaria, and all symptoms resolved within 48–72 hours of completion of treatment.

All subjects reported at least one unsolicited AE. The majority of unsolicited AEs were mild, and most were considered unrelated to challenge procedures or malaria infection. Related events included cough, decreased appetite, dizziness, insomnia and exertional dyspnea (**Table 1**). Of these, only cough (2/6) was reported by more than one subject. All AEs resolved by Day 56. Subjects were followed for six months post-CHMI, and study follow-up ended in September 2010.

Four of six subjects had no laboratory abnormalities throughout the trial. One subject had an asymptomatic mild-moderate increase in liver transaminases (ALT 160 U/L [2.5 xULN] and AST 105 U/L [2.6 x ULN)] occurring on the same day as diagnosis of patent parasitemia (Day 11 post-challenge). The other subject experienced a mild, asymptomatic decrease in Hb from baseline (1.8 g/dL) occurring on Day 10 post-challenge that remained within the normal range. Both abnormalities resolved by Day 35.

### Infectivity

All subjects undergoing CHMI developed microscopic parasitemia with a mean prepatent period of 11.2 days (range 9–14 days; SD 1.6 days) (**Table 2**). Four of six subjects (67%) had a prepatent period of 11 days. The earliest detection of patent parasitemia was on Day 9 post-challenge and the latest was at 14 days. As noted above, the mean incubation period was 9.7 days (range 7–14 days; SD 3.1 days). Similar to reports from other centers, there was no apparent correlation between prepatent and incubation periods (**Figure 2**).

### Parasitemia

The geometric mean parasite density (by microscopy) at the time of microscopic diagnosis was 10.8 parasites/µL across all slides examined (range 2–69 parasites/µL; SD 21.2 parasites/µL) (**Table S1**). Similar to reports from other centers, there was no apparent correlation between incubation or prepatent periods and density of parasitemia at diagnosis.

**Figure 3:**
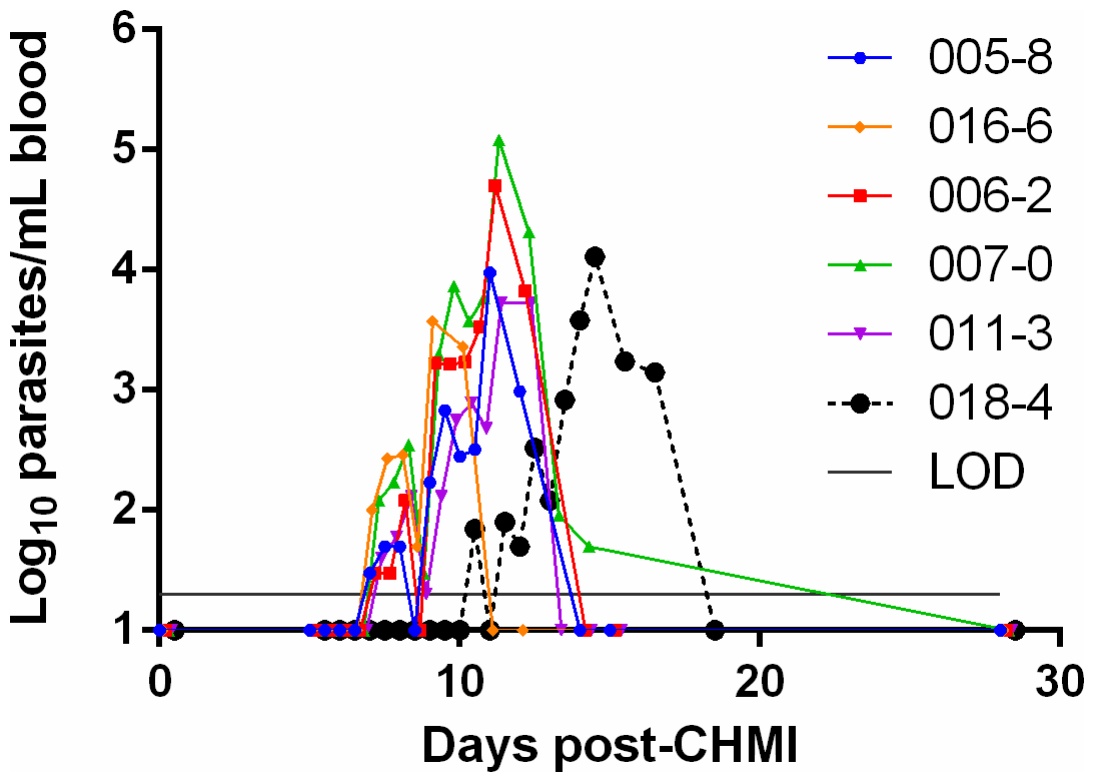
qRT-PCR-based course of parasitemia. Parasite density based on qRT-PCR measurements are presented individually for each participant.

We previously reported the performance characteristics of our qRT-PCR assay, and its comparability to microscopic diagnosis in this trial [16]. All subjects became qRT-PCR positive with a mean time to positivity of 7.5 days (range 7.0–10.0 days; SD 1.2 days) (**Table 2**). Detection of parasites by qRT-PCR occurred an average of 3.7 days (range 2.0–4.0 days; SD 0.8 days) earlier than by peripheral blood smears [16]. Five of six subjects (83%) were positive by qRT-PCR four days prior to blood smear diagnosis including four subjects who become qRT-PCR positive on Day 7 and one subject who became qRT-PCR positive on Day 10 (**Figure 3**). The sixth subject became positive by qRT-PCR on Day 7 post-CHMI, two days prior to blood smear diagnosis. For most individuals, the qRT-PCR findings were consistent with the expected timing for release of merozoites from the liver into the bloodstream. With the exception of one subject, qRT-PCR and microscopic parasite density measurements aligned within 0.5 log_10_ parasites/mL (**Table S2**). The parasite density by qRT-PCR on the day of corresponding microscopic diagnosis ranged from 3,730 to 120,700 parasites/mL, which was in agreement with reports from other CHMI centers [13].

Subjects were treated with chloroquine upon microscopic diagnosis and were released from the hotel after three consecutive negative daily blood smears. All subjects were blood smear-negative within two days of initiating treatment (**Table 3**, mean 1.67 days). As previously reported [16], except for one subject, qRT-PCR remained positive even after blood smears became negative after treatment. Two subjects were qRT-PCR-negative within two days of treatment, but the remaining subjects were still positive by qRT-PCR at the time of discharge from the hotel (**Table 3**). By five days post-treatment, the qRT-PCR signal was undetectable in three subjects and reduced by more than two orders of magnitude in the remaining three subjects. All subjects were negative by qRT-PCR at the Day 28 follow-up clinic visit.

**Table 3:** Time from anti-malarial treatment to clearance of peripheral parasitemia.

Subject	Time to first negative blood smear (days)	Time to first negative qRT-PCR (days)
A	2	*
B	2	*
C	1	*
D	2	2
E	1	2
F	2	4

*Data not available. Subjects were qRT-PCR positive and peripheral blood smear negative at the time of discharge from the hotel. No further daily sampling was performed after discharge until Day 28, at which point all subjects in the study were blood smear and qRT-PCR negative.

### Immunology

**Figure 4:**
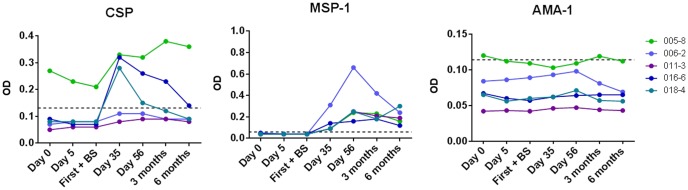
Humoral immune responses to *P. falciparum* antigens. ELISAs were performed on the indicated days post-CHMI to test for responses against the indicated *P. falciparum* antigens. The positivity cut-off (dotted line) was calculated per ELISA plate as three standard deviations above the mean of the two negative control wells. All samples with an OD higher than the calculated cut-off were deemed positive. Data are presented for the five subjects completing the follow up at 3 and 6 months. Data to Day 56 for the sixth subject did not differ considerably from the five subjects in the graph.

Humoral and cellular responses to *P. falciparum* antigens were analyzed for all six subjects through Day 56 and, for five of the six subjects who consented to the extension portion of the protocol, at three and six months post-challenge. Humoral responses to *P. falciparum* were measured by ELISA for pre-erythrocytic (CSP) and blood-stage (AMA-1 and MSP-1_42_) antigens (**Figure 4**). Two subjects initially seronegative for CSP became seropositive, and of these two, one remained seropositive at 6 months. One subject was seropositive to CSP at baseline and remained so throughout the 6 month study period; this could be due to cross-reactive response to epitopes shared between CSP and other common antigens or remote exposure to malaria, although this was not reported by the subject. All five subjects were seronegative to MSP-1_42_ at baseline, but became seropositive on Days 35 and remained so throughout the six month study period (**Figure 4**). No subject was seropositive to AMA-1 at any timepoint, except for one subject who exhibited low responses on Day 0 and at three months post-CHMI, reporting an average OD of 0.12 for both timepoints (positivity cut-off was 0.11).

**Figure 5:**
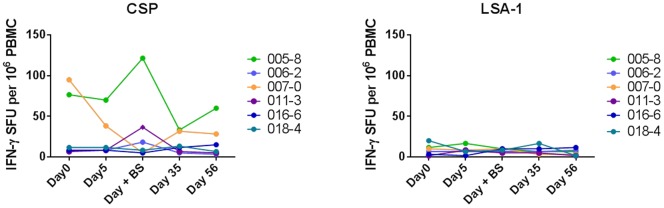
Cellular immune response to CSP and LSA-1. IFNγ ELISpot assays were performed using the indicated CSP and LSA-1 peptide pools on the indicated days post-CHMI. Spot forming units (SFU) per million PBMC are shown per individual subject for all six subjects.

Only one subject showed cellular antigen-specific cell-mediated immune responses to CSP, detected on the day of the first positive blood smear (**Figure 5**). The CSP-specific response was negative at Day 35 and Day 56. Responses to later timepoints were therefore anticipated to be negative. As a result, PBMC samples from extension visits were not tested by ELISpot in all subjects. For LSA-1, no positive responses were detected in any subject at any timepoint (**Figure 5**). The mean LSA-1 response was 7.78 spot forming units (SFU)/million PBMCs (95% CI 6.08–9.47 SFU/million), similar to the negative control wells (mean 8.5 SFU/million). Based on the lack of response seen by Day 56, the LSA-1 cellular responses at three months and six months were not evaluated.

## Discussion

Therapeutic and experimental malaria infection of humans has a long history, dating back nearly a century [20]–[22]. Chulay et al. first described the production of infected mosquitoes from *in vitro* cultured NF54 strain *P. falciparum* gametocytes for the experimental challenge of six healthy volunteers under controlled conditions [5]. Since that time, the CHMI mosquito bite model has continued to be an important and powerful tool in the clinical development path towards effective malaria vaccines and drugs. This study demonstrates that CHMI at the Seattle-based MCTC is safe, reproducible and well tolerated. Recent efforts at standardization of practices across centers [23] and compliance with phase-appropriate cGMP for the production of the infected mosquitoes ensures the continued safety and integrity of the CHMI model and allows for comparability in data sets across multiple centers. To this end, this is the first CHMI study conducted under an IND where the *P. falciparum* sporozoite was considered an investigational product. Similarly, efforts to align and standardize supportive diagnostic tools such as qRT-PCR [24] will further ensure comparability of data between centers.

Similar to reports from other centers, the local, systemic and laboratory AEs observed following CHMI in this study were consistent with mosquito exposure and subsequent uncomplicated clinical malaria episodes. Solicited adverse events peaked within 48 hours of blood smear diagnosis and most resolved within 72 hours. Severe symptoms (chills, headache, malaise, myalgia) observed in one subject were likewise consistent with uncomplicated malaria and resolved within 24 hours of initiating antimalarial treatment.

Consistent with the literature [13], all subjects developed signs and symptoms of malaria infection during the trial. Both the mean incubation and prepatent periods (9.7 days and 11.2 days, respectively) were consistent with those reported in other CHMI studies (8.8 and 11.5 days) [1], [3], [4], [6], and there appeared to be no significant relationship between incubation and prepatent periods and parasitemia upon diagnosis. Half of the subjects reported clinical symptoms of malaria prior to blood smear diagnosis, one remained asymptomatic until the first day of treatment and the remaining two had symptoms concurrent with blood smear diagnosis, which was generally consistent with the published literature [1], [6]. Similarly, there was no correlation between peak parasitemia or duration of parasitemia and severity of clinical illness.

In this study, prepatent parasitemia was detected by qRT-PCR an average of 3.7 days before blood smear diagnosis, and modeling of the qRT-PCR data suggested that 28–60 (range 8–111) hepatocytes were infected per subject as previously reported [16]. In addition, there was good quantitative agreement between qRT-PCR- and microscopy-derived parasite density results on the day of patency. This study was the first to demonstrate accurate parasite detection and quantification by this qRT-PCR method. Because the 18S rRNA is biologically enriched in malaria-infected erythrocytes relative to the parent 18S rDNA by a factor of 3500–5000 [16], [25], the qRT-PCR format offers robust sensitivity relative to qPCR for samples of the same volume.

In this study, mosquito infectivity was approximately 50% as assessed by the post-challenge dissection and sporozoite rating system [5], and multiple mosquito bite iterations were required to achieve a total of five infectious bites. Ultimately, 100% of subjects were infected and developed patent parasitemia. Mosquito infectivity has since been optimized, and in Seattle BioMed trials since this study, 81–91% of mosquitoes had sporozoite ratings of 2+ or higher (Jen Hume, personal communication). High sporozoite loads have also been recently noted for aseptic cGMP-grade mosquitoes used to induce infections with less than the standard five bites [3].

As expected from the limited antigenic exposure with only five to six infected mosquito bites, limited humoral and cellular immune responses to pre-erythrocytic and erythrocytic antigens were observed. Although two subjects had transient measurable antibody levels to CSP, neither maintained a detectable response at six months post-challenge. A third subject had pre-existing humoral and cellular responses to CSP that persisted at all timepoints, did not markedly increase after infection and remains unexplained; anomalous cross-reactive responses to CSP have also been reported by others [26].

Likewise, a single challenge with *P. falciparum* in our study was not sufficient to induce significant antibody response to AMA-1 as detected by our standardized assay. The peak cellular response was expected at Day 35+/−3 days, as previously reported in experimental malaria infection models [27]. In this study, cellular responses to pre-erythrocytic antigens were limited, except for two subjects with pre-existing responses to CSP that persisted at many timepoints. For CSP, there was no difference between timepoints when all subjects were analyzed together. Only one subject showed new antigen-specific responses to CSP, detected on the day of the first positive blood smear but not on Day 35 or 56. For LSA-1, no positive responses were detected in any subject at any timepoint.

In summary, this study demonstrates the successful implementation of the CHMI mosquito bite model at Seattle BioMed, thereby expanding the global capacity for conducting these important studies. Subjects were successfully infected with *P. falciparum* sporozoites, and all subjects developed patent parasitemia and were successfully treated and cured with standard doses of chloroquine. The CHMI was safe and well tolerated. The AE profile, prepatent and incubation periods were consistent with the expected clinical course and response to CHMI reported in previous malaria studies conducted at other centers worldwide. All volunteers developed antibody responses to the blood-stage antigen MSP-1, which was detectable up to six months post-challenge. Both humoral and cellular responses to pre-erythrocytic antigens CSP and LSA-1 were limited. Results from a diagnostic qRT-PCR assay correlated with blood smear density results and identified prepatent infection nearly four days before peripheral blood smears. Seattle BioMed plans to support additional CHMI studies to accelerate development of effective malaria drugs and vaccines.

## Supporting Information

Figure S1 **Cumulative mosquito sporozoite ratings for all mosquitoes used to challenge each subject.** The sporozoite load was rated microscopically according to a semi-quantitative scale: 0 (no sporozoites observed), +1 (1–10), +2 (11–100), +3 (101–1000) and +4 (>1000) [5]. Only mosquitoes with a salivary gland rating of +2 or greater were considered infective.
(TIF)

Table S1 **Microscopy-based parasite density assessment.** ^1^Volume per 1 cm pass based on the field number for each microscope, corresponding to 290–320 hpf. Microscope/equipment numbers: Nikon Eclipse 50i, (FN 22), Nikon microscope E200, (FN20)
(DOCX)

Table S2 **Comparison of microscopy and qRT-PCR-based parasite density assessments for first smear-positive sample.** ^1^qRT-PCR measurements as reported in [1] (converted from parasites/mL to parasites/µL). ^2^Quantitative agreement denoted if microscopic and qRT-PCR parasite density measurements were within 0.5 log_10_ parasites/mL of each other.
(DOCX)

Checklist S1 **CONSORT checklist for MC-001.**
(DOC)

Protocol S1 **IRB-approved study MC-001 protocol.**
(PDF)

## References

[1] Church LW, Le TP, Bryan JP, Gordon DM, Edelman R, et al (1997) Clinical manifestations of Plasmodium falciparum malaria experimentally induced by mosquito challenge. J Infect Dis 175: 915–920. 9086149 10.1086/513990

[2] Roestenberg M, McCall M, Hopman J, Wiersma J, Luty AJ, et al (2009) Protection against a malaria challenge by sporozoite inoculation. N Engl J Med 361: 468–477. 19641203 10.1056/NEJMoa0805832

[3] Lyke KE, Laurens M, Adams M, Billingsley PF, Richman A, et al (2010) Plasmodium falciparum malaria challenge by the bite of aseptic Anopheles stephensi mosquitoes: results of a randomized infectivity trial. PLoS One 5: e13490. 21042404 10.1371/journal.pone.0013490 PMC2958836

[4] Verhage DF, Telgt DS, Bousema JT, Hermsen CC, van Gemert GJ, et al (2005) Clinical outcome of experimental human malaria induced by Plasmodium falciparum-infected mosquitoes. Neth J Med 63: 52–58. 15768480

[5] Chulay JD, Schneider I, Cosgriff TM, Hoffman SL, Ballou WR, et al (1986) Malaria transmitted to humans by mosquitoes infected from cultured Plasmodium falciparum. Am J Trop Med Hyg 35: 66–68. 3511753 10.4269/ajtmh.1986.35.66

[6] Epstein JE, Rao S, Williams F, Freilich D, Luke T, et al (2007) Safety and clinical outcome of experimental challenge of human volunteers with Plasmodium falciparum-infected mosquitoes: an update. J Infect Dis 196: 145–154. 17538895 10.1086/518510

[7] Hoffman SL (1997) Experimental challenge of volunteers with malaria. Ann Intern Med 127: 233–235. 9245231 10.7326/0003-4819-127-3-199708010-00010

[8] Spring M, Polhemus M, Ockenhouse C (2014) Controlled human malaria infection. J Infect Dis 209 Suppl 2S40–45. 24872394 10.1093/infdis/jiu063

[9] Herrera S, Solarte Y, Jordan-Villegas A, Echavarria JF, Rocha L, et al (2011) Consistent safety and infectivity in sporozoite challenge model of Plasmodium vivax in malaria-naive human volunteers. Am J Trop Med Hyg 84: 4–11. 21292872 10.4269/ajtmh.2011.09-0498 PMC3032484

[10] Herrera S, Fernandez O, Manzano MR, Murrain B, Vergara J, et al (2009) Successful sporozoite challenge model in human volunteers with Plasmodium vivax strain derived from human donors. Am J Trop Med Hyg 81: 740–746. 19861603 10.4269/ajtmh.2009.09-0194 PMC2826834

[11] Douglas AD, Edwards NJ, Duncan CJ, Thompson FM, Sheehy SH, et al (2013) Comparison of modeling methods to determine liver-to-blood inocula and parasite multiplication rates during controlled human malaria infection. J Infect Dis 208: 340–345. 23570846 10.1093/infdis/jit156 PMC3685228

[12] Bejon P, Andrews L, Andersen RF, Dunachie S, Webster D, et al (2005) Calculation of liver-to-blood inocula, parasite growth rates, and preerythrocytic vaccine efficacy, from serial quantitative polymerase chain reaction studies of volunteers challenged with malaria sporozoites. J Infect Dis 191: 619–626. 15655787 10.1086/427243

[13] Roestenberg M, O'Hara GA, Duncan CJ, Epstein JE, Edwards NJ, et al (2012) Comparison of clinical and parasitological data from controlled human malaria infection trials. PLoS One 7: e38434. 22701640 10.1371/journal.pone.0038434 PMC3372522

[14] Ballou WR, Arevalo-Herrera M, Carucci D, Richie TL, Corradin G, et al (2004) Update on the clinical development of candidate malaria vaccines. Am J Trop Med Hyg 71: 239–247. 15331843

[15] Gaziano TA, Young CR, Fitzmaurice G, Atwood S, Gaziano JM (2008) Laboratory-based versus non-laboratory-based method for assessment of cardiovascular disease risk: the NHANES I Follow-up Study cohort. Lancet 371: 923–931. 18342687 10.1016/S0140-6736(08)60418-3 PMC2864150

[16] Murphy SC, Prentice JL, Williamson K, Wallis CK, Fang FC, et al (2012) Real-time quantitative reverse transcription PCR for monitoring of blood-stage Plasmodium falciparum infections in malaria human challenge trials. Am J Trop Med Hyg 86: 383–394. 22403305 10.4269/ajtmh.2012.10-0658 PMC3284350

[17] Miura K, Orcutt AC, Muratova OV, Miller LH, Saul A, et al (2008) Development and characterization of a standardized ELISA including a reference serum on each plate to detect antibodies induced by experimental malaria vaccines. Vaccine 26: 193–200. 18054414 10.1016/j.vaccine.2007.10.064 PMC2253722

[18] McElrath MJ, De Rosa SC, Moodie Z, Dubey S, Kierstead L, et al (2008) HIV-1 vaccine-induced immunity in the test-of-concept Step Study: a case-cohort analysis. Lancet 372: 1894–1905. 19012957 10.1016/S0140-6736(08)61592-5 PMC2774110

[19] Dubey S, Clair J, Fu TM, Guan L, Long R, et al (2007) Detection of HIV vaccine-induced cell-mediated immunity in HIV-seronegative clinical trial participants using an optimized and validated enzyme-linked immunospot assay. J Acquir Immune Defic Syndr 45: 20–27. 17310936 10.1097/QAI.0b013e3180377b5b

[20] James SP, Shute PG (1926) Malaria Commission: report on the first results of laboratory work on malaria in England. Geneva: League of Nations Health Organization.

[21] Nicol WD (1927) The care and management of induced malaria. J Ment Sci 6: 1–9.

[22] James SP (1931) Some general results of a study of induced malaria in England. Trans R Soc Trop Med Hyg 24: 477–538.

[23] Laurens MB, Duncan CJ, Epstein JE, Hill AV, Komisar JL, et al (2012) A consultation on the optimization of controlled human malaria infection by mosquito bite for evaluation of candidate malaria vaccines. Vaccine 30: 5302–5304. 22659449 10.1016/j.vaccine.2012.04.088

[24] Murphy SC, Hermsen CC, Douglas AD, Edwards N, Petersen I, et al (2014) External quality assurance of malaria nucleic acid testing for clinical trials and eradication surveillance. PLoS One 9: e97398. 24838112 10.1371/journal.pone.0097398 PMC4023973

[25] Murphy SC, Daza G, Chang M, Coombs R (2012) Laser cutting eliminates nucleic acid cross-contamination in dried-blood-spot processing. J Clin Microbiol 50: 4128–4130. 23052309 10.1128/JCM.02549-12 PMC3503017

[26] Zevering Y, Amante F, Smillie A, Currier J, Smith G, et al (1992) High frequency of malaria-specific T cells in non-exposed humans. Eur J Immunol 22: 689–696. 1547814 10.1002/eji.1830220311

[27] Todryk SM, Walther M, Bejon P, Hutchings C, Thompson FM, et al (2009) Multiple functions of human T cells generated by experimental malaria challenge. Eur J Immunol 39: 3042–3051. 19658096 10.1002/eji.200939434

