# Supplementary figures and images for: Safety and Comparability of Controlled Human Plasmodium falciparum Infection by Mosquito Bite in Malaria-Naïve Subjects at a New Facility for Sporozoite Challenge
Source: PLoS One. 2014 Nov 18;9(11):e109654. doi: 10.1371/journal.pone.0109654 (PMC4236046; doi:10.1371/journal.pone.0109654)

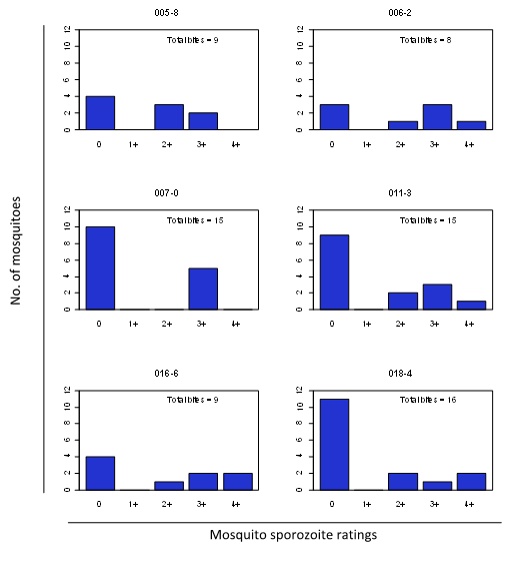

Supplement: Figure S1 — Cumulative mosquito sporozoite ratings for all mosquitoes used to challenge each subject. The sporozoite load was rated microscopically according to a semi-quantitative scale: 0 (no sporozoites observed), +1 (1–10), +2 (11–100), +3 (101–1000) and +4 (>1000) [5]. Only mosquitoes with a salivary gland rating of +2 or greater were considered infective. (TIF) [file pone.0109654.s001.tif]
