# Supplementary material for: Safety and Comparability of Controlled Human Plasmodium falciparum Infection by Mosquito Bite in Malaria-Naïve Subjects at a New Facility for Sporozoite Challenge
Source: PLoS One. 2014 Nov 18;9(11):e109654. doi: 10.1371/journal.pone.0109654 (PMC4236046; doi:10.1371/journal.pone.0109654)
Supplement: Table S1 — Microscopy-based parasite density assessment. 1Volume per 1 cm pass based on the field number for each microscope, corresponding to 290–320 hpf. Microscope/equipment numbers: Nikon Eclipse 50i, (FN 22), Nikon microscope E200, (FN20) (DOCX) [file pone.0109654.s002.docx]

| **Subject PTID** | **Day post-challenge** | **Parasites counted per pass** | | | | | | | | | | | | | | | **Total parasites counted** | **Passes read** | **Calculated blood volume read (μL)^1^** | **Parasites /μL** | **Parasites/μl (avg)** |
| --- | --- | --- | --- | --- | --- | --- | --- | --- | --- | --- | --- | --- | --- | --- | --- | --- | --- | --- | --- | --- | --- |
|  |  | **1** | **2** | **3** | **4** | **5** | **6** | **7** | **8** | **9** | **10** | **11** | **12** | **13** | **14** | **15** |  | **(1 cm)** |  |  |  |
| 016-6 | 9 | 1 | 2 | 0 | 0 | 0 |  |  |  |  |  |  |  |  |  |  | 3 | 5 | 0.55 | 5.5 | **5.2** |
|  |  | 0 | 0 | 0 | 0 | 1 |  |  |  |  |  |  |  |  |  |  | 1 | 5 | 0.5 | 2.0 |  |
|  |  | 1 | 0 | 2 | 1 | 0 |  |  |  |  |  |  |  |  |  |  | 4 | 5 | 0.5 | 8.0 |  |
| 011-3 | 11 | 1 | 2 | 1 | 1 | 0 |  |  |  |  |  |  |  |  |  |  | 5 | 5 | 0.55 | 9.1 | **7.3** |
|  |  | 0 | 1 | 3 | 0 | 0 |  |  |  |  |  |  |  |  |  |  | 4 | 5 | 0.5 | 8.0 |  |
|  |  | 1 | 0 | 0 | 1 | 0 |  |  |  |  |  |  |  |  |  |  | 2 | 5 | 0.5 | 4.0 |  |
|  |  | 2 | 0 | 0 | 0 | 2 |  |  |  |  |  |  |  |  |  |  | 4 | 5 | 0.5 | 8.0 |  |
| 007-0 | 11 | 6 | 3 | 3 | 4 | 6 |  |  |  |  |  |  |  |  |  |  | 22 | 5 | 0.55 | 40.0 | **39.0** |
|  |  | 4 | 3 | 5 | 4 | 3 |  |  |  |  |  |  |  |  |  |  | 19 | 5 | 0.5 | 38.0 |  |
| 005-8 | 11 | 0 | 1 | 3 | 1 | 1 |  |  |  |  |  |  |  |  |  |  | 6 | 5 | 0.55 | 10.9 | **12.5** |
|  |  | 3 | 0 | 1 | 1 | 2 |  |  |  |  |  |  |  |  |  |  | 7 | 5 | 0.5 | 14.0 |  |
| 006-2 | 11 | 7 | 12 | 3 | 9 | 7 |  |  |  |  |  |  |  |  |  |  | 38 | 5 | 0.55 | 69.1 |  |
|  |  | 6 | 3 | 3 | 3 | 3 |  |  |  |  |  |  |  |  |  |  | 18 | 5 | 0.5 | 36.0 | **53.7** |
|  |  | 3 | 4 | 6 | 8 | 7 |  |  |  |  |  |  |  |  |  |  | 28 | 5 | 0.5 | 56.0 |  |
| 018-4 | 14 | 1 | 0 | 0 | 0 | 0 | 0 | 0 | 0 | 1 | 1 | 0 | 0 | 0 | 0 | 1 | 4 | 15 | 1.5 | 2.7 | **2.3** |
|  |  | 0 | 0 | 0 | 1 | 0 | 0 | 0 | 1 | 0 | 1 | 0 | 0 | 0 | 0 | 0 | 3 | 15 | 1.5 | 2.0 |  |
