# Supplementary material for: Safety and Comparability of Controlled Human Plasmodium falciparum Infection by Mosquito Bite in Malaria-Naïve Subjects at a New Facility for Sporozoite Challenge
Source: PLoS One. 2014 Nov 18;9(11):e109654. doi: 10.1371/journal.pone.0109654 (PMC4236046; doi:10.1371/journal.pone.0109654)
Supplement: Table S2 — Comparison of microscopy and qRT-PCR-based parasite density assessments for first smear-positive sample. 1qRT-PCR measurements as reported in [1] (converted from parasites/mL to parasites/µL). 2Quantitative agreement denoted if microscopic and qRT-PCR parasite density measurements were within 0.5 log10 parasites/mL of each other. (DOCX) [file pone.0109654.s003.docx]

| Subject PTID | By microscopy (parasites/μL) | By qRT-PCR (parasites/μL)^1^ | By microscopy (log_10_ para/mL) | By qRT-PCR (log_10_ para/mL) | Microscopy vs. qRT-PCR agreement^2^ |
| --- | --- | --- | --- | --- | --- |
|  |  |  |  |  | (Difference in log_10_ para/mL) |
| 016-6 | 5.2 | 3.73 | 3.65 | 3.57 | Yes (0.08) |
| 011-3 | 7.3 | 5.35 | 3.84 | 3.73 | Yes (0.11) |
| 007-0 | 39.0 | 120.70 | 4.59 | 5.08 | Yes (0.49) |
| 005-8 | 12.5 | 9.50 | 4.09 | 3.98 | Yes (0.12) |
| 006-2 | 53.7 | 50.20 | 4.71 | 4.70 | Yes (0.01) |
| 018-4 | 2.3 | 12.90 | 3.36 | 4.11 | No (0.75) |
