# Supplementary material for: Safety and Comparability of Controlled Human Plasmodium falciparum Infection by Mosquito Bite in Malaria-Naïve Subjects at a New Facility for Sporozoite Challenge
Source: PLoS One. 2014 Nov 18;9(11):e109654. doi: 10.1371/journal.pone.0109654 (PMC4236046; doi:10.1371/journal.pone.0109654)
Supplement: Protocol S1 — IRB-approved study MC-001 protocol. (PDF) [file pone.0109654.s005.pdf]

CLINICAL TRIAL PROTOCOL

**Title:**	**Reproducibility of Malaria Challenge in Healthy Volunteers**
**Protocol No.:**	MC-001
**Protocol Amendment No.**	**1**
**Version number and date:**	2.0 26 March 2010
**Principal Investigator:**	Angela K. Talley, MD
**Sponsor:**	Seattle Biomedical Research Institute<br>307 Westlake Avenue N Suite 500<br>Seattle, WA 98109-5219<br>Telephone: 206-256-7200
**Funding Sponsor:**	The PATH Malaria Vaccine Initiative
**IND No.:**	14224

CONFIDENTIALITY STATEMENT

This protocol contains privileged and or proprietary information, which is the property of Seattle Biomedical Research Institute (Seattle BioMed). Accordingly, this protocol is to be treated as confidential and must not be disclosed or used except as authorized in writing by Seattle BioMed.

KEY ROLES AND CONTACTS

Investigator:

Angela K. Talley, MD
Lead Trials Investigator
Malaria Clinical Trials Center
Seattle Biomedical Research Institute
Clinical Instructor of Medicine, Division of Allergy and Infectious Diseases, University of Washington
307 Westlake Avenue North
Seattle, WA, 98109
Phone: (206) 256-7131
Fax: (206) 256-7229
email: angela.talley@sbri.org

Other Institutions: University of Washington

Clinical Laboratories: Quest Diagnostics

Sponsor Medical Monitor:

Wesley VanVoorhis, MD, PhD
Head, Allergy and Infectious Diseases Division
Professor of Medicine, Adjunct Professor, Microbiology & Global Health
University of Washington
1959 NE Pacific Ave, HSB BB-1237, MS 356523
Seattle, WA 98195-7185
Phone: (206) 543-2447 (office)
Fax (206) 616-4898
email: wesley@u.washington.edu

Sponsor Representative and Study Monitor:

Susan Lundebjerg
Quality Assurance Analyst
Seattle Biomedical Research Institute
307 Westlake Ave N
Seattle, WA 98109
Phone: (206) 256-7125
Fax: (206) 256-7229
email:susan.lundebjerg@sbri.org

Funding Sponsor Representative:

Cynthia K. Lee, Ph. D.
Scientific Advisor
The PATH Malaria Vaccine Initiative
7500 Old Georgetown Road, Suite 1200
Bethesda, MD 20814 USA
Office: +1.240.395.2813
Fax: +1.240.395.2591
email: clee@path.org

PROTOCOL SIGNATURES

By my signature below, I agree to conduct the study according to this protocol version as approved by the IRB of record, in accordance with applicable IRB requirements, Good Clinical Practice and federal and local regulations. I will not modify the protocol without first obtaining an IRB approved amendment and new protocol version unless it is necessary to protect the health and welfare of study participants.

ANGELA TALLEY
Print Investigator Name

[signature]
Investigator Signature

29 MAR 2010
Date

Sponsor Approval signature:

Susan Lundeberg Clinical Quality Assurance Analyst
Sponsor Representative (Name/Title)

[signature]
Sponsor Representative Signature

29 Mar 2010
Date

PROTOCOL SYNOPSIS

**Protocol Title:**	**Reproducibility of Malaria Challenge in Healthy Volunteers**
**Study Phase:**	**N/A**
**Investigational Product:**	**Wild-type NF54 strain *Plasmodium falciparum* sporozoites**
**IND Number**	***pending***
**Study Objectives:**	**Primary Objective:** To demonstrate the safety, tolerability and infectivity of experimental *P. falciparum* challenge in healthy malaria-naïve adults<br>**Secondary Objective:** To qualify the immune response to experimental *P. falciparum* infection in healthy malaria-naïve adults.<br>**Exploratory Objective:** To evaluate the detection and quantification of sub-patent parasitemia by RT-PCR.
**Main Inclusion Criteria:**	a) Age 18 to 50 years<br>b) Gender: male or non-pregnant female<br>b) Good general health status as demonstrated by medical history, physical exam, and screening laboratory tests performed within 56 days of enrollment<br>c) Hemoglobin, WBC, platelets and creatinine within institutional normal range<br>d) Alanine aminotransferase (ALT), aspartate aminotransferase (AST), bilirubin and alkaline phosphatase < 1.25 times the institutional upper limit of normal<br>e) Normal urine as defined by negative urine glucose, negative or trace urine protein, and negative or trace urine hemoglobin (within institutional normal range)<br>f) Negative HIV-1 and 2 blood test<br>g) Negative Hepatitis B surface antigen (HBsAg)<br>h) Negative anti-Hepatitis C virus antibodies (anti-HCV)<br>i) Low risk for coronary heart disease (CHD) based on NHANES I cardiovascular risk assessment and screening electrocardiogram (ECG)<br>j) Ability and willingness to provide informed consent<br>k) Assessment of Understanding questionnaire completed prior to enrollment: demonstration of understanding for all questionnaire items answered incorrectly<br>l) Reliable access to the clinical trials center (CTC) and availability to participate for duration of study (approximately 4 months)<br>m) If the participant is biologically female she must:<br>▪ Have a negative serum or urine beta human chorionic gonadotropin (β-hCG) pregnancy test performed within 3 days prior to challenge<br>▪ Agree to consistently use effective contraception from 21 days prior to enrollment for the duration of the study, for sexual activity that could lead to pregnancy. Effective contraception is defined as using any 1 or more of the following methods:<br>1) Condoms (male or female) with or without a spermicide, or<br>2) Diaphragm or cervical cap with spermicide;<br>3) Intrauterine device (IUD),<br>4) Hormonal contraception, or<br>5) Successful vasectomy in the male partner<br>▪ OR not be of reproductive potential: i.e. be surgically, medically or physiologically sterile,<br>▪ OR be sexually abstinent<br>▪ Also agree to not seek pregnancy through alternative methods such as artificial insemination or in vitro fertilization until after the last scheduled protocol visit
**Main Exclusion Criteria:**	a) Planned travel to malaria endemic area during the study period<br>b) Recent travel to a malaria endemic area within 3 months of enrollment<br>c) Prior receipt of an investigational malaria vaccine<br>d) History of malaria diagnosis based on positive peripheral blood smear.<br>e) Use of malaria chemoprophylaxis with chloroquine within 5 months of malaria challenge, with

	atovaquone/proguanil within 2 months of malaria challenge or with mefloquine within 30 days prior to malaria challenge<br>f) Recent (within 30 days) or anticipated use of systemic antibiotics with anti-malarial effects (i.e., tetracyclines for dermatologic conditions, sulfa drugs for recurrent urinary tract infections, etc.) during the study period<br>g) Anticipated use of medications known to interact with chloroquine and/or atovaquone/proguanil during the study period<br>h) Use of any investigational or non-registered drug or vaccine within 30 days preceding challenge or planned use during the study period.<br>i) Immunosuppressive medications received within 6 months prior to challenge or planned use within 21 days after challenge (e.g., oral/parenteral corticosteroids, and/or cytotoxic medications). (Not excluded: [1] corticosteroid nasal spray for allergic rhinitis; and [2] topical corticosteroids for mild, uncomplicated dermatitis.)<br>j) Any vaccination received or planned within 30 days prior to challenge<br>k) Blood products or immunoglobulins received within 120 days of challenge<br>l) Screening laboratory abnormalities beyond the limits defined in Section 3.1.<br>m) Clinically significant medical condition, physical examination findings, other clinically significant abnormal laboratory results, or past medical history that may have clinically significant implications for current health status in the opinion of the Investigator. A clinically significant condition or process includes but is not limited to:<br>▪ A process that would affect the immune response,<br>▪ A process that would require medication that affects the immune response,<br>▪ Any contraindication to repeated phlebotomy,<br>▪ A condition or process in which signs or symptoms could be confused with reactions to malaria challenge and/or infection, including dermatologic abnormalities at the site of sporozoite inoculation, or<br>▪ Any condition specifically listed among the exclusion criteria below<br>n) History of anaphylaxis<br>o) History of severe allergic reactions to mosquito bites<br>p) History of, or known active cardiac disease including: (1) prior myocardial infarction (heart attack); (2) angina pectoris; (3) congestive heart failure; (4) valvular heart disease; (5) cardiomyopathy; (6) pericarditis; (7) stroke or transient ischemic attack; (8) exertional chest pain or shortness of breath; or (9) other heart conditions under the care of a doctor<br>q) Elevated (moderate or high) risk of coronary heart disease as determined by the NHANES I (22) cardiovascular risk assessment criteria (Appendix D)<br>r) Clinically significant ECG findings, as determined by the expert study cardiologist<br>s) Chronic or active neurologic disease (including seizures, migraine headaches, etc.)<br>t) History of splenectomy or functional asplenia<br>u) History of psoriasis or porphyria<br>v) History of ocular disease diagnosed by an ophthalmologist as retinopathy or visual field defects<br>w) Acute illness at the time of enrollment<br>x) Serologic positivity for HIV, hepatitis C and/or hepatitis B<br>y) Pregnant or lactating female or female who intends to become pregnant during the study period<br>z) Psychiatric condition that precludes compliance with the protocol including but not limited to:<br>▪ psychosis within the past 3 years,<br>▪ ongoing risk for suicide, or history of suicide attempt or gesture within the past 3 years<br>aa) Suspected or known current alcohol abuse as defined by the American Psychiatric Association in DSM IV.<br>bb) Recreational intravenous drug use in the last 12 months<br>cc) Any other finding that, in the judgment of the investigator, would interfere with, or serve as a contraindication to, protocol adherence, assessment of safety or reactogenicity, or a participant's ability to give informed consent or increase the risk of having an adverse outcome from participating in the study
**Study Design:**	**This is a prospective, single arm, single intervention safety and infectivity study in 6 healthy, malaria-naïve adults, conducted to demonstrate the successful implementation of**

	**the the malaria human challenge model at the Seattle Biomedical Research Institute (Seattle BioMed).**
**Study Schema:**	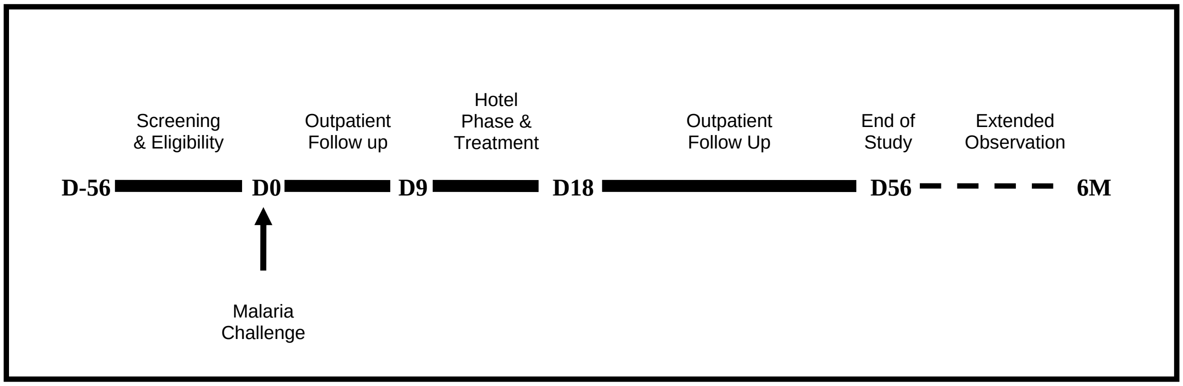
**Study Endpoints:**	**Primary endpoints:**<br>1. Local and systemic solicited adverse events (AE) assessed day 0 through 28 days post challenge<br>2. Occurrence of unsolicited AEs through 56 days post challenge<br>3. Development of malaria parasitemia and time to parasitemia demonstrated on peripheral blood smear<br>4. Occurrence of serious adverse events (SAEs) through 56 days post challenge<br>**Secondary endpoints**<br>1. Humoral and cell-mediated immune response to experimental malaria infection<br>**Exploratory endpoints**<br>1. Detection of sub-patent parasitemia and quantification of parasite densities by RT-PCR following malaria challenge<br>2. Time to detection of sub-patent parasitemia following malaria challenge by RT-PCR
**Study Procedures and Methods:**	**Challenge:** Study participants will undergo malaria sporozoite challenge with wild-type NF54 strain *P. falciparum* sporozoites administered via the bite of five infected *Anopheles stephensi* mosquitoes under controlled containment conditions.<br><br>**Follow-up Post- Challenge:** Participants will be closely monitored for acute reactogenicity and signs and/or symptoms of malaria infection, and from day five post-challenge, will have daily blood films examined for the presence of malaria parasites. Participants who develop malaria infection will be treated with a standard oral regimen of chloroquine, or other FDA-approved anti-malarial drugs, under direct observation. Participants will be treated upon first evidence of microscopic parasitemia or at day 18 if they remain negative. Participants will be housed with study staff in a local hotel for close observation from day 9 post-challenge, until three consecutive blood smears are negative and all symptoms have resolved, then followed weekly for a total of 8 weeks.
**Immunologic Assessments:**	1. Cell-mediated immune responses to *P. falciparum* infection including INF-γ ELISpot on day 0, 5, day of first positive blood smear, day 35 and 56.<br>2. Humoral immune responses to *P. falciparum* infection by ELISA for liver and blood stage antigens including CSP, AMA-1, and MSP-1 measured on day 0, 1, 5, day of first positive blood smear, and day 35.<br><br>In an effort to qualify the duration of immune response to experimental infection, participants will be invited to return for 2 additional visits at 3 months and 6 months after challenge. This extension of the main study will be conducted under separate extension consent.
**Stopping Rules:**	Stopping rules for individual participants are not applicable based on study design.
**Study Duration:**	Approximately 4 months, with a 6 month follow up phone call to all enrolled participants and an optional 6 month extension for immunology assessments
**Study Site:**	Malaria Clinical Trials Center (MCTC) at Seattle Biomedical Research Institute

TABLE OF CONTENTS

PROTOCOL SIGNATURES 3
ABBREVIATIONS AND ACRONYMS 10
1.0 Introduction 13
1.1 Background and Rationale 13
1.2 Study Product/ Investigational Agent/Intervention 15
1.3 Summary of Preclinical and Clinical Data 15
1.4 Potential Risks and Benefits 19
2.0 Objectives and Endpoints 22
2.1 Objectives 22
2.1.1 Primary Objective 22
2.1.2 Secondary Objective(s) 23
2.1.3 Additional/exploratory Objective(s) 23
2.2 Overview of Study Design 23
2.3 Endpoints 24
2.3.1 Primary Endpoints 24
2.3.2 Secondary Endpoints 24
3.0 Participant Selection and Withdrawal 25
3.1 Inclusion Criteria 25
3.2 Exclusion Criteria 26
3.3 Participant Recruitment and Screening 28
3.4 Early Withdrawal and Termination of Participants 29
4.0 Investigational Product/Treatment//Intervention 30
4.1 Description and Formulation 30
4.2 Dosing Schedule 30
4.3 Treatment Assignment 31
4.4 Preparation and Administration 31
4.5 Storage and Handling 33
4.6 Accountability (Release, Dispensing and Return of Investigational Product) 33
4.6.1 Release and Dispensing 33
4.6.2 Return of Study Product 33
4.7 Adherence Assessment 34
4.8 Toxicity Management 34
5.0 Study Procedures and Assessments 34
5.1 Pre-enrollment procedures 36
5.1.1 Screening 36
5.1.2 Informed Consent 36
5.1.3 Clinical Evaluations: 38
5.1.4 Concomitant Medications 39
5.1.4.1 Prohibited Therapy 40
5.1.4.2 Allowed Therapy 41
5.1.5 Determination of Eligibility 41
5.2 Post-enrollment procedures 41
5.2.1 Enrollment and Challenge 41
5.2.2 Reactogenicity Assessment 42

5.2.3 Malaria Assessment and Treatment 43
5.2.4 Toxicity Management 45
5.2.5 Follow up 45
5.3 Visit-by-Visit 46
5.3.1 Interim Contacts and Visits 46
5.4 Study Extension 46
5.5 Biological Laboratory Samples 47
5.5.1 Clinical Laboratory Evaluations 47
5.5.2 Immunologic Assays 48
5.5.3 Special Assays or Procedures 49
5.5.4 Specimen Handling and Storage 50
5.5.5 Future Use of stored Specimens 51
5.5.6 State Reporting Requirements 52

6.0 Safety and Adverse Events 52
6.1 Definitions 52
6.1.1 Adverse Event (AE) 52
6.1.2 Solicited Adverse Event 53
6.1.3 Unsolicited Adverse Event 54
6.1.4 Serious Adverse Event (SAE) 54
6.1.5 Unexpected Adverse Event 55
6.1.6 Unanticipated Problems 55
6.2 Classification 57
6.2.1 Severity/Intensity: 57
6.2.2 Relationship to Investigational Product 58
6.2.3 Outcome 59
6.3 Identification and Documentation of Adverse Events 59
6.4 Adverse Event Reporting 60
6.4.1 Serious Adverse Events 61
6.4.2 Other Immediately Reportable Events 62
6.4.3 Unanticipated Problems 62
6.4.4 Reporting to Regulatory Agencies 62
6.4.5 Reporting to the Institutional Review Board 63
6.5 Treatment of Adverse Events 63
6.6 Adverse Event Follow Up 64
6.7 Unblinding Procedures 64
6.8 Stopping Rules 64
6.8.1 Participant Discontinuation 64
6.8.2 Study Discontinuation 64
6.9 Monitoring 65
6.9.1 Medical and Safety Monitoring 65
6.9.2 Study Monitoring 65

7.0 Data Handling and Record Management 66
7.1 Data Management Responsibilities 66
7.2 Data Collection Process 66
7.2.1 Types of Data 66
7.2.2 Data Capture Methods 66
7.3 Source Document Requirements 67
7.3.1 Access to Source Documents 67
7.4 Required Records 68
7.4.1 Essential documents 68

7.4.2 Other ... 68
7.5 Record Retention ... 69
7.6 Protocol Deviations ... 69

8.0 Statistical Considerations ... 69
8.1 Sample Size Calculation ... 69
8.2 Analytical Plans ... 70
8.2.1 Analysis Population ... 70
8.2.2 Infectivity Analysis ... 70
8.2.3 Safety analysis ... 70
8.2.4 Immunogenicity Analysis ... 70

9.0 Quality Control and Quality Assurance ... 71

10.0 Ethics and Responsibility ... 72
10.1 Investigator Responsibility ... 72
10.2 Compensation ... 73
10.3 Exclusion of Special Populations ... 73
10.4 Institutional Review Board ... 73
10.4.1 Protocol Modifications ... 74
10.4.2 Reporting ... 74
10.5 Participant Confidentiality and Privacy ... 74
10.6 Research Related Injury ... 75

11.0 Publication and/or Presentation Policy ... 75

REFERENCES ... 76

Appendix A: Study Visit Procedures ... 79

Appendix B: Laboratory Procedures ... 80

Appendix C: Adverse Event Toxicity Grading Scales for Clinical and Laboratory Abnormalities ... 81

Appendix D: Cardiovascular Risk Assessment ... 85

Appendix E: Volunteer Compensation Schedule ... 88

ABBREVIATIONS AND ACRONYMS

AE	adverse event
ALT	alanine aminotransferase
ANCOVA	analysis of co-variance
ANOVA	analysis of variance
Ag	Antigen
AMA	apical membrane antigen
AUC	area under the curve
AR	army regulations
AST	aspartate aminotransferase
β-hCG	beta human chorionic gonadotropin
BB-MF	Biologics Master File
BS	blood smear
BMI	body mass index
BSE	bovine spongiform encephalitis
BUN	blood urea nitrogen
CRF	case report form
CRT	case report tabulations
CMI	cell mediated immunity
°C	degrees Celsius
CBER	Center for Biologics Evaluation and Research
CDC	Center for Disease Control and Prevention
CDER	Center for Drug Evaluation and Research
CeMPMIR	Center for Mosquito Production and Malaria in Research
cm	Centimeter
CMC	chemistry, manufacturing and control
CSP	circumsporozoite protein
CDM	clinical data management
CLIA	Clinical Laboratory Improvement Amendments
CPM	Clinical Project Manager
CRC	Clinical Research Center
CRO	clinical research organization
CSR	clinical study report
CTMF	Clinical Trial Master File
CTC	Clinical Trials Center
CS	clinically significant
CFR	Code of Federal Regulations
CAP	College of American Pathologists
CBC	complete blood count
CI	confidence interval
CHD	coronary heart disease
CIOMS	Council for International Organization of Medical Science
Cr	Creatinine
CV	Curriculum Vitae
DB	data base
DMC	Data Monitoring Committee
dL	deciliter
DNA	deoxyribonucleic acid
DoD	Department of Defense

DA	Department of the Army
DSM	Diagnostic and Statistical Manual of Mental Disorders vol IV
ECG	Electrocardiogram
EDC	electronic data capture
EHS	Environmental Health and Safety
ELISA	enzyme linked immunosorbant assay
ELISpot	enzyme linked immunospot assay
EC	ethics committee
EDTA	ethylenediaminetetraacetic acid
°F	degrees Fahrenheit
GAP	genetically attenuated parasite
GCP	Good Clinical Practice
GLP	Good Laboratory Practice
GMP	Good Manufacturing Practice
GBS	Guillain-Barré Syndrome
HIPAA	Health Insurance Portability Authorization Act
Hb	Hemoglobin
HBsAg	hepatitis B surface antigen
HCV	hepatitis C virus
hpf	high power fields
HIV	human immunodeficiency virus
Ig	Immunoglobulin
IEC	Independent Ethics Committee
IT	information technology
IC	informed consent
ITHS	Institute of Translational Health Sciences
IRB	Institutional Review Board
IFNγ	interferon gamma
IL	Interleukin
ICH	International Conference on Harmonization
ICS	intracellular cytokine staining
IND	Investigational New Drug Application
IB	investigator's brochure
kg	Kilogram
LS	liver stage
LSA	liver stage antigen
LDL	low density lipoprotein
LP	lymphocyte proliferation
MCTC	Malaria Clinical Trials Center
MCB	master cell bank
MVI	PATH Malaria Vaccine Initiative
MedDRA	Medical Dictionary for Regulatory Activities
MSP	merozoite surface protein
μl	microliter
mg	Milligram
ml	Milliliter
mm	Millimeter
NHANES	National Health And Nutritional Examinations Survey
NIH	National Institutes of Health
NMRC	Naval Medical Research Center
NDA	New Drug Application

NCS	not clinically significant
NTF	note to file
OSHA	Occupational Safety and Health Administration
OHRP	Office of Human Research Protections
OTSG	Office of the Surgeon General
PTID	participant identification number
PBMC	peripheral blood mononuclear cell
PD	pharmacodynamics
PK	pharmacokinetics
Pf	*Plasmodium falciparum*
PCR	polymerase chain reaction
PDF	portable document format
PI	Principal Investigator
PATH	Program for Appropriate Technology in Health
QA	quality assurance
QAU	quality assurance unit
QC	quality control
RT-PCR	real time-polymerase chain reaction
RA	regulatory affairs
SMC	Safety Monitoring Committee
Seattle BioMed	Seattle Biomedical Research Institute
SAE	serious adverse event
SD	source document
spz	sporozoite
SOP	standard operating procedure
SAP	statistical analysis plan
SAS	Statistical Analysis Software
TSC	Trial Steering Committee
TNFα	tumor necrosis factor alpha
DHHS	U.S. Department of Health and Human Services
FDA	U.S. Food and Drug Administration
USAMMDA	United States Army Medical Materiel Development Activity
USAMRMC	United States Army Medical Research and Materiel Command
USP	United States Pharmacopeia
VS	vital signs
WRAIR	Walter Reed Army Institute of Research
DOH	Washington State Department of Health
WBC	white blood cell
WCB	working cell bank
WHO	World Health Organization

1.0 Introduction

1.1 Background and Rationale

1.1.1 Burden of Disease

Malaria is the most prevalent and deadly human parasitic disease, afflicting 300-500 million people annually resulting in nearly a million deaths each year; most are children under the age of five and pregnant women(1, 2). Malaria related morbidity and mortality has a major economic impact in endemic regions, predominantly resource poor countries in Sub-Saharan Africa, Southeast Asia and South America. Malaria likewise presents a significant health risk to non-immune travelers to endemic regions and military personnel deployed overseas.(3) While medications exist for treatment and prophylaxis of malaria infection, their efficacy is limited by issues of accessibility, toxicity, prohibitive cost, poor compliance and increasing parasite resistance to available drugs. Vector control efforts have helped to reduce transmission and limit the burden of disease, although coverage is incomplete. To stem the worldwide impact of this devastating disease, a safe and effective malaria vaccine and improved anti-malarial therapeutics are urgently required.

1.1.2 Malaria Life Cycle and Vaccine Strategies

Malaria is a vector-borne illness caused by infection with species of the apicomplexan parasite, *Plasmodium*. Of the five major species commonly known to infect humans (*P. vivax, P. ovale, P. malariae, P. knowlesi and P. falciparum*), the majority of deaths are caused by *P. falciparum*. The complex parasite life cycle includes developmental stages in obligate mammalian and insect hosts, presenting both challenges and opportunities for development of malaria vaccines and novel drugs. The parasite is transmitted to humans by the bite of the female *Anopheline* mosquito by inoculating a relatively small number of sporozoites into the host during a blood meal. The haploid sporozoites travel in the bloodstream to the liver, invade hepatocytes and undergo intracellular replication. This clinically silent, exo- or pre-erythrocytic stage is the target of leading vaccine strategies utilizing conserved antigenic targets to elicit protection against sporozoite migration and hepatocyte invasion and development. A vaccine at this stage would ideally provide sterile immunity, preventing progression to blood stage infection and clinical disease. After five to seven days hepatocytes rupture releasing thousands of merozoites into the

bloodstream, initiating the pathogenic erythrocytic stage. The merozoites invade erythrocytes and undergo asexual replication and maturation, then rupture the erythrocytes, releasing additional merozoites which can reinitiate the cycle of erythrocyte invasion, replication and release. This continuous cycle is responsible for the clinical symptoms of the disease including fever, chills, malaise, myalgia, arthralgia, nausea and vomiting, and in the case of *P. falciparum* can result in severe illness including cerebral malaria, pulmonary edema, renal failure, shock and death. Vaccine development goals at this stage are to limit the severity of disease and resultant morbidity and mortality by decreasing overall parasite burden. A small number of blood stage parasites ultimately differentiate into male and female gametocytes and enter the sexual phase of the life cycle inside the mosquito midgut when taken up in a blood meal. Sexual stage parasites progress through several developmental forms through the mosquito midgut until ultimately, infective asexual sporozoites reach the salivary glands to complete the life cycle.

1.1.3 Rationale for the Current Study

In the absence of defined immune correlates of protection and reliably predictive animal models, human challenge studies of malaria have been the most effective means of assessing early stage efficacy of candidate vaccines and anti-malarial therapeutics to prioritize those best suited for further development and clinical field trials in endemic populations. Under this model, human volunteers are inoculated with *P. falciparum* sporozoites by the bite of infected mosquitoes under controlled conditions, monitored closely for development of patent parasitemia and treated with standard doses of anti-malarial medications defined by the known sensitivity profile of the parasite.(4, 5) The existing worldwide infrastructure for conducting these unique studies is currently inadequate to meet the projected global need based on the current pipeline of malaria therapeutics under development.(6) Currently, dedicated facilities for malaria challenge studies are limited to three centers: one in the Netherlands, one in the U.K. and the only U.S. facility, located at the Walter Reed Army Institute of Research (WRAIR). The Seattle Biomedical Research Institute (Seattle BioMed) collaborates with WRAIR scientists in the effort to develop products for the US military. WRAIR has been safely conducting human challenge studies since the mid 1980s, however there is mutual recognition that expanded infrastructure is urgently needed and have partnered with Seattle BioMed to establish the human challenge model at the Malaria Clinical Trials Center (MCTC) at

Seattle BioMed. The recently developed Malaria Clinical Trials Center is an innovative center for the integration of basic science and clinical research, with an immediate mandate to test and license an effective malaria vaccine. In order to demonstrate our ability to safely transfer the WRAIR challenge model to Seattle BioMed, this study will conduct a controlled demonstration challenge trial in six healthy volunteers.

By establishing the human challenge model at Seattle BioMed, the MCTC will increase capacity and accelerate safety and efficacy assessment of:

- Genetically attenuated parasite vaccines that prevent infection with *P. falciparum*
- Subunit pre-erythrocytic vaccines that prevent infection with *P. falciparum*
- Subunit vaccines that limit blood stage parasitemia with *P. falciparum*
- Drugs that prevent infection with *P. falciparum*

Seattle BioMed scientists are recognized leaders in the field of malaria vaccinology and immunology. Seattle BioMed is also home to the Center for Mosquito Production and Malaria Infection Research (CeMPMIR), a state-of-the-art entomology facility for the production and study of malaria sporozoites and infected mosquitoes. Thus, as an integrated center for translational research, the MCTC will catalyze basic and applied research in malaria immunology and vaccinology. As the central component, the human challenge model will accelerate the pace of testing to enhance the qualification of selected candidate immunogens, and will ensure that optimized candidates rapidly advance along the development pipeline.

1.2 Study Product/ Investigational Agent/Intervention

Malaria sporozoite challenge with the wild-type NF54 strain of *Plasmodium falciparum* will be delivered to human volunteers by the bite of infected *Anopheles stephensi* mosquitoes. The *P. falciparum* parasites are provided by the Walter Reed Army Institute of Research from its Master Cell Bank (MCB) and produced as described in WRAIR BB-MF #5855 and as per WRAIR Division of Entomology procedures using the NF54 strain of *Plasmodium falciparum*. The mosquito feedings will be performed in the secure insectary of the Center for Mosquito Production and Malaria Infection Research (CeMPMIR) at Seattle BioMed.

1.3 Summary of Preclinical and Clinical Data

While preclinical data from murine and primate malaria challenge models is useful to inform early vaccine candidate selection, the response to vaccination and challenge in these models may not predictably translate to human malaria interventions. Validation of safety and immunogenicity in small phase one studies reduces development costs and risks to volunteers by ensuring that only the most viable candidates advance to large scale testing in endemic areas. As such, human challenge models of malaria have been the mainstay of early phase vaccine and drug candidate evaluation for decades.(5, 7, 8)

Studies of experimental malaria infection in humans conducted over the last century are responsible for much of the current knowledge on malaria transmission, pathogenesis, immunity and vaccine and drug efficacy.(7, 9, 10, 11) Malaria challenge of human volunteers was accepted in the setting of the widespread use of "malariatherapy" for treatment of neurosyphilis prior to the availability of penicillin. Subsequent studies utilizing experimental challenge for evaluation of drugs were critical to the development of many currently available antimalarials. These early challenge interventions employed both vector and intravenous inoculations of malaria parasites usually obtained from infected volunteers whose infections were often allowed to progress until clinical symptoms mandated treatment.(5, 12)

The challenge model has been used for evaluation of malaria vaccine efficacy starting in the 1970s with irradiated sporozoite vaccination and challenge trials with *P. falciparum* and *P. vivax*.(13-16) This approach was successful, albeit difficult to deliver on a large scale due to the logistics of the model, which required administration of >1000 infectious bites. Insight gained from evaluation of immune responses to vaccination in these studies allowed identification of several candidate immunogens included in later vaccine platforms.(16) Importantly, these studies demonstrated the safety and tolerability of malaria challenge for evaluating vaccine efficacy.

The challenge model was optimized in the mid 1980s with the standardization of parasite culture and mosquito infection by membrane feeding, thereby decreasing the risks to challenge participants considerably. Chulay et al. (4) first described infection of six healthy volunteers with the NF54 strain of *P. falciparum* administered under controlled conditions by the bite of infected mosquitoes produced *in vitro.* All participants developed patent (microscopic) parasitemia within 7-11 days and were treated immediately upon diagnosis with chloroquine, often

prior to the onset of clinical symptoms. All six participants ultimately developed symptoms of malaria for 2-3 days and recovered post treatment. The improved challenge model using membrane-fed mosquitoes was safely and reliably adopted thereafter for the assessment of malaria vaccine and drug candidates. Three comprehensive reviews of challenge trial participants conducted from 1985-2002 have defined the clinical course following infection, response to treatment and time course of laboratory abnormalities in challenge participants.(8, 17, 18) These reviews have consistently demonstrated the safety and reliability of the challenge model conducted in this manner.

A review of 118 vaccine trial participants in 18 different trials conducted from 1985-1992, including 56 infectivity controls, showed overall that the model was safe, reliable and generally well tolerated.(17) The majority of participants were symptomatic, with a mean duration of symptoms of 3 days. The predominant symptoms were fever, chills, myalgia, arthralgia and fatigue and frequency of symptoms notably increased after blood smear diagnosis and initiation of treatment. The mean prepatent period was 11.5 days, and 36% percent were asymptomatic at the time of blood smear diagnosis. Reductions in platelet and white blood cell (WBC) counts were the predominant laboratory abnormalities, reaching a nadir on day two post-treatment with subsequent recovery to baseline by day 21. Significant aminotransferase elevations occurred in two participants and likewise resolved without sequelae. Serum electrolytes, BUN, and creatinine remained normal in all volunteers. Likewise, symptoms were similar to those seen in naturally infected non-immune individuals but the clinical course shorter and milder due to early diagnosis and treatment under controlled conditions.

In a study by Verhage et al.(18) to evaluate infectivity and clinical safety of different protocols for experimental malaria challenge, 30 non-immune volunteers were challenged with the NF54 strain of *P. falciparum* by the bites of 4-7 or 1-2 mosquitoes. The latter group had a 50% infectivity rate, whereas all participants receiving higher number of infectious bites developed patent infection, with the mean prepatent period of 8.8 days. All volunteers who developed patent parasitemia had a mild, uncomplicated episode of clinical malaria. The most commonly reported symptoms were headache, malaise and/or fatigue, myalgia, and arthralgia. The most common laboratory abnormalities were transient thrombocytopenia and decreased WBC counts, which resolved to baseline by day 10 post-treatment. All volunteers were treated with chloroquine

for diagnosed malaria or at the end of the study if they remained negative. The majority were treated upon detection of parasitemia on blood smear, but treatment was delayed by 24-48 hours in a subset of volunteers to monitor parasite replication and immune response. Symptoms and lab abnormalities were similar in this group with the exception of higher fevers at greater frequency. One volunteer who remained negative for malaria developed a myocardial infarction while on study and recovered. This volunteer was retrospectively assessed to have moderate risk for a coronary event within ten years. The authors comment that despite the general knowledge that cardiac complications are extremely rare during and after adequate treatment for malaria, they planned to exclude volunteers with a coronary event greater than 10% in future challenge studies.

A review by Epstein et al. (8) confirmed the safety and reliability of experimental challenge based on experience from 47 trial participants challenged with the NF54 strain of *P. falciparum*. The majority of records were from a single trial conducted at the Navy Medical Research Center (NRMC) between 1998 and 2002 and included 16 infectivity controls. The mean prepatent period was 10.52 days with a range from 9-14 days. All volunteers were symptomatic with a mean incubation period of approximately 9 days, indicating that the majority of participants experienced at least 1 symptom prior to microscopic diagnosis. The predominant signs/symptoms were mild to moderate fever, fatigue, headache, malaise, chills and myalgia lasting on average 1-3 days. Twenty one percent had severe symptoms by toxicity grading scales although none met the accepted clinical criteria for severe malaria and there were no hospitalizations. Laboratory abnormalities at day 10-12 post-challenge mirrored those of prior studies including WBC and platelet count reductions, and aminotransferase elevations. The majority of lab abnormalities attributable to infection and challenge were mild-moderate and resolved by week four post-challenge. A small number of grade 3 abnormalities were noted but relationship to infection and challenge were not definitively determined. Based on their review, the authors note that the clinical course following challenge is fairly predictable with most participants becoming mildly to moderately ill for several days but retaining ability to perform most of their routine activities. Further they note that laboratory assessment is unlikely to directly affect clinical management but may be helpful in documenting the clinical course. Thus controlled experimental challenge is safe, reproducible, and well tolerated with close monitoring, rapid diagnosis and treatment making it unlikely

that any participant would develop severe malaria and/or require hospitalization.

1.4 Potential Risks and Benefits

1.4.1 Risks from malaria challenge, infection and treatment

Risks associated with malaria challenge include local reactions to mosquito bites, including pain, discomfort, erythema, induration, edema, pruritis, and urticaria and possibly more extensive local reactions involving the arm including regional lymphadenitis. As with any investigational product administration and no matter what precautions are taken, there is always the risk of a serious, or even life-threatening, allergic reaction to mosquito bites or malaria infection. Participants with known adverse reactions to mosquito bites or history of anaphylaxis are excluded from participating in the study. Local reactions will be treated with topical and/or systemic medications as described in section 4.8. Participants will be closely monitored during the challenge and in the acute post-challenge period by the Investigator and clinical staff who are trained and equipped to respond immediately to systemic reactions including anaphylaxis, angioedema, bronchospasm, and laryngospasm.

Additional risks include side effects from anti-malarial medications including chloroquine, quinine, atovaquone/proguanil or doxycycline (e.g., nausea, vomiting, diarrhea, headaches, blurred vision, pruritus, tinnitus, or photosensitivity). The close monitoring of participants in the study allows for early diagnosis and treatment, thus complications of malaria frequently seen in naturally acquired infections at higher levels of parasitemia (e.g., anemia, multi-organ failure, coma and death), are unlikely. Under the carefully controlled conditions of this study, the chance of serious illness or death from malaria infection is very low.

Transient clinical symptoms and laboratory abnormalities, such as fever, headache, mild anemia, leukopenia, thrombocytopenia, splenomegaly, abdominal pain and fatigue, are expected findings in uncomplicated clinical malaria. If a participant becomes ill or injured as a direct result of participating in this research study, (s)he will be provided medical care for that illness or injury at no cost to the participant. In challenge studies involving over 900 study subjects over the past 23 years at the WRAIR, there has never been a case of mortality or excess morbidity requiring inpatient hospitalization (WRAIR, unpublished communication). WRAIR staff with experience in the conduct of malaria human challenge trials will

be available in an advisory capacity throughout the preparation and conduct of the trial.

Recent concern for potential risk of cardiac abnormalities in the setting of malaria challenge trials was raised in relation to an isolated event in a participant in a malaria vaccine challenge trial in the Netherlands (27). A 20 year old participant reported chest pain and was found to have elevated cardiac enzymes after receiving an investigational malaria vaccine, malaria challenge and treatment with a non FDA-approved antimalarial drug. The participant recovered uneventfully without sequelae and the cause of the cardiac event, thought to be either an acute coronary syndrome or myocarditis, was not established. There is insufficient data to determine with any certainty a relationship to the study product, malaria challenge, infection or treatment, as multiple factors possibly contributing to the cardiac event were identified, however the authors note that the temporal relationship and the apparent absence of an underlying condition make the infection the most probable trigger. Primary cardiac abnormalities in uncomplicated clinical malaria are extremely rare (19) and the need for routine cardiac monitoring of patients with severe malaria is questionable, even in patients receiving treatment with antimalarials with known effects on cardiac electrical conduction.(20, 21) However, to minimize potential risks for cardiac abnormalities, all volunteers will undergo a cardiac risk assessment as described in section 5.1.3 and only volunteers classified as low risk (<10% risk for coronary event in the next 5 years) will be enrolled in the study. Participants will be questioned about cardiac symptoms while on study and will undergo further cardiac evaluation as needed should these symptoms arise.

The risk of transmission of other infectious agents by mosquitoes is extremely low. No documented cases of HIV, syphilis or viral hepatitis transmission from *Anopheles* mosquitoes to humans have been reported. Likewise, it would be unlikely for other infectious pathogens to survive in the invertebrate host for the duration of the time between mosquito infection and challenge (approximately 16 days). Blood used for mosquito blood meal and infection has been provided by an FDA licensed facility and commercially tested using FDA-approved test methods for HIV-1 and HCV-RNA, syphilis and hepatitis B surface antigen; all blood has tested negatively for these pathogens.

Malaria infection during pregnancy can have adverse effects on both mother and fetus, including maternal anemia, fetal loss, premature delivery, intrauterine growth retardation, and delivery of low birth-weight

infants. As such, women who are pregnant or plan to become pregnant during the study period are excluded from the study. Pregnancy testing is performed prior to enrollment. Pregnancy prevention counseling is performed during screening and reinforced while on study. Because the drugs used for treating malaria in the study may not be completely safe during lactation, women who are breastfeeding are also excluded from participation.

1.2.1 Risk from phlebotomy

Phlebotomy carries a minimal risk of minor discomfort and the possibility of bruising at the site of the needle puncture and, rarely, the possibility of infection and/or clot formation at the needle puncture site. Screening diagnostic assays may reveal a new diagnosis or disease process previously unknown to the participant, which could cause psychological stress to the participant. In such a case, the participant will be provided with counseling and referrals to appropriate medical care if necessary.

1.2.2 Confidentiality

While every precaution will be made to ensure participant confidentiality, there is a risk associated with possible breach of confidentiality and release of participant's protected health information. All study-related information will be stored securely at the study site. All volunteer information will be stored in locked file cabinets in areas with access limited to study staff. Clinical laboratory specimens processed at the clinical laboratory and subsequent results may contain participant identifying information, but will be coded for data transcription and reporting and original reports stored in the participant's study binder. All other laboratory specimens, reports, study data collection, process and administrative forms will be identified by coded number to maintain participant confidentiality. Computer entry will be done by coded number, and all local databases will be secured with password-protected access systems. Forms, lists, logbooks, appointment books and any other listings that link volunteer ID numbers to other identifying information will be stored in a locked file in an area with limited access. Participant's study information will not be released without the written permission of the participant, except as necessary for monitoring by the Sponsor, Institutional Review Board (IRB) or regulatory authorities or as required by law.

1.2.3 Risk to study staff

Risks to staff include exposure to blood borne pathogens through handling of needles contaminated by blood or body fluids or other biohazardous waste generated from phlebotomy. Universal precautions will be followed at all times during collection, handling and processing of biologic specimens. Only staff with adequate training will perform phlebotomy. Handling and disposal of biohazardous materials will observe Seattle BioMed environmental health and safety (EHS) policies and Occupational Health and Safety Administration (OSHA) regulations. Staff members present in the insectary during the malaria challenge have a small risk of exposure to malaria infection. However these risks are mitigated by strict CeMPMIR procedures regarding handling of infected mosquitoes and conduct of the challenge.

1.2.4 Risk to the community

The risk of malaria transmission to any individual in the community is considered negligible. The infected mosquitoes are reared under eight levels of containment within the secure insectary and challenges are conducted in the challenge room within the secure facility, thus the likelihood of an infected mosquito escaping is low. Malaria is not contagious and cannot be spread by person-to-person contact. Further, participants are routinely diagnosed and treated before the infective form of the parasite (gametocyte) appears in the blood.

1.2.5 Other risks

There may be unknown risks that cannot be determined at this time, based on current information about the investigational product.

1.2.6 Benefits of participation

Participation in the study is voluntary. There is no direct benefit to the volunteer for participating in the study, however, the information gained from this study and the future conduct of malaria challenge studies under this model may contribute to the development of a safe and effective malaria vaccine, improved therapeutics and better understanding of malaria pathogenesis and immunity.

2.0 Objectives and Endpoints

2.1 Objectives

2.1.1 Primary Objective

To demonstrate the safety, tolerability and infectivity of experimental *P. falciparum* sporozoite challenge in healthy malaria-naïve adults.

2.1.2 Secondary Objective

To qualify the immune response to experimental *P. falciparum* infection in healthy malaria-naïve adults.

2.1.3 Additional/exploratory Objective

To evaluate detection and quantification of sub-patent parasitemia by RT-PCR.

2.2 Overview of Study Design

This is a prospective, single arm, single intervention, safety and infectivity study in 6 healthy, malaria-naïve adults, conducted to demonstrate the successful transfer of the WRAIR malaria challenge model to Seattle BioMed. Study participants will undergo malaria sporozoite challenge with wild-type NF54 strain *P. falciparum* sporozoites administered via the bite of five infected *Anopheles stephensi* mosquitoes under controlled containment conditions. Participants will be closely monitored for acute reactogenicity and signs and/or symptoms of malaria infection, and from day five post-challenge, will have daily blood films examined for the presence of malaria parasites. Participants who develop malaria infection will be treated with a standard oral regimen of chloroquine, or other FDA-approved anti-malarial drugs, under direct observation. Participants will be treated upon first evidence of microscopic parasitemia or at day 18 if they remain negative. Participants will be housed with study staff in a local hotel for close observation from day 9 post-challenge until three consecutive blood smears are negative post-treatment and all symptoms have resolved, then followed weekly for a total of 8 weeks. Periodic laboratory monitoring will be performed for safety assessment and to evaluate the immune response to malaria infection. Data collection will be done at the site. WRAIR staff experienced in conduct of human challenge studies for malaria will be available in an advisory capacity throughout the preparation and conduct of the trial.

In an effort to qualify the duration of immune response to experimental infection, participants will be invited to return for 2 additional visits at 3 months and 6 months after challenge. This extension of the main study will be conducted as described in Section 5.4 under a separate extension consent for participation.

Study Schema:

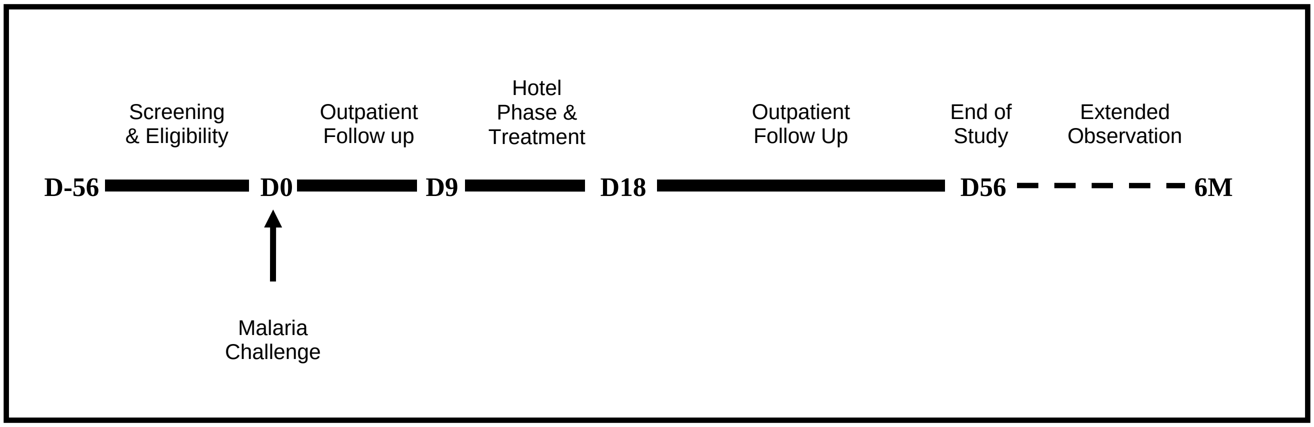

2.3 Endpoints

2.3.1 Primary Endpoints

1. Solicited adverse events (AEs) assessed day 0 through 28 days post-challenge.
2. Occurrence of unsolicited AEs through 56 days post-challenge
3. Development of malaria parasitemia and time to parasitemia as demonstrated on blood smear from day five through day 28 post-challenge
4. Occurrence of serious adverse events (SAEs) through 56 days post-challenge

2.3.2 Secondary Endpoints

1. Cell-mediated immune responses to *P. falciparum* infection including INF-γ ELISpot on day 0, 5, day of first positive blood smear, day 35 and 56.
2. Humoral immune responses to *P. falciparum* infection by ELISA for liver and blood stage antigens including CSP, AMA-1, and MSP-1 measured on day 0, 1, 5, day of first positive blood smear, and day 35.

2.3.3 Exploratory Endpoints

1. Detection of sub-patent parasitemia and quantification of parasite densities by RT-PCR following malaria challenge.
2. Time to detection of sub-patent parasitemia following malaria challenge by RT-PCR

3.0 Participant Selection and Withdrawal

3.1 Inclusion Criteria

a) Age 18 to 50 years

b) Gender: male or non-pregnant female

c) Good general health status as demonstrated by medical history, physical exam, and screening laboratory tests performed within 56 days of enrollment

d) Hemoglobin, WBC, platelets and creatinine within institutional normal range

e) Alanine aminotransferase (ALT), aspartate aminotransferase (AST), bilirubin and alkaline phosphatase < 1.25 times the institutional upper limit of normal

f) Normal urine as defined by negative urine glucose, negative or trace urine protein, and negative or trace urine hemoglobin (within institutional normal range)

g) Negative HIV-1 and 2 blood test

h) Negative Hepatitis B surface antigen (HBsAg)

i) Negative anti-Hepatitis C virus antibodies (anti-HCV)

j) Low risk for coronary heart disease (CHD) based on NHANES I cardiovascular risk assessment and screening electrocardiogram (ECG)

k) Ability and willingness to provide informed consent

l) Assessment of Understanding questionnaire completed prior to enrollment: demonstration of understanding for all questionnaire items answered incorrectly

m) Reliable access to the clinical trials center (CTC) and availability to participate for duration of study (approximately 4 months)

n) If the participant is biologically female she must:

- Have a negative serum or urine beta human chorionic gonadotropin (β-hCG) pregnancy test performed within 3 days prior to challenge
- Agree to consistently use effective contraception from 21 days prior to enrollment for the duration of the study, for sexual activity

that could lead to pregnancy. Effective contraception is defined as using any 1 or more of the following methods:

1) Condoms (male or female) with or without a spermicide, or
2) Diaphragm or cervical cap with spermicide;
3) Intrauterine device (IUD),
4) Hormonal contraception, or
5) Successful vasectomy in the male partner

- OR not be of reproductive potential: i.e. be surgically, medically or physiologically sterile,
- OR be sexually abstinent
- Also agree to not seek pregnancy through alternative methods such as artificial insemination or in vitro fertilization until after the last scheduled protocol visit

3.2 Exclusion Criteria

a) Planned travel to malaria endemic area during the study period
b) Recent travel to a known malaria endemic region within 3 months of enrollment
c) Prior receipt of an investigational malaria vaccine
d) History of confirmed malaria diagnosis based on positive peripheral blood smear.
e) Use of malaria chemoprophylaxis with chloroquine within 5 months of malaria challenge, with atovaquone/proguanil within 2 months of malaria challenge or with mefloquine within 30 days prior to malaria challenge
f) Recent (within 30 days) or anticipated use of systemic antibiotics with anti-malarial effects (i.e., tetracyclines for dermatologic conditions, sulfa drugs for recurrent urinary tract infections, etc.) during the study period
g) Anticipated use of medications known to interact with chloroquine and/or atovaquone/proguanil during the study period
h) Use of any investigational or non-registered drug or vaccine within 30 days preceding challenge or planned use during the study period.

i) Immunosuppressive medications received within 6 months prior to challenge or planned use within 21 days after challenge (e.g., oral/parenteral corticosteroids, and/or cytotoxic medications). (Not excluded: [1] corticosteroid nasal spray for allergic rhinitis; and [2] topical corticosteroids for mild, uncomplicated dermatitis.)

j) Any vaccination received or planned within 30 days prior to challenge

k) Blood products or immunoglobulins received within 120 days of challenge

l) Screening laboratory abnormalities beyond the limits defined in Section 3.1.

m) Clinically significant medical condition, physical examination findings, other clinically significant abnormal laboratory results, or past medical history that may have clinically significant implications for current health status in the opinion of the Investigator. A clinically significant condition or process includes but is not limited to:

- A process that would affect the immune response,
- A process that would require medication that affects the immune response,
- Any contraindication to repeated phlebotomy,
- A condition or process in which signs or symptoms could be confused with reactions to malaria challenge and/or infection, including dermatologic abnormalities at the site of sporozoite inoculation, or
- Any condition specifically listed among the exclusion criteria below

n) History of systemic anaphylaxis

o) History of severe allergic reactions to mosquito bites

p) History of, or known active cardiac disease including: (1) prior myocardial infarction (heart attack); (2) angina pectoris; (3) congestive heart failure; (4) valvular heart disease; (5) cardiomyopathy; (6) pericarditis; (7) stroke or transient ischemic attack; (8) exertional chest pain or shortness of breath; or (9) other heart conditions under the care of a doctor

q) Elevated (moderate or high) risk of coronary heart disease as determined by the NHANES I (22) cardiovascular risk assessment criteria (Appendix D)

r) Clinically significant ECG findings, as determined by the expert study cardiologist

s) Chronic or active neurologic disease (including seizures, migraine headaches, etc.)

t) History of splenectomy or functional asplenia

u) History of psoriasis or porphyria

v) History of ocular disease diagnosed by an ophthalmologist as retinopathy or visual field defects

w) Acute illness at the time of enrollment

x) Serologic positivity for HIV, hepatitis C and/or hepatitis B

y) Pregnant or lactating female or female who intends to become pregnant during the study period

z) Psychiatric condition that precludes compliance with the protocol including but not limited to:

- Psychosis within the past 3 years,
- ongoing risk for suicide, or history of suicide attempt or gesture within the past 3 years

aa) Suspected or known current alcohol abuse as defined by the American Psychiatric Association in DSM IV.

bb) Recreational intravenous drug use in the last 12 months

cc) Any other finding that, in the judgment of the investigator, would interfere with, or serve as a contraindication to, protocol adherence, assessment of safety or reactogenicity, or a participant’s ability to give informed consent or increase the risk of having an adverse outcome from participating in the study

dd) Clinical Trial Staff with direct involvement in the conduct of the trial will not be allowed to participate; not excluded: Seattle Biomed staff with no direct role in the study.

3.3 Participant Recruitment and Screening

A total of 6 participants meeting eligibility criteria will be enrolled in the study. Participants will be recruited through a variety of IRB approved outreach methods and messaging, from the greater Seattle and surrounding area, within a reasonable distance to ensure reliable access to the CTC and compliance with visit follow-up. Examples of recruitment outreach methods include but are not limited to: flyers, postcards, email, posting on public access listserves, newspaper advertising, etc. and will contain previously approved IRB messaging with information about the study. With their consent, participants at any stage of the screening process will

be entered into a screening database at the CTC for tracking progress through the process, recruitment statistics and for possible contact for participation in future studies.

Screening activities as outlined in section 5.0 will take place at the CTC at Seattle BioMed. Prior malaria challenge trial experience at WRAIR estimates a 4:1 ratio of screened to eligible participants, thus the CTC will anticipate screening at least 24-32 volunteers for eligibility to participate to ensure the availability of 6 eligible participants and 2 back up eligible potential participants.

The screening process is outlined in detail in section 5.1 and includes the informed consent process and laboratory and clinical assessments of eligibility. Some recruitment efforts may be employed prior to the participant consenting, including the use of a telephone script to prescreen potential participants contacting the site before they come to the clinic for a full screening visit. However, participants must sign a protocol-specific consent before any procedures to determine eligibility are performed. All recruitment and prescreening materials will be submitted to the IRBs/IECs for human subjects review and approval prior to use in the screening process.

3.4 Early Withdrawal and Termination of Participants

Since this is a single intervention protocol with malaria challenge conducted on the day of enrollment, it is not anticipated that participants will be held or discontinued from further intervention. However, a participant may voluntarily withdraw at any time.

Under certain circumstances, an enrolled participant may be terminated from further participation in the study. Specific events that may lead to termination from the study include:

1) Participant withdraws consent, refuses further participation or fails to comply with study procedures;
2) Participant relocates to an area away from the CTC and remote follow-up is not possible;
3) The investigator determines that the participant is lost to follow-up;
4) Investigator or participant’s primary care physician decides that the participant should be withdrawn from the study without his/her consent due to concerns that continued participation may present a risk to the health/safety of the participant, fellow participants or

study staff including behavioral issues identified during the study (e.g. illicit drug use or inappropriate behavior toward clinic staff).

5) The Sponsor decides that the participant should be withdrawn or the Sponsor stops the study for any reason, (e.g., receipt of prohibited therapy that may impact participant safety or assessment of study endpoints).

In any of the above categories, participants will be administered immediate treatment with chloroquine and, where possible, continue to be followed for safety evaluations. Counseling will be provided about the participant's health if he or she decides to discontinue participation in the trial. Medical advice in the best interest of the participant will be provided. Every reasonable effort will be made to locate participants who have relocated or have been lost to follow up.

When a participant withdraws due to an adverse event or is withdrawn by the Investigator due to an adverse event, the Sponsor Medical Monitor must be notified within 24 hours as outlined in Section 6.4.2. Participant withdrawal will not affect provision of medical evaluation and treatment for research related injury or illness as described in Section 10.6.

Participants who are withdrawn will not be replaced.

4.0 Investigational Product/Treatment//Intervention

The study product/intervention is described in further detail in the Investigator's Brochure (IB).

4.1 Description and Formulation

The Investigational Product (challenge material) is comprised of wild-type NF54 strain *Plasmodium falciparum* sporozoites delivered to human volunteers by the bite of five infected *Anopheles stephensi* mosquitoes. The *P. falciparum* parasites are provided by the Walter Reed Army Institute of Research and produced as described in WRAIR BB-MF #5855.

4.2 Dosing Schedule

The study intervention is a single challenge episode in which participants will be inoculated with sporozoites on day 0 by the bite of five infected

mosquitoes allowed to feed on the participant's forearm for five minutes. Multiple iterations may be required to ensure sufficient sporozoite inoculation based on semi-quantitative assessment of mosquito blood meal and salivary gland sporozoite load as described in section 4.4.

4.3 Treatment Assignment

All six participants will be assigned to the same treatment intervention.

4.4 Preparation and Administration

The *P. falciparum* parasites are provided by the Walter Reed Army Institute of Research and produced as described in WRAIR BB-MF #5855. The parameters for production and evaluation of the challenge material described below are approximate and will be specifically defined in the master batch record (MBR) for each parasite lot manufactured under GMP. Parasite cultures are expanded and infected mosquitoes produced at trial quantities under phase-appropriate GMP conditions in the CeMPMIR at Seattle BioMed, per standard procedures. Briefly, parasitized erythrocytes received from the WRAIR Master Cell Bank (MCB) will be thawed, expanded and maintained in a continuous culture with medium containing 5% normal human erythrocytes and 10% normal human serum. Blood products used for malaria culture and mosquito infection are obtained from FDA-registered commercial blood suppliers and have been commercially tested for HIV, hepatitis B, C, and syphilis.

The mosquitoes used to deliver the challenge material are a laboratory-reared *Anopheles stephensi* strain originally received from WRAIR. This species has a long history of successful use in human challenge studies for transmission of malaria parasites to volunteers via infectious bites.(4, 11) Mosquitoes are reared per CeMPMIR standard entomology procedures and infections performed under strict containment conditions in the secure insectary of the CeMPMIR at Seattle BioMed. Mosquitoes are infected with *P. falciparum* by standard membrane feeding methods on cultures derived from the WRAIR MCB containing a sufficient proportion of mature gametocytes. Infected mosquitoes are stored at 27°C and 75% humidity for up to 19 days after infection with *P. falciparum* gametocytes. Eight to ten days after infection, a representative sample (5-10) will be removed from each container of infected mosquitoes and evaluated for the presence of oocysts in the mosquito midgut.

Approximately 16 days after infection, a second sample of 10-20 mosquitoes will be removed from each carton and dissected to assess the sporozoite load in their salivary glands. If either midgut or salivary infections are sub-optimal for challenge, the infected mosquitoes will be destroyed and the challenge material will be produced from a back up batch initiated as described 4-5 days after the first round of mosquito infection. In the unlikely event that both the primary and back up batch of infected mosquitoes are assessed to be sub-optimal for challenge, infected mosquitoes will be transferred from the WRAIR Entomology Unit to conduct the challenge.

Mosquitoes infected approximately 16 to 19 days earlier that are assessed to contain adequate *P. falciparum* sporozoites in their salivary glands will be used for the participant challenge. On the day of challenge five infected mosquitoes placed in a screen-covered one pint carton will be allowed to feed on the forearm of study participants for five minutes. Each study participant will have their own container uniquely labeled with the protocol number, participant identification number (PTID), date of mosquito infection and mosquito batch number. Following the five minute feed, mosquitoes will be evaluated to confirm both and adequate blood meal, as evidenced by the presence of a blood meal in the abdomen, and the presence of adequate numbers of sporozoites in their salivary glands by individual dissection. Paired salivary glands of each fed mosquito are transferred to a microscope slide and squashed with a cover slip to release the sporozoites. The sporozoite load is rated under microscopy according to the following scale based on previously described methods for scoring the mosquito salivary gland load:(4)

**Salivary gland score**	**Approximate number of sporozoites**
0	no sporozoites observed
+1	1-10 sporozoites observed
+2	11-100 sporozoites observed
+3	101-1000 sporozoites observed
+4	>1000 sporozoites observed

Only mosquitoes with a salivary gland rating of +2 or more are considered infective. If required, additional infected mosquitoes will be allowed to feed on the participant until a total of five infected mosquitoes per participant with a minimum +2 salivary gland score have taken a blood meal. Additional mosquitoes are added to a carton depending on the

number of remaining infectious bites required. Several iterations may be required to ensure each participant receives 5 infective bites.

4.5 Storage and Handling

Parasitized erythrocytes received from the WRAIR MCB will be stored in liquid nitrogen until released for use in the production of infected mosquitoes as described above. Infected mosquitoes are stored under eight levels of containment in the secure insectary at the CeMPMIR. Mosquitoes in screened 32 ounce containers are stored in within screened cages. Up to 16 screened cages are stored in incubators within the secure insectary behind a locked, screened door and limited access entrance with security airlock. Following infection with *P. falciparum* gametocytes, the infected mosquitoes are maintained for up to 19 days at 27°C and 75% humidity. All mosquito manipulations are performed in an enclosed acrylic Plexiglas glove box. On the day of participant challenge five infected mosquitoes are removed and placed in a screen-covered container labeled with the protocol number, participant’s study identification number (PTID), date of challenge and mosquito batch number. One container for each participant will be transferred to the challenge room within the secure facility. The mosquitoes will be allowed to feed at room temperature on the participant’s forearm overlaying the screened container and covered with a towel to simulate darkness. After five minutes, fed mosquitoes will be harvested and dissected as described above.

4.6 Accountability (Release, Dispensing and Return of Investigational Product)

4.6.1 Release and Dispensing

Parasite stocks received from WRAIR will be released by the Quality Assurance Unit (QAU) for use in production of the challenge material per Seattle BioMed procedures for product release and batch record review. Mosquito batch numbers will be entered into the participant’s case report form. Infected mosquitoes will be released for challenge by the QAU following evaluation of the salivary gland sporozoite load from a representative sampling of the infected mosquitoes on the day prior to challenge, conducted by insectary staff.

4.6.2 Return of Study Product

Challenge material will be consumed as described above. All remaining infected mosquitoes not used for challenge will be destroyed or used for Quality Control (QC) assessments.

4.7 Adherence Assessment

Participant compliance for study product administration will not be an issue because the challenge material will be administered by clinical trial staff.

4.8 Toxicity Management

Participants will be monitored for acute reactogenicity following the challenge as described in section 5.2.2. Participants who develop pain or itch at the site of inoculation will be prescribed topical hydrocortisone 1% or a topical antihistamine to use twice daily for 3 days following challenge. Oral antihistamines may also be prescribed for treatment of local or systemic symptoms following challenge.

Systemic symptoms in the acute reactogenicity period will be assessed and graded per the Adverse Event Toxicity Grading Tables (Appendix C) and treated by the study clinician as outlined in section 6.5.

Trained medical staff will be on site to provide emergency medical care if necessary.

5.0 Study Procedures and Assessments

Study procedures are listed in Table 1 and 2. All pre-enrollment and post-enrollment procedures are performed on all participants (unless otherwise noted) at the time points indicated in Appendix A, with associated laboratory assays and blood draw volumes specified in Appendix B. For some visits, study timepoint windows are allowed. The windows are defined in Table 3.

Participants are considered to be enrolled into the trial only upon receipt of experimental challenge on day 0.

Table 1: Study Screening Procedures

Pre-enrollment		
**Clinical evaluation**	**Counseling**	**Other screening procedures**
Medical history	Protocol informed consent	Participant ID card (issued D0)
Complete physical exam[1]	HIV pre- and post-test counseling[2]	Confirmation of eligibility
Abbreviated physical exam[3]	Assessment of understanding	
Concomitant medications	Pregnancy prevention counseling	
Cardiac Risk Assessment[4]		
**Laboratory Assays**		
CBC with differential	Screening Chemistry Panel:	Urine dipstick/urinalysis
HIV ELISA +/-	Serum creatinine	Females:
HIV Western Blot confirmation	AST/ALT	Pregnancy testing
Hepatitis BsAg	Total bilirubin	(urine or serum βhCG)
Hepatitis C Ab	Alkaline phosphatase	

Table 2: On-Study Procedures

Post-enrollment		
**Clinical evaluation**	**Intervention and monitoring**	
Abbreviated physical exam[3]	Malaria challenge	
Concomitant medications	Pre-patent period follow up	
Reactogenicity Assessments	Hotel Phase Monitoring	
Intercurrent illness/ adverse experience	Treatment with chloroquine	
Pregnancy prevention compliance	Specimen collection and storage	
Assessment of understanding (if necessary)	Post-treatment follow up	
**Laboratory Assays**		
Safety Assessment	Malaria diagnostics	Immunology Assessment
CBC	Blood smear	Cell Mediated Immunity Assays (CMI):
Chemistry panel:	RT-PCR (exploratory)	IFN-γ ELISpot
AST/ALT		Humoral Assays:
Serum Creatinine		Binding ELISA (CSP, AMA-1, MSP-1)

[1] Includes vital signs (temperature, blood pressure, respiratory rate, heart rate, height, weight), HEENT, neck, lymph nodes, chest, heart, abdomen, extremities, skin and neurologic exam.
[2] Includes follow up contact to report results to participant
[3] Includes vital signs and symptom-directed physical exam
[4] Includes screening ECG and NHANES I cardiac risk assessment

Table 3: Visit Windows

Visit	Study Day	Procedure	Visit Window
1.0	D -56 to -1	Screening	N/A
2.0	D0	Enrollment/Challenge	N/A
3.0	D1	Clinic Follow-up	N/A
4.0 – 7.0	D5 to D8	Clinic Follow-up	N/A
8.0 – 17.0	D9 to D18	Hotel Phase	N/A
18.0	3 days post hotel d/c	Phone Follow-up	+ 2 days
19.0	D28	Clinic Follow	+/- 3 days
20.0	D35	Clinic Follow	+/- 3 days
21.0	D42	Clinic Follow	+/- 3 days
22.0	D49	Clinic Follow	+/- 3 days
23.0	D56	End Visit	+/- 3 days
P1	3 months	Clinic Follow-up	+/- 7 days
P2	6 months	Clinic Follow-up	+/- 7 days
N/A	6 month phone call	SAE Follow-up	+/- 2 weeks

5.1 Pre-enrollment procedures

5.1.1 Screening

Screening procedures are done to determine eligibility and to provide a baseline for comparison of safety data. Screening procedures include the informed consent process, assessment of understanding and laboratory and clinical assessments of eligibility and collection of specimens for long term storage. Screening may occur over the course of several visits up to and including Day 0, prior to the challenge. All inclusion and exclusion criteria must be assessed within 56 days before enrollment, unless otherwise specified in the inclusion/exclusion criteria.

As described in Section 3.3, the CTC may employ recruitment efforts such as a telephone pre-screen prior to the participant consenting, but the protocol informed consent must be signed before any protocol-specific procedures to determine eligibility are performed.

5.1.2 Informed Consent

Informed consent encompasses all written or verbal study information the MCTC study staff provides to the participant, before and during the trial.

All informed consent discussions will be documented by the study staff in the participant's source documentation.

Written informed consent will be obtained only by MCTC study staff trained in the protocol and designated on the study signature log, using the protocol-specific Informed Consent Form approved by the local IRB/EC and developed and administered in accordance with MCTC policies and procedures, local IRB/EC requirements, federal guidelines 21 CFR 50.20, 45 CFR 46.116 and the ICH E6 Guidance section 4.8.10.

Qualified study staff will explain the study to potential participants using both verbal and written information where possible. The participant will be given ample time to review the Informed Consent Form, ask questions about the study and discuss with others prior to signing and dating the form. All questions will be documented and answered to the participant's satisfaction. Key concepts will be reviewed with the participant and their understanding periodically assessed through open ended questions about the study. HIV testing consent will be included in the protocol specific consent as it is part of the screening procedures and eligibility criteria. A separate consent will be presented to participants for optional participation in long term follow-up assessments of immune response to malaria infection as an extension of this protocol. This extension to the main study includes 2 additional visits at 3 and 6 months post-challenge for immunology assessment as described in Section 5.4.

Participants will be provided with a copy of all consent forms that they sign. The original signed and dated copy will be kept on file in their study binder, stored in locked cabinets within the MCTC.

The informed consent process continues throughout the study. Key study concepts will be reviewed periodically with the participant and the review will be documented. At each study visit, study staff will review the procedures and requirements for that visit and periodically provide an overview of the remaining visits. Any new information learned by the Investigator that might affect the participant's decision to stay in the trial will be shared with trial participants. If necessary, participants will be asked to sign a revised informed consent form.

To ensure the participant fully understands the study, an Assessment of Understanding questionnaire will be administered to the participant prior

to enrollment and challenge to document their understanding and to highlight areas that need additional discussion or clarification. All incorrect responses will be reviewed with the participant, and s/he must verbalize understanding of all questions answered incorrectly. A score of 80% correct is required for enrollment. For participants scoring less than 80%, study staff may choose to review study principles and informed consent details again with participant and reassess comprehension with a repeat Assessment of Understanding questionnaire. At the discretion of the Investigator, any participant whose comprehension is questionable, regardless of score, may be excluded from enrollment. This process and the participant's discussion of understanding will be documented in the source documentation.

5.1.3 Clinical Evaluations:

Clinical assessment procedures are conducted both the pre- and post-enrollment as outlined in Tables 1 and 2 and in Appendix A. Procedures performed prior to enrollment are used to support documentation of eligibility and the assessment that the participant is generally in good health. All procedures will be documented in the participant's source documentation and the relevant details reported in the appropriate Case Report Form (CRF) upon enrollment. Pre-enrollment clinical procedures include:

a) Complete medical history obtained by the study clinician including review of medication history and concomitant medications (outlined in detail in section 5.1.4 below), including prescription and nonprescription drugs, vitamins, topical therapies, alternative/complementary medicines (e.g., herbal supplements), recreational drugs, and vaccinations.

b) Review of standard demographic data for the participant including sex, age, race, and ethnicity.

c) Complete physical examination including height, weight, vital signs (blood pressure, heart rate, respiratory rate) as well as HEENT, neck, lymph nodes, chest, heart, abdomen, extremities, skin and neurologic exam. Examination of other areas as directed by symptoms may be performed.

d) An abbreviated physical exam may be performed on follow up screening contacts prior to enrollment as indicated, to evaluate any new reported symptom or illness. The abbreviated physical exam is also performed during post-enrollment scheduled follow up visits as indicated. The abbreviated physical examination includes vital signs as noted above and a symptom-directed physical exam. Both the complete and any abbreviated physical exam done prior to enrollment will be documented in the participant's source documentation and will support evaluation of the participant's eligibility.

e) Cardiac risk assessment based on the National Health and Nutritional Examinations Survey (NHANES I)(22) cardiovascular risk assessment criteria (Appendix D) and screening 12 lead electrocardiogram (ECG). Screening ECGs will be performed by study staff and interpreted by an expert cardiologist. Only volunteers classified as low risk by the NHANES I criteria (Appendix D, blue and green sections) who have a non-clinically significant ECG as determined by the study cardiologist will be eligible for enrollment.

f) Clinical laboratory assessment as outlined in Table 1 and Appendix B including CBC with differential, HbsAg, Anti-HCV, Chemistry panel (ALT, AST, alkaline phosphatase, total bilirubin and creatinine), screening HIV test, urine dipstick and/or urinalysis as indicated and for female participants, a urine or serum pregnancy test.

g) HIV counseling and testing including follow up contact with participant to report the results and referral for appropriate medical care in compliance with Washington State Department of Health (DOH) guidelines.

h) Counseling on pregnancy prevention during the study including assessment and counseling of behaviors that could lead to pregnancy and appropriate methods for prevention as defined in the inclusion/exclusion criteria.

5.1.4 Concomitant Medications

All participants will be asked to report any medication and history of medications at each study visit/contact, including screening and post-

enrollment visits. All reported medications will be recorded in the source documentation for that visit and, once the participant is enrolled, will also be recorded in the appropriate CRF. Documentation will include the trade name and/or generic name of the medication, dosing, route of administration, start and end dates of treatment and indication for use. This includes any concomitant medication administered or taken once enrolled

5.1.4.1 Prohibited Therapy

As noted in section 3.2, the following medications and/or treatments are exclusion criteria for participation in the study and/or prohibited while on study except as noted below. Data from participants who are enrolled and subsequently receive prohibited therapy may be excluded from analysis as described in Section 8.2.

a) Prior receipt of an investigational malaria vaccine

b) Malaria chemoprophylaxis with chloroquine within 5 months prior to malaria challenge, with atovaquone/proguanil within 2 months prior to malaria challenge or with mefloquine within 30 days prior to malaria challenge

c) Antimalarial medications other than the treatment administered by study staff

d) Medications known to interact with chloroquine and/or atovaquone/proguanil during the study period including cimetidine, neostigmine, rifabutin, rifampin, metoclopramide; additionally, antacids or kaolin within 4 hours of chloroquine administration

e) Systemic antibiotic therapy with anti-malarial effects (i.e., tetracyclines for dermatologic conditions, sulfa drugs for recurrent urinary tract infections, etc.) during the study period. Topical antibiotics are not prohibited.

f) Any investigational or non-registered drug or vaccine while on study

g) Immunosuppressive medications (>0.5mg/kg/day of prednisone or equivalent corticosteroid therapy for greater than 14 days) within 6 months prior to challenge or within 21 days after challenge (e.g., oral/parenteral corticosteroids, and/or cytotoxic medications). Corticosteroid nasal spray for allergic

rhinitis; and topical corticosteroids for mild, uncomplicated dermatitis are not prohibited.

h) Any vaccination received within 30 days post-challenge, except where deemed medically necessary, (e.g. rabies vaccination).

i) Blood products or immunoglobulins received within 120 days of challenge. These are not prohibited while on study if deemed medically necessary.

5.1.4.2 Allowed Therapy

All other medications are allowed, including medications prescribed or recommended by the study investigators for treatment of signs and symptoms attributable to malaria challenge and/or infection.

5.1.5 Determination of Eligibility

Eligibility for the study will be determined based on results of the screening procedures noted above. Only participants who have provided written informed consent, demonstrate understanding of the purpose of the study, are documented to be in good general health and meet all inclusion/exclusion criteria within 56 days of enrollment will be considered eligible for enrollment.

Eligibility of study participants will be verified by at least two study staff with an eligibility checklist prior to enrollment. Final eligibility determination will be the judgment of the Principal Investigator.

5.2 Post-enrollment procedures

All post-enrollment procedures are outlined in Table 2 and will be performed at the scheduled study visits indicated in Appendix A or as necessary at interim visits for assessment of adverse events and/or to ensure participant safety and study compliance.

5.2.1 Enrollment and Challenge

A total of six eligible participants will be enrolled into the study. Participants are considered to be enrolled into the trial only upon receipt of experimental challenge on day 0.

On the day of challenge, eligibility criteria and checklist will be reviewed for each participant to ensure previously determined eligibility status has not changed. A pre-challenge clinical evaluation including vital signs and a symptom-directed physical exam will be performed to serve as a baseline for reactogenicity assessments. For female participants, a urine pregnancy test will be administered on the day of challenge, prior to challenge regardless of whether pregnancy testing was performed as a part of prior study visits. Each participant will be issued a participant identification card that outlines their participation in the trial, the investigational product including known drug sensitivity of the parasite, and investigator contact information.

The malaria challenge will be conducted per the well-established challenge model(4) as described in section 4.4 and 4.5. Briefly, each participant will receive bites on their non-dominant forearm from five *P. falciparum* infected *A. stephensi* mosquitoes within the MCTC challenge room located within the secure Insectary at Seattle BioMed. Participants will be monitored by study staff for 30-45 minutes following the challenge for assessment of acute local and systemic reactogenicity as noted below.

5.2.2 Reactogenicity Assessment

Reactogenicity data will be collected in the acute post-challenge period and for five days following the challenge or until resolution of symptoms. Reactogenicity assessments include solicited adverse events described in section 6.1.2 and include both systemic signs/symptoms and local reactions proximal to the site of sporozoite inoculation. Reactogenicity assessments will be performed by CTC staff in the acute period (30-45 minutes) following challenge and on day 1 post-challenge, then by the participant daily for 3 days (day 2-4) and by the CTC staff at the day 5 post-challenge visit. Participants are required to complete diary forms of solicited adverse events daily on days 2-4 post-challenge.

Participants will be issued a symptom diary and thermometer and will be instructed how to record post-challenge reactogenicity assessments. Participants will be contacted daily by phone on days 2-4 following

challenge and will present to the MCTC for evaluation on day 5 to complete the post-challenge reactogenicity assessment as outlined in Appendix A. For any solicited or unsolicited symptoms that are greater than mild, the participant will be evaluated within 24 hours by the study clinician unless the symptoms are resolving or have resolved in that period. Participant diaries and reactogenicity data from days 2-4 will be collected and reviewed with the participants on the day 5 evaluation. Diary cards will be considered source documentation and will be kept in the participant's study records. All solicited and unsolicited adverse events will be graded for severity per the protocol established toxicity grading tables (Appendix C) and relationship to challenge determined as defined in Section 6.2.2, and will be recorded in the appropriate CRF(s). Solicited and unsolicited signs or symptoms persisting at day 5 will be followed to resolution and recorded in the appropriate CRF.

5.2.3 Malaria Assessment and Treatment

Peripheral blood films are the gold standard for diagnosis of malaria infection. While other methods are commercially available for point-of-care diagnostics, these are generally performed on a symptomatic participant seeking medical care and therefore, likely at a much higher parasitemia. Nucleic acid based methods of detection and quantification of malaria parasites have not been consistently validated thus their use in malaria challenge studies generally remains limited to exploratory endpoints in support of blood smear data.

Experience from previous malaria challenge studies demonstrates that the pre-patency period in non-vaccinated infectivity controls averages 9-12 days post-challenge with a range between 7-23 days(17). Participants will be evaluated daily from day five post-challenge through the Hotel Phase, with clinical evaluation and daily peripheral blood smears as described below. Evaluations on days five through eight will occur at the CTC. Starting from day 9 post-challenge, participants will enter the Hotel Phase of the study, whereby participants are housed with study staff in a local hotel allowing for close observation during hours when the CTC is closed, including nights and weekends. This approach has been the standard practice for challenge studies conducted at WRAIR and allows immediate assessment of any possible symptoms of malaria by investigators on site. Participants are required to stay in the hotel overnight but may otherwise come and go to carry out their normal daily activities, checking in each

morning and night with study staff for assessment of malaria related symptoms and blood smears. Study staff, including a study nurse will be on site 24 hours a day during the entire Hotel Phase. A clinician investigator will remain on site overnight, and will be present frequently throughout the day and otherwise accessible by phone or pager.

From day five, daily blood smears will be performed to assess the development of peripheral parasitemia, and the remaining blood sample used for 1 of 2 daily assessments for exploratory detection of subpatent parasitemia and parasite densities by nucleic acid sequence based amplification and real-time polymerase chain reaction (RT-PCR). Participants will be contacted each afternoon to report the results of their blood smears.

Giemsa stained thick blood smears are prepared in duplicate according to standard challenge procedures(8) outlined in further detail in Section 5.5.3. Smears are examined microscopically using a high power oil immersion objective by trained study microscopists, and the results reported to the study investigator. At least 200 high-power fields (hpf) are scanned for the presence of malaria parasites. This method allows for detection of a parasite density of approximately 3 parasites/µl, often before participants become symptomatic for malaria. For symptomatic volunteers, at least 1000 hpf are evaluated. Additional blood smears may be prepared if the participant develops fever at other times. Slides are considered positive if at least 2 parasites per slide are identified and confirmed by either a second microscopist or clinical investigator.

Participants will be treated upon confirmation of a positive blood smear with standard oral dose of chloroquine phosphate (600 mg cholorquine base initially, followed by 300 mg chloroquine base 6, 24 and 48 hrs later) under direct observation. If the participant vomits within 30 minutes of ingestion, the dose will be repeated. The NF54 strain of *P. falciparum* has documented sensitivity to chloroquine. This regimen is considered curative and has been 100% effective in treating participants of prior challenge studies at WRAIR and the Naval Medical Research Center (NRMC) using this parasite strain. In case of chloroquine intolerance, alternative treatments with other FDA-approved anti-malarial drugs, including atovaquone/proguanil, quinine, and doxycycline will be available to treat infected participants. Treated participants will continue

to stay overnight at the hotel until 3 consecutive blood smears at least 12 hours apart are negative and all symptoms of malaria have resolved. Thereafter they may leave the hotel and will be contacted by study staff daily for three days for symptom review. Participants will be seen in the CTC for evaluation of any new symptoms that are greater than mild occurring after discharge from the hotel.

Based on prior experience, the maximum length of stay in the hotel for any participant should be approximately 10 days. If any participant has not developed patent infection within 18 days following challenge, they will be treated presumptively with FDA approved dosing of Malarone (atovaquone/proguanil), known to be effective on the liver stage, and monitored for further signs and symptoms of malaria in daily follow up at the CTC for 3 days with repeat peripheral blood smears until 3 consecutive blood smears post treatment remain negative.

5.2.4 Toxicity Management

Symptoms assessed to be related to malaria challenge and infection will be treated by the study clinician as needed with standard dosing regimens of FDA-approved medications including but not limited to: topical steroids or antihistamines, systemic antihistamines, anti-pyretics, anti-emetics and anti-diarrheals. History of allergy to any of these medications will be excluded prior to administration. Medications administered will be recorded in the participant source documentation and concomitant medication CRF and the symptom(s) treated will be graded per the protocol toxicity table (Appendix C) and recorded in the appropriate CRF. If necessary, the study clinician will refer the participant to appropriate medical care or for hospitalization as noted in section 10.6.

5.2.5 Follow up

Following completion of treatment and demonstration of cure, participants will be followed weekly until the end of the study. Participants will be followed for a total of 56 days post enrollment per the study visit schedule and procedures outlined in Appendix A, and laboratory safety and immunology assessments in Appendix B. At the termination of the study, participants will be advised to contact the study investigators or their personal physician to report any symptoms possibly related to malaria infection within one year of completion of the study. However, in prior

experience at WRAIR, no challenge participant has ever experienced a relapse or recrudescence as these do not occur following adequate treatment for patent *P. falciparum* infection and chloroquine treatment for patent infection is considered curative.

All participants will be contacted by telephone 6 months after the malaria challenge to elicit the onset of new serious adverse events (SAEs), chronic illnesses and other medically significant conditions.

Separately, participants will be invited to participate in periodic long term follow up immunology assessments at 3 months and 6 months post-challenge under a separate consent as noted in Section 5.4.

5.3 Visit-by-Visit

Study procedures at each scheduled visit are outlined in Appendix A and B. Detailed study procedures by visit are included in a visit checklist for each scheduled study visit, located in the participant's study binder. Visit checklists are completed for each participant's visit and become part of the participant's source documentation.

5.3.1 Interim Contacts and Visits

Occasionally it will be necessary for a participant to be evaluated between study visits for evaluation and/or follow up of acute symptoms and adverse event assessment. Interim visits will be documented in the participant's source records as per scheduled visits and assigned a visit number code based on the most recent study visit (e.g., 2.1, 2.2, etc.).

5.4 Study Extension

In an effort to qualify long term immune responses to malaria infection, participants will be invited to participate in follow up immunology assessments at day 84 (3 months) and day 168 (6 months) post challenge. Participation in this extended follow up will be conducted under a separate informed consent for biologic sampling as an extension to this protocol, and results reported separately. Participants will present to the CTC for review of concomitant medications, interim malaria exposure history (e.g. by travel) and collection of blood samples for immunology assays only. Participants receiving immunosuppressive medications, vaccinations, blood products, immunoglobulins, or any other

investigational or non-registered drug as described in Section 5.1.4.1 or malaria exposure by travel during the interval period since the last study visit will not be invited to participate in follow up immunology assessments.

5.5 Biological Laboratory Samples

Pre- and post-enrollment laboratory assessments are outlined Appendix A and B. Blood sample collection will be conducted by qualified staff at the MCTC. Required blood volumes and tube types for sample collection are specified in Appendix B. Additional sampling may be required for adverse event assessment and follow up, and would represent minimal increase in the total volumes listed in Appendix B. The total blood volume drawn from any participant will not exceed 500 ml in any 56 day period. If blood volume collection is limited, specimen collection will be prioritized for safety and diagnostic assays, followed by additional endpoint assays and specimen storage.

5.5.1 Clinical Laboratory Evaluations

Routine diagnostic laboratory assays will be performed at the clinical laboratory at the University of Washington or another local CLIA certified laboratory. Normal limits for diagnostic assays will be defined by the clinical lab or institution and may be adjusted by the clinical laboratory in the event of a change to their diagnostic assay. All laboratory normals are reported in the study laboratory manual.

Clinical laboratory evaluations for eligibility determination include:

a) Complete blood count (CBC) with white blood cell differential
b) Serum creatinine (Cr)
c) Aspartate Aminotransferase (AST)
d) Alanine Aminotransferase (ALT)
e) HIV assay (3rd generation ELISA with Western blot confirmation)
f) HBsAg ELISA
g) Anti-HCV Antibody (3rd generation ELISA with immunoblot confirmation)
h) Urinalysis (in the event of an abnormal urine dipstick test)

Urine β-hCG testing will be performed at the clinical trial unit using an FDA-approved urine pregnancy test kit.

Urine dipstick testing will be performed at the site using an FDA-approved test. Microscopic and macroscopic urinalysis will be performed by the diagnostic laboratory in the event of an abnormal urine dipstick test.

Laboratory assessments for safety endpoints include measurement of CBC, serum Cr, and AST/ALT performed on the day of first positive blood smear, day 35, day 56, and as required for AE assessment by the study clinician.

Results of diagnostic labs used for screening and follow-up will be stored in the participant case record and reported on the appropriate CRF.

5.5.2 Immunologic Assays

Laboratory assays to assess immune response to malaria infection will be performed in the MCTC immunology core laboratory at Seattle BioMed according to standard laboratory procedures briefly outlined below. These assays include:

a) Antibody ELISA to assess serum antibody levels to known liver and blood stage antigens including CSP, MSP-1, and AMA-1.

Briefly, microtiter plates will be coated with the relevant antigens. After an overnight incubation, diluted sera and controls will be added to the plate, followed by an incubation with Horseradish peroxidase-conjugated anti-human IgG . Captured IgG antibody is detected using an OPD (o-phenylenediamine) dihydrochloride substrate. Samples are analyzed on an ELISA plate reader to determine levels of bound antibody.

b) IFNγ ELISpot assays performed on PBMCs.

Briefly, purified PBMCs will be re-suspended at 2×10^6 cells/mL in culture medium containing 5% human AB serum. Triplicate cultures of 100 µl each will be stimulated with LSA-1, CSP or medium alone at 37°C in 96 well microtiter culture plates. Subsequently the cells will be transferred into the corresponding wells of a 96 well ELISpot plate that was pre-coated with 1-DIK (anti-γ-interferon monoclonal antibody). After an18-hour culture at 37°C the cells will be washed and a second γ-interferon-specific biotinylated monoclonal antibody

will be added to the wells. Binding will be detected using a streptavidin-alkaline phosphatase conjugate plus the appropriate substrate. Results will be expressed as the number of spots per 10^6 cells tested.

5.5.3 Special Assays or Procedures

Infectivity endpoints will be assessed by Giemsa stained thick blood smear and by polymerase chain reaction (RT-PCR). Only the peripheral blood smear will be used for the primary diagnostic endpoint and decision to treat the participant as the RT-PCR detection assay is currently an exploratory assay that has not been validated.

Peripheral blood smears (BS) will be collected daily starting from day five until detection of parasitemia on peripheral blood smear, then daily post treatment until three consecutive blood smears are negative. Peripheral blood smears are prepared in duplicate per standard challenge procedures. Briefly, 3 ml of blood is collected by venipuncture into an EDTA containing tube and each smear is prepared using 10 µl of blood spread over a defined 1 x 2 cm rectangle etched on a glass slide, which is then dried on a heat block and stained with a Giemsa stain. Slides are evaluated microscopically for parasites by the study microscopist using a high power oil immersion objective, and the results reported to the study investigator. At least 200 high-power fields are scanned for the presence of malaria parasites. For symptomatic volunteers, at least 1000 high-power fields are evaluated. Additional blood smears may be prepared if the participant develops fever at other times. Slides are considered positive if at least 2 parasites per slide are identified and confirmed by a second microscopist or clinical investigator. Results of blood smears are documented in a laboratory notebook and on the appropriate CRF. Results are reported to the investigator and to the participant as they become available.

The remaining sample collected for BS preparation and one additional sample per day will be used for exploratory endpoint evaluation of sub-patent parasitemia by real-time polymerase chain reaction (RT-PCR) using an assay currently under development at the University of Washington. This assay allows qualitative and quantitative assessment of parasitemia by amplification of the 18s ribosomal subunit RNA gene of *P. falciparum* and detection of copy number by a gene specific fluorescent probe which

is then compared to standardized reference samples with known concentrations of parasite DNA. Similar assays used in previous challenge studies have demonstrated significantly increased sensitivity well below the limit of detection of peripheral blood films and can provide valuable information on parasite replication dynamics and early response to vaccination, infection or drug treatment. Samples from this study will be used to validate the current assay for use in future human challenge studies at the MCTC.

5.5.4 Specimen Handling and Storage

Specimens will be collected and transported to the appropriate laboratory per standard Seattle BioMed procedures for handling and transport of biological specimens and sample collection recorded on the appropriate CRF. Universal precautions will be followed at all times during collection, handling and processing of biologic specimens. Handling and disposal of biohazardous materials will observe Seattle BioMed environmental health and safety (EHS) policies and Occupational Health and Safety Administration (OSHA) regulations.

Clinical diagnostic laboratory samples will be collected and labeled according to local lab requirements and transported to the University of Washington clinical laboratory by study staff. Urine collection for urine dipstick and urine pregnancy testing will be performed as soon as possible after collection according to the manufacturer’s instructions.

Samples collected for blood smear and RT-PCR will be de-identified prior to transport to the microscopist and processed as noted above. All blood smear slides will be archived at Seattle BioMed for future reference.

Specimens for immunology endpoints will be collected in the appropriate tubes, labeled with the participant identification number and processed per standard Seattle BioMed procedures. Whole blood samples for immune assays will be collected and kept at 20-25°C until transfer to the immunology laboratory for isolation of peripheral blood mononuclear cells (PBMC), separation of plasma and serum per standard laboratory procedures. PBMCs will be stored in liquid nitrogen, and plasma and serum will be stored at -80°C until assays are performed. Any sample remaining after completion of the endpoint assays may be used for exploratory assays or stored for future use as described below.

Specimens for long term storage will be collected and processed as above, and stored in the MCTC specimen repository per standard laboratory procedures. Specimens will be labeled with participant identification number (PTID) at the MCTC. A list linking the coded number to the participant's identifying information is maintained in a secure limited access location at the MCTC. As noted in Section 5.5.5 below, participant identifying information will not be shared with specimen repository management. Assay results from research specimens, including those used for immunology endpoints, will not be reported to participants.

5.5.5 Future Use of stored Specimens

The MCTC seeks to evaluate not only the safety and immunology of experimental human challenge in this study, but to further define immune correlates of protection and mechanisms of malaria pathogenesis in general. For this purpose, some specimens from challenge trial participants will be stored for future testing and research related to malaria pathogenesis, detection, immunity and development of improved therapeutics and standardized assays. Participants will be advised as part of the informed consent process that any remaining samples from endpoint assays will be stored for future use along with separate specimens collected for long term storage and future research as outlined in Appendix B. Specimens for long term storage will be labeled with the participant identification number (PTID) at the MCTC and a key to coded specimens will be maintained by the Investigator. The Investigator and specimen repository management will enter into an agreement whereby the investigator will agree not to release personal identifying information and specimen repository management will not request the information per the OHRP DHHS Guidance on Research Involving Coded Private Information or Biological Specimens dated October 16, 2008. Participants will be informed that results obtained from assays done on stored specimens will not be reported to them or linked with identifiable information.

The informed consent is written so that participants may explicitly allow or reject long term storage of their specimens under an addendum consent to the main study. Participants can withdraw their consent for long term storage of specimens at any time, in which case the specimen repository management will be notified and all remaining stored samples will be

destroyed after completion of the study analysis. Withdrawal of consent will be documented in writing by the participant. Participants who withdraw consent for long term specimen storage will remain in the study.

If withdrawing consent for further study participation, specimens collected to date will be retained for endpoint analysis as noted above with unless the participant explicitly states their intent to withdraw consent for the use of their specimens for this purpose.

5.5.6 State Reporting Requirements

Study staff will comply with all applicable local requirements to report communicable diseases identified among study participants to local health authorities. Participants will be made aware of all reporting requirements during the study informed consent process.

HIV is a reportable illness under Washington state Department of Health (DOH) guidelines and Washington Administrative Code (WAC 246-100 and WAC 246-101). Where indicated by relevant risk factors or a positive result, participants will undergo pre- and post-test counseling for HIV as a part of the screening process, including participant follow up to report the results. Positive HIV test results will be reported to the WA DOH and appropriate medical referrals initiated.

6.0 Safety and Adverse Events

Investigators are responsible for monitoring safety of all participants enrolled into the study. Safety will be evaluated by monitoring participants for local and systemic adverse reactions throughout the course of the trial. Safety assessments will include clinical observation and evaluation and monitoring of clinical laboratory parameters. Assessment of adverse events (AEs) will occur at each encounter with the participant. It is the responsibility of the Investigator to ensure all AEs are documented and reported to the Sponsor, Institutional Review Board (IRB) or regulatory authority, according to the following guidelines.

6.1 Definitions

6.1.1 Adverse Event (AE)

An AE is defined as any untoward medical event that occurs in a human participant administered an investigational product regardless of any

potential causal relationship to the intervention. An AE can therefore be any unfavorable and unintended sign (including an abnormal laboratory finding), symptom, or disease temporally associated with the use of an investigational product, whether or not it is considered to be study related. This includes an exacerbation of pre-existing conditions and intercurrent illnesses. Anticipated day-to-day fluctuations of pre-existing conditions, including the disease under study, that do not represent a clinically significant exacerbation are not considered adverse events.

Laboratory or other test abnormalities (ECG, etc.) that are not clinically significant as assessed by the study investigator, will not be considered an AE. In general, an abnormal laboratory or test finding will be classified as an adverse event if one or more of the following criteria are met:

- The abnormality is accompanied by clinical symptoms.
- The abnormality requires additional diagnostic evaluation or medical intervention.
- The abnormality leads to discontinuation from the study.
- The abnormality is considered an AE in the opinion of the investigator.

All AEs must be graded for intensity and relationship to the investigational product as described in Section 6.2.

6.1.2 Solicited Adverse Event

A solicited adverse event is defined as a predetermined event that may reflect safety concerns related to the investigational product. These include post-challenge reactogenicity events and events that can be reasonably expected as part of this study as defined in prior similar studies,(4, 8, 17, 17) including local and systemic signs and symptoms related to the challenge and/or diagnosis of malaria. While not previously established to be related to malaria infection or experimental challenge, for the purpose of this initial challenge study, cardiac symptom review including chest pain will be included as a solicited AE and reported on the malaria CRF.

Solicited adverse events will be captured by participant diary card and/or by direct questioning during the follow-up period with participants.

Solicited adverse events for this study include the following:

Local signs/symptoms at the site of sporozoite inoculation:

1) Pain/tenderness
2) Pruritis
3) Urticaria
4) Erythema
5) Induration/Edema

Systemic signs/symptoms:

1) Fever (oral temperature ≥ 100.4°F/38°C)
2) Chills/Rigors
3) Headache
4) Malaise/Fatigue
5) Myalgia (other than low back pain)
6) Low Back Pain
7) Nausea
8) Vomiting
9) Diarrhea
10) Abdominal pain
11) Arthralgia
12) Non-musculoskeletal chest pain

6.1.3 Unsolicited Adverse Event

Unsolicited adverse events are all events not defined as solicited, reported by participants or identified by study staff at any time during the study period, or any adverse events occurring after day 28. Unsolicited adverse events will be recorded on the AE CRF and their severity and relationship to the investigational product or test article will be assessed by the Investigator as outlined in section 6.2.

6.1.4 Serious Adverse Event (SAE)

An SAE is any adverse experience, regardless of relationship to the investigational product that results in any of the following outcomes:

1) Death during the study period
2) Life threatening adverse experience defined as an event that places the participant at immediate risk of death at the time of the event. This does not include an event that hypothetically might have caused death were it more severe

3) Inpatient hospitalization or prolongation of existing hospitalization defined as at least an overnight stay in the hospital or emergency ward for treatment that would have been inappropriate if administered in the outpatient setting. Note: hospitalization for elective surgery during the study period that is related to a pre-existing condition that did not increase in severity or frequency after initiation of the study is not considered a Serious Adverse Event.

4) Results in a congenital anomaly or birth defect in the offspring of the participant

5) Persistent or significant disability/incapacity defined as a substantial disruption of the participant's ability to conduct normal life functions

6) Any other important medical event that may not result in death, be immediately life-threatening or require hospitalization may be considered a serious adverse event when, based upon appropriate medical and scientific judgment**,** the event may jeopardize the participant and may require medical or surgical intervention to prevent one of the outcomes listed in the definition above. Examples of such medical events include allergic bronchospasm requiring intensive treatment in an emergency room or at home, or blood dyscrasias or convulsions that do not result in inpatient hospitalization.

6.1.5 Unexpected Adverse Event

An unexpected adverse event is any adverse event, the specificity or severity or frequency of which is not consistent with the current investigator brochure; or, if an investigator brochure is not required or available, the specificity or severity of which is not consistent with the risk information described in the general investigational plan or elsewhere. The term "Unexpected", as used in this definition, refers to an adverse event that has not been previously observed (e.g., included in the investigator brochure) rather than events not being anticipated from the pharmacological properties of the investigational product.

6.1.6 Unanticipated Problems

Recent FDA guidance sought to clarify event reporting to IRBs during a clinical trial.(23) Unanticipated problems are events defined for this purpose, including some adverse events as defined below and other types of problems may represent a risk to study participants or other individuals involved in the trial or may impact the integrity of data during the trial. In general, adverse events identified during the study are considered

unanticipated problems if they are unexpected, serious and would have implications for the conduct of the study (e.g., require change in the protocol, inclusion/exclusion criteria, consent, investigator's brochure). Usually an individual AE does not meet this definition since the implication to the study can not be determined with an isolated event.

The following adverse events and other scenarios should be considered as unanticipated problems for the purpose of IRB reporting:

1) A single occurrence of an event that is serious, unexpected, uncommon and strongly associated with investigational product/test article exposure (such as angioedema, agranulocytosis, hepatic injury or Stevens-Johnson syndrome).

2) A single occurrence or a small number of occurrences of an event that is serious, unexpected, not commonly associated with investigational product/test article exposure, but uncommon in the study population (e.g., tendon rupture, progressive multifocal leukoencephalopathy).

3) Multiple occurrences of an AE that, based on an aggregate analysis, is determined to be an unanticipated problem. There should be a determination that the series of AEs represents a signal that the AEs were not just isolated occurrences and involve risk to human subjects (e.g., a comparison of rates across treatment groups reveals higher rate in the investigational product arm verses the control). A summary and analyses must accompany the report to the IRB.

4) An AE that is described or addressed in the investigator's brochure, protocol or informed consent documents, but occurs at a specificity or severity that is inconsistent with prior observations. For example, if transaminase elevation is listed in the investigator's brochure and hepatic necrosis is observed in study participants, hepatic necrosis would be considered an unanticipated problem involving risk to human subjects. A discussion of the divergence from expected specificity or severity must be included in the report to the IRB.

5) A serious AE that is described or addressed in the investigator's brochure, protocol or informed consent documents, but in the study occurs at a clinically significant increased rate compared to the expected rate of occurrence; ordinarily, reporting would only be triggered if there were a credible baseline rate for comparison. A

discussion of the divergence from the expected rate must be included in the report to the IRB.

6) Any other AE or safety finding (e.g., based on animal or epidemiologic data) that would cause the sponsor to modify the investigator's brochure, study protocol, or informed consent documents, or would prompt other action by the IRB to ensure the protection of human subjects. An explanation of the conclusion must accompany the report to the IRB.

7) Breach of confidentiality (e.g., lost or stolen research data that can identify participants).

8) Incarceration of a research participant, when the IRB has not approved the study for prisoners.

9) Complaints from research participants and/or staff about unsafe research (including the research environment) that can not be resolved.

6.2 Classification

All adverse events will be assessed and graded for severity and relationship to study product. Only a qualified clinician Investigator can make this determination by a thorough clinical assessment of all information surrounding the event including any medical intervention required.

6.2.1 Severity/Intensity:

Severity of clinical and laboratory adverse events will be assessed by the study clinician using a protocol defined grading system as represented in Appendix C, adapted from similar toxicity grading tables used for other vaccine clinical trials.(24, 24-26)

For events not included in the protocol defined grading system the following guidelines will be used to quantify severity/intensity:

Grade 1 (Mild): events require minimal or no treatment and cause minimal or no interference with the participant's usual social and functional activities.

Grade 2 (Moderate): events result in low level of inconvenience and cause greater than minimal interference with usual social and functional activities. The event may require limited medical intervention.

Grade 3 (Severe): events result in complete inability to perform usual social and functional activities. Severe events are usually incapacitating and are likely to require medical intervention and/or close follow-up.

Grade 4 (Potentially life threatening): any adverse experience that places the participant, in the view of the investigator, at immediate risk of death from the reaction as it occurred, i.e., it does not include a reaction that had it occurred in a more severe form, might have caused death. A grade 4 Adverse Event is considered serious and will be reported as an SAE.

As defined by the ICH E6 Good Clinical Practice: Consolidated Guidance, the term “severe” is often used to describe intensity (severity) of a specific event (as in mild, moderate, or severe myocardial infarction); the event itself however, may be of relatively minor medical significance (such as severe headache). This is *not* the same as “serious”, which is based on patient/event *outcome* or *action* criteria usually associated with events that pose a threat to a patient’s life or functioning. Seriousness (not severity) serves as a guide for defining regulatory reporting obligations.

6.2.2 Relationship to Investigational Product

All adverse events will be evaluated by the Investigator for their association to receipt of the investigational product and this relationship used to evaluate reporting requirements to regulatory agencies. The Investigator (**only a physician can make this determination**) will assign a causal relationship of the AE to the investigational product using clinical judgment in conjunction with the assessment of a plausible biologic mechanism, temporal relationship between the onset of the event in relation to receipt of the investigational product or test article, and identification of a possible alternate etiology including underlying disease, concurrent illness or concomitant medications. The following classification will be used by investigators to assess the relationship of an AE to receipt of the investigational product:

Not related: The event is completely independent of receipt of investigational product and/or evidence exists that the event is definitely related to another etiology.

Probably Not Related: Likely unrelated to the investigational product or test article. The nature of the event or temporal association with receipt of the investigational product is such that the event is likely to be related to

factors other than the investigational product but a relationship cannot be ruled out with certainty (cause and effect relationship improbable but not impossible).

Possibly Related: An association between the event and the receipt of the investigational product or test article cannot be ruled out. There is a reasonable temporal association, but an alternate etiology such as the participant's clinical status or association with underlying factors including other therapy exists.

Probably Related: A high degree of certainty there is a relationship to the investigational product or test article. There is a reasonable temporal association, and potential alternate etiology or association is not apparent.

Definitely Related: A clear association exists between the receipt of investigational product and the event. Any alternate etiology or association to other factors has been ruled out.

6.2.3 Outcome

All adverse events will be followed to resolution or stabilization as described in section 6.6. AE outcome will be classified as:

Resolved: The participant has fully recovered from the event with no residual effects observable.

Stabilized: The participant has recovered with long term sequelae and/or effects of the event are chronic with low likelihood of these effects changing (improving or worsening).

Ongoing: Effects of the event are still present and changing. The event is not considered stabilized or resolved.

Participant Death: Death is an outcome of an event and not an event per se (sudden death or death of unexplainable causes can be reported but follow-up will be required until cause of death is determined). Death outcomes should be further classified as follows:

> **Death Due to Event:** The event was the primary cause of death (may or may not be the immediate cause of death).
>
> **Death Due to Other Event:** The participant died but the event reported was not the primary cause of death.

6.3 Identification and Documentation of Adverse Events

Adverse event assessment will be performed at each visit and all adverse events will be evaluated by the investigator. These include events observed by study staff or reported by the participant either on direct questioning or voluntarily. Occurrence of an AE may come to the attention of study personnel during study visits and interviews of participants presenting for medical care or on review by a study monitor.

Adverse events will be documented in the appropriate location (source record, participant diary, and/or CRFs) from the time the participant signs informed consent through the end of the study (day 56). Adverse events that occur after informed consent is obtained, but prior to enrollment, will be documented in the Medical History source documentation and reported on the Pre-existing Conditions CRF if the AE persists on enrollment.

Information collected includes a description of the event, date and time (where appropriate) of onset, outcome (time of resolution/stabilization of the event), intensity/severity, and relationship to the investigational product. Changes in the severity of an AE should be documented to allow assessment of the duration of the event at each level of intensity. Adverse events characterized as intermittent require documentation of onset and duration of each episode. Details of any intervention or treatment should be recorded on the appropriate corresponding CRF. See Section 6.4 for instructions for reporting and recording of Serious Adverse Events.

Whenever possible, the investigator should group signs or symptoms that constitute a medical diagnosis into a single event term. For example, cough, rhinitis, and sneezing might be grouped together as *upper respiratory tract infection*. Where a single diagnosis is not appropriate, individual symptoms will be reported as separate AEs.

Solicited adverse events to be recorded as endpoints are described in section 6.1.2. Solicited AEs will be captured by the participant diary and/or by direct questioning during the follow-up period with participants through day 28. Solicited AEs noted during the reactogenicity period will be recorded on the Reactogenicity CRF. Solicited AEs captured through day 28 will be recorded on the Malaria Symptom Questionnaire CRF. All other adverse events through the end of the study will be recorded as unsolicited adverse events and reported on the AE CRF.

Laboratory and/or other testing related adverse events (as defined in section 6.1.1) will be recorded on the AE CRF.

6.4 Adverse Event Reporting

6.4.1 Serious Adverse Events

All Serious Adverse Events (as defined in section 6.1.4) that occur during the study period starting from enrollment through the last study visit will be evaluated by the Investigator, documented in the participant's source documentation and AE CRF and reported on a SAE report form **immediately** (within 24 hours of identification of the event) by email to the Sponsor Medical Monitor, regardless of assessed relationship to the investigational product. Additionally, the Study Monitor and Sponsor Regulatory Representative will be notified by email or telephone that an SAE has been reported to the Medical Monitor.

The Investigator will document all available information regarding the SAE on the SAE Report form. In addition to the information provided in the SAE Report, the Investigator will make available copies of any supporting documentation (e.g., adverse event CRF, concomitant medication CRF, laboratory or diagnostic test results, diaries, and progress notes) that may support pharmacovigilance review and determination of further reporting requirements. Participant personal identifying information must be redacted from the documents and all documents must include protocol and participant number.

Fatal and life-threatening AEs (regardless of suspected causality) must first be reported by telephone as soon as the event is discovered by the Investigator.

Events are reported as outlined above to:

Seattle BioMed Medical Monitor:

Wesley C. Van Voorhis, MD PhD
Telephone number: (206) 543-2447 (office)
(206) 437-1522 (cell)
(206) 982-6384 (pager)
(206) 361-0149 (home)
Fax number: (206) 616-4898
email: wesley@u.washington.edu

Sponsor Representative and Study Monitor:

Susan Lundebjerg
Telephone number: (206) 256-7125
email: susan.lundebjerg@sbri.org

6.4.2 Other Immediately Reportable Events

The investigator must report the following events **immediately** (within 24 hours of identification) to the Medical Monitor, using the SAE Report form and via email or telephone call to the Study Monitor and Sponsor Regulatory Representative.

1) Pregnancy

   Participants who become pregnant during the study period will be followed on study. A pregnancy should be followed to term for outcome, including date of delivery, health status of the mother and child including the child's gender, height and weight. Complications and or abnormalities should be reported including any premature terminations. A pregnancy is reported as an AE or SAE only when there is suspicion that the investigational product may have interfered with the effectiveness of contraception or there was a serious complication in the pregnancy including a spontaneous abortion or an elective termination for medical rationale.

2) Withdrawal of a participant due to an adverse event whether the participant withdraws due to the adverse event or is withdrawn by the Investigator due to an adverse event.

6.4.3 Unanticipated Problems

The Investigator will report all unanticipated problems to the Sponsor Medical Monitor within 48 hours of identification of the problem and will notify the Study Monitor and the Sponsor Representative as outlined in the Study Binder. The Sponsor Medical Monitor will evaluate the relevance and significance of unanticipated problems, in relation to all similar occurrences of the event and the population under study (e.g., cardiac events in an elderly population) and the type of problem (e.g., breach in confidentiality). The Sponsor Medical Monitor may consult with the Trial Steering Committee or the Safety Monitoring committee (SMC) (as defined in Section 9.0) and/or the Sponsor Representative to determine whether an event is reportable to the IRB as defined by their policies and procedures.

6.4.4 Reporting to Regulatory Agencies

As mandated by regulations, the Sponsor must notify regulatory authorities of specified adverse events within a certain timeframe. These include some unanticipated problems and events that are classified as serious, unexpected and where there is a reasonable possibility that the experience may have been caused by the investigational product. In general, events classified as “probably not related” do not meet the definition of reasonable possibility for reporting to the FDA.

After clinical review, the Sponsor Medical Monitor, in consultation with the Sponsor Regulatory Representative will determine the SAEs and unanticipated problems requiring reporting to regulatory agencies.

For trials conducted in the United States, per the Code of Federal Regulations (21CFR 312.32), all serious adverse events that are unexpected and for which there is a reasonable possibility that the event may have been caused by the investigational product must be reported to the FDA and all associated Investigators conducting the clinical trial as soon as possible but in no event later than 15 calendar days after the determination is made. Further, any unexpected fatal or life-threatening experience associated with use of the investigational product should be reported to regulatory authorities by telephone or fax as soon as possible but in no event later than 7 calendar days after the Sponsor’s initial receipt of the information. In order for complete reporting and follow up, the Investigator is responsible for providing any additional information about the event to the Sponsor, as soon as it is available.

6.4.5 Reporting to the Institutional Review Board

The Investigator is responsible for reporting serious adverse events and unanticipated problems involving risk to human subjects or others to the IRB of record according to its’ policy and applicable regulations.

6.5 Treatment of Adverse Events

The Investigator is responsible for ensuring provision of reasonable medical care to participants enrolled or on-study, with follow-up for adverse events, abnormal laboratory/test values and intercurrent illnesses. During participant visits at the clinical trial site, the investigator is required to provide emergency medical care.

Treatment of any adverse event is at the discretion of the Investigator and/or Medical Monitor in accordance with Good Medical Practice. Any treatment should be documented in the participant case record and the

appropriate CRF as applicable. Further details on provision of care for study related injury or illness is described in section 10.6.

6.6 Adverse Event Follow Up

All adverse events including clinically significant laboratory or other testing abnormalities will be followed to resolution. Serious adverse events that are considered related to the investigational product by the Investigator or Sponsor will be followed until resolution, even if the follow up period extends beyond the study period. Resolution is defined as normalization of the abnormality or return to baseline status, or the Investigator has determined the condition has stabilized or that it will remain chronic.

Investigators are not obligated to actively seek SAEs in former study participants however, if an SAE, considered to be related to the investigational product / test article is brought to the attention of the investigator *at any time* following completion of the study, the event will be reported to the sponsor as defined above.

For adverse events that are not serious and have not resolved by the end of the reporting period, every effort will be made to follow the participant clinically, until resolution of the event. For moderate to severe adverse events continuing beyond the last study visit and not considered to be related to the investigational product, the participant will be encouraged to follow up with their primary medical physician to ensure resolution.

6.7 Unblinding Procedures

Blinding is not applicable in the current study.

6.8 Stopping Rules

6.8.1 Participant Discontinuation

As this is a single intervention trial design, participants will not be discontinued from the intervention. However, participants may be withdrawn from the study as noted in section 3.4.

6.8.2 Study Discontinuation

The conduct of this study may be suspended or terminated early by the determination of the Sponsor, the IRB of record, or of the appropriate local or national regulatory authority.

6.9 Monitoring

6.9.1 Medical and Safety Monitoring

A Medical Monitor has been appointed by the Sponsor for oversight of safety in this trial. The Sponsor Medical Monitor will be available to advise the Investigators on trial-related medical questions or problems, and act as a representative for the participants' welfare. Additionally, the Sponsor Medical Monitor or the Investigator may consult with members of the Trial Steering Committee (as defined in Section 9.0) and may ask to convene a Safety Monitoring Committee meeting for review of any safety issue or adverse event. The Sponsor Medical Monitor will review all SAEs and confirm the determination of relationship to the investigational product. The Sponsor Medical Monitor will advise the Sponsor Regulatory Representative in making the determination of adverse events requiring reporting to regulatory agencies. The Sponsor Medical Monitor will also review individual and cumulative participant data periodically throughout the trial to identify concerning AE trends or other safety issues related to the study.

The Trial Steering Committee (TSC) will select three individuals independent of the Sponsor to serve on a Safety Monitoring Committee (SMC) whose activities are defined in a written charter that includes procedures for conducting meetings, review criteria, reports and communication with the site and sponsor. The SMC's primary responsibility will be to ensure participant safety and advise the Sponsor Medical Monitor and the study Investigators. As needed, the SMC will periodically review individual and cumulative participant data on safety for evidence of study-related AEs, adherence to the protocol, and factors that may affect outcome or study data such as protocol violations and participant withdrawal. The Sponsor Medical Monitor and Investigator are responsible for ensuring that the SMC is aware of all new relevant safety information.

Because of the single intervention trial design, SMC review will not be required to approve further interventions. For this trial, the SMC will have an organizational meeting and one after the conclusion of the hotel phase or at the request of the Sponsor, Sponsor Medical Monitor, Trial Steering Committee and/or study Investigators for review of safety issues or SAE review as well as study conduct.

6.9.2 Study Monitoring

To ensure protection of study participants, compliance with the protocol, and accuracy and completeness of records, the Sponsor will monitor all aspects of the study for compliance with Good Clinical Practices (GCP) and applicable government regulations. A Study Monitor appointed by the Sponsor will periodically review the individual participant records, including consent forms, CRFs, supporting data, laboratory specimen records, and progress notes/source documentation. The monitor will inspect regulatory files to ensure that regulatory requirements are being followed and may also inspect entomology challenge records as applicable. The Investigator will be given reasonable notification prior to the monitoring visit in order to ensure study staff are available to discuss the study.

7.0 Data Handling and Record Management

7.1 Data Management Responsibilities

Seattle Biomedical Research Institute (Seattle BioMed) Data Management Center or designated third parties will be responsible for all data processing, including receiving, entering, cleaning, coding, reconciling, storing and transferring of data for statistical analysis and report writing.

7.2 Data Collection Process

7.2.1 Types of Data

Clinical assessments (including adverse events), routine and safety laboratory assessments and biological sampling data are collected throughout the clinical study and recorded on source documents in the participant’s medical record. Specific types of data collected include physical examination findings, vital signs, laboratory reports, electrocardiogram reports, solicited and unsolicited adverse events, concomitant medication use and participant diaries.

7.2.2 Data Capture Methods

This study will use DataFax, a clinical data management software package, for electronic data capture. The study staff will record clinical information on source documents and transcribe this information on

sponsor-approved printed Case Report Forms (CRFs) according to CRF completion guidelines in the study binder. Completed CRFs are faxed to the Seattle BioMed data management center via a dedicated facsimile machine.

The data management center staff will receive faxes and enter data in a secure, restricted access DataFax database that is 21CFR11 compliant, maintaining an audit trail of entries, changes and deletions of data by date, time and user. The data management staff or designated third parties will also clean and code the data, resolve discrepancies and lock the database according to Seattle BioMed data management procedures.

7.3 Source Document Requirements

Source data are all information necessary for reconstruction and evaluation of the trial, including original records of clinical and laboratory findings, observations or other activities. All study data must be verifiable to the source documentation. A file containing all the source documents will be maintained for each study participant at the study site including documentation of all observations and other data pertinent to the investigation on each individual administered an investigational product or used as a control in the trial. Documentation includes the case report forms and supporting data including, signed and dated consent forms, and any medical records (e.g., clinic or office charts, progress notes of the physician, the individual’s hospital record, nurses’ notes, pharmacy dispensing records, laboratory reports, test results, including data reports from automated instruments, radiology studies, etc.).

In addition, an investigator is required to maintain adequate records of the disposition of the investigational product or test article, including dates (receipt, dispense, return), quantity, and use by participants.

7.3.1 Access to Source Documents

CRFs, source documents, and other supporting documents will be kept in locked cabinets within the MCTC. Source documentation will be available for review to ensure that the collected data are consistent with the CRFs. Sponsor representatives, auditors, the IRB of record and regulatory authorities will be permitted to review, copy and verify all

medical and research records related to this study as part of their responsibility to protect human subjects in clinical research. Upon request of the sponsor representative, monitor, IRB, or regulatory authority, the investigator/institution will make available for direct access all requested trial-related records. Participant personal identifying information will be removed before photocopying.

7.4 Required Records

7.4.1 Essential documents

Essential documents are documents that individually and collectively permit evaluation of the conduct of a study and meet regulatory requirements. The following documents are required and will be maintained by the sponsor according to Seattle BioMed procedures:

1) FDA Form 1572 or equivalent regulatory authority document
2) CVs and licenses, if applicable, for all personnel listed on FDA Form 1572
3) Financial Disclosure form
4) IRB approvals (protocol, amendments, informed consents, advertising), progress reports and correspondence
5) Investigator Brochure (and updates)
6) Source documents (including signed informed consent forms) and completed case report forms (including query resolutions)
7) Laboratory documentation (certification and normal ranges)
8) Protocol specific training records
9) Logs (Delegation of Responsibilities/Signature Log, Monitor Log, Participant Screening/Enrollment Logs, Participant Identification Codes
10) Serious Adverse Event Reports
11) Correspondence, Notes to File, telephone contact reports, protocol deviation reports
12) Investigational product accountability records
13) Manuals (e.g., study binder, laboratory, pharmacy)

7.4.2 Other

N/A

7.5 Record Retention

Study files will be stored by the Sponsor at Seattle BioMed or other designated secure location off site. Records must be maintained for a minimum of two years after the Investigational New Drug (IND) application is withdrawn or until two years after the related New Drug Application (NDA) is either approved or withdrawn. If no application is to be filed, then the records must be maintained for a minimum two years after the investigation is completed or discontinued and FDA is notified.

7.6 Protocol Deviations

A protocol deviation is defined as any occurrence involving a procedure that did not follow the study protocol, applicable procedures, and/or regulatory requirements. The noncompliance may be either on the part of the participant, the investigator, or the study staff.

It is the responsibility of the Investigator and study staff to use continuous vigilance to identify and report deviations according to Sponsor procedures and local IRB requirements. All staff involved in the conduct of a clinical trial shall be aware of the specific protocol requirements for completing study visits and notify the PI in the event of any breach of protocol as soon as it is discovered. All deviations from the protocol must be addressed in the participant’s source documentation. As applicable, the study staff will develop and promptly implement corrective actions in response to any deviation.

8.0 Statistical Considerations

The purpose of this trial is to estimate adverse event rates and immune responses to the sporozoite inoculation and malaria infection. This section briefly describes the statistical methods to be used. A separate statistical analysis plan (SAP) will fully describe analysis sets, more technical and detailed descriptions of analysis methods, planned approaches to missing data and outliers, protocol violations and table presentations.

8.1 Sample Size Calculation

A total of 6 subjects will be included in the study and followed for eight weeks. The sample size is based on previous malaria challenge studies conducted using the WRAIR challenge model and represents the minimum number of infectivity controls required for comparison to treatment groups in order to allow determination of whether the trial achieved a successful infectivity rate in addition to providing a sufficient safety profile.

8.2 Analytical Plans

8.2.1 Analysis Population

The summaries and statistical analysis of safety, tolerability and infectivity, primary endpoints, will be provided for the safety population, defined as all subjects receiving the sporozoite challenge. This is the population of primary interest.

The immunology population will include all participants for whom assay results are available for the proposed humoral and cell-mediated immunology assays. Whether a subject is evaluable will be defined in the SAP.

Data for participants who receive prohibited therapy after enrollment may be exluded from analysis populations. Determination of exclusion from analysis will be evaluated during report planning at the end of the study period.

8.2.2 Infectivity Analysis

Detection of peripheral parasitemia by blood smear will be used to determine successful malaria infectivity, another primary endpoint. The number and percentage of participants experiencing successful infectivity will be tabulated, from all subjects who received sporozoite challenge. The time between the primary *P. falciparum* challenge and development of microscopic and sub-patent parasitemia will also be reported.

8.2.3 Safety analysis

AEs and SAEs will be tabulated using MedDRA preferred reference terms. The number and percentage of participants experiencing each specific AE (solicited or unsolicited) or SAE will be tabulated by severity and by relationship to receipt of the sporozoite inoculation. For the calculations in these tables, each participant's AE or SAE will be counted once under the maximum severity or the strongest causal relationship to the study product.

8.2.4 Immunology Analysis

A descriptive, exploratory analysis will be performed using the immunology population described in Section 8.2.1. Qualitative assay data analysis will be performed by tabulating the frequency of positive responses for each assay at each timepoint that an assessment is performed. Crude response rates will be presented with their corresponding exact 95% confidence interval estimates.

For assay variables that have continuous or count-type measurements, graphical and tabular summaries of the underlying distributions will be made. Means or medians with associated 95% confidence intervals will be presented. Box plots of assay values will also be generated. These summaries may be performed on transformed data to better satisfy assumptions of symmetry and homoscedasticity.

9.0 Quality Control and Quality Assurance

Seattle BioMed has several quality control and quality assurance systems in place to assure the safety of study subjects and to ensure the collection of accurate, consistent, complete, and reliable data generated and reported from a clinical trial.

A Trial Steering Committee, composed of the Investigator, Sponsor Medical Monitor, secondary Sponsor Medical Monitor and a representative of the funding sponsor (PATH MVI), is established for the review of the protocol, adverse events, regulatory responsibilities and trial-related issues.

An independent Safety Monitoring Committee is available for review of any safety problems if deemed necessary by the Investigator, Sponsor Medical Monitor or Trial Steering Committee as described in section 6.9.1.

During the clinical trial a Sponsor appointed Study Monitor will conduct on-site visits to ensure that the study is conducted according to the protocol, procedures and regulatory requirements. During the visit the Study Monitor will review each participant's case report forms and supporting source documents, signed informed consent forms, IRB approvals of protocol/amendments, consents, and recruiting documents and regulatory files. The review will be done according to standard operating procedures and a monitoring plan, to assure data accuracy, protocol compliance, and adherence to regulatory requirements.

The Study Monitor will also identify any protocol violations/deviations (as defined in Section 7.6) and confirm that they have been recorded in participant's source documents and reported to the Sponsor and IRB, if applicable.

Prior to the start of the trial, clinical trial staff will be trained during a study initiation visit. The following documents will be reviewed with the staff and trial processes will be explained:

> Investigator's Brochure; Protocol (and Amendments) – including study evaluations and procedures, participant recruitment, screening, enrollment and retention; IRB communications, reporting and approvals; informed consent procedure and form; safety monitoring and adverse event reporting; required documents and records; laboratory and test procedures, including handling of biological specimens; investigational product requirements; completion of case report forms; investigator responsibilities.

The study may be audited by regulatory agencies at any time. If contacted by a regulatory agency for an audit, please call the Sponsor's study monitor immediately. Contact information for the Sponsor's study monitor is included in the Study Binder.

The study may be audited by the Sponsor or its representative at any time to ensure compliance with regulations, policies, procedures and the protocol. Selection of the site for an audit is based on various criteria and is usually pre-arranged with the site. Audit findings will be discussed with the Principal Investigator in an exit interview, summarizing the observations and documented in a formal report. An audit certificate will be issued at the completion of each audit. The audit certificate is a statement that an audit has taken place and is not an indication that the study meets the appropriate requirements.

10.0 Ethics and Responsibility

10.1 Investigator Responsibility

The investigator will conduct the study according to the IRB approved protocol/amendments and government regulations and only make changes after notifying the sponsor/IRB or to eliminate an immediate hazard in order to protect the safety, rights or welfare of subjects. All deviations from/changes in the protocol, unanticipated problems and changes in the investigator will be

documented and reported to the sponsor and IRB, as required by their policies and procedures.

The investigator will personally conduct or supervise the trial. If the investigator delegates responsibilities to clinical trial staff, s/he will ensure that they will be informed about the protocol, the investigational product and their trial-related duties and functions. Training of staff should also include training on the protocol, regulatory requirements and Good Clinical Practices. The training and delegation of responsibilities will be documented on the appropriate form(s). The investigator will maintain adequate and accurate records documenting the conduct of the clinical trial as described in Section 7.0. These records will be available for inspection by sponsor representatives, regulatory authorities and the IRB. As part of the conduct of the trial, the investigator is required to provide periodic reports to the IRB, per its' policies and procedures.

The investigator will obtain informed consent from subjects or subjects legally authorized representative, using an IRB approved consent form. The consent process is described in Section 5.1.2.

The investigator will report all adverse events to the sponsor as described in Section 6.4.

10.2 Compensation

Participants will be compensated for time and travel to participate in this trial. Compensation is pro-rated based upon completion of specific visits and is not contingent upon completing the entire study. A compensation schedule is provided in Appendix E. Compensation for interim visits to follow up clinical or laboratory abnormalities will be provided at the Investigator's discretion, up to $50. If a participant withdraws from the trial, payment will be made based on the last completed visit. Information about compensation, including the amount and schedule of payment(s) and applicable reporting to the IRS, will also be described in the informed consent form.

10.3 Exclusion of Special Populations

No minors or children will be allowed to participate in this clinical trial.

10.4 Institutional Review Board

The Investigator must obtain approval of a clinical protocol, amendments, informed consent forms, recruitment and advertising materials from a duly constituted IRB and comply with its policies, procedures and conditions. The Investigator may not start the trial or make changes to the protocol without prior approval from the IRB, unless it must be done to protect the safety of a

participant. IRB approval to conduct the trial must be obtained prior to the start of the study or prior to implementation of protocol amendments. Annual re-approval of the trial is required, if applicable by study duration. The investigator is also responsible for obtaining approval of any additional documents designated by the IRB.

10.4.1 Protocol Modifications

All amendments/modifications will be reviewed and approved by the Sponsor, and submitted for review by the IRB of record. In the event of a protocol modification, the informed consent must also be revised, if applicable, and submitted for approval to ensure alignment with any amendment to the protocol. Any subject already enrolled in the study will be informed about the revision and asked to sign the revised informed consent. A copy of the revised, signed, and dated informed consent will be given to the subject. All original versions of the informed consent will be retained in the protocol regulatory file and a copy will be retained in the clinic medical record.

10.4.2 Reporting

The Investigator will provide periodic progress reports to the IRB according to its policies and procedures for its continuing review of the research and report adverse events and unanticipated problems involving risk to participants or others. The Investigator will provide a final report to the Sponsor shortly after completion of the trial.

10.5 Participant Confidentiality and Privacy

Seattle BioMed is not a HIPAA covered entity.

Confidentiality must be maintained and private information must be secured for participants in a clinical trial both during and after the participant is no longer participating in the clinical trial. Documentation, data, and all other information generated for a participant will be held in strict confidence whether the information was obtained verbally, recorded on paper, or in electronic files. This confidence extends to cover testing of biological samples in addition to the clinical information. The study database will identify study participants only by a study identification number and will not contain identifying information such as name, address, national identification number (e.g., social security number), medical record number, or personal contact information. No identifying participant information concerning the study or the data will be released to any unauthorized third party without prior written approval of the participant except as necessary for monitoring by the IRB/IEC, the US FDA or the Sponsor or as

required by law or in the case of an emergency, to facilitate medical care. Participants will be made aware of the occasions when information may be released without their consent. Participant records will be stored in locked files. Only authorized individuals will have access to source documents that contain participant identifying information. Confidentiality and private information will also be maintained for participant's biological specimens collected for future studies as described in Section 5.5.5.

10.6 Research Related Injury

If a participant becomes ill or injured as a direct result of study participation and requires additional medical evaluation or referral for treatment, the Sponsor will ensure provision of reasonable and necessary medical evaluation and treatment for the illness or injury at a local University of Washington affiliate hospital at no cost to the participant. Medical care costs for study related illness or injury not covered by other sources (participant primary health insurance, etc.) will be covered by the Sponsor. Payments for medical care by the Sponsor will be coordinated with appropriate payees. Participants will not be otherwise compensated for study related injuries.

11.0 Publication and/or Presentation Policy

Following completion of the study, it is anticipated that the results of the study will be presented to the scientific community via oral presentations and written publications. Any proposed publication or presentation will be governed by Seattle BioMed Publication Policies and any proposed presentation, abstract, or manuscript will be made available for review by the Sponsor, MVI or any involved author(s) prior to submission.

REFERENCES

1. Guinovart C, Navia MM, Tanner M, Alonso PL, et al. Malaria: Burden of disease. - Curr Mol Med.2006 Mar;6(2):137-40..

2. Greenwood BM, Fidock DA, Kyle DE, Kappe SHI, Alonso PL, Collins FH, et al. Malaria: Progress, perils, and prospects for eradication.;118(4):1276.

3. Graves PM, Levine MM, eds. In: Battling malaria: Strengthening the U.S. Military Malaria Vaccine Research - A Program Review,. National Academies Press;2006, p. *ix*

4. Chulay JD, Schneider I, Cosgriff TM, Hoffman SL, et al. Malaria transmitted to humans by mosquitoes infected from cultured plasmodium falciparum. - Am J Trop Med Hyg.1986 Jan;35(1):66-8..

5. Hoffman SL. Experimental challenge of volunteers with malaria. - Ann Intern Med.1997 Aug 1;127(3):233-5..

6. Ballou WR, Arevalo-Herrera M, Carucci D, Richie TL, et al. Update on the clinical development of candidate malaria vaccines. - Am J Trop Med Hyg.2004 Aug;71(2 Suppl):239-47.).

7. Rosenbaum J, Sepkowitz K. Infectious disease experimentation involving human volunteers. Clinical Infectious Diseases. 2002 04/01;34(7):963-71..

8. Epstein J, Rao S, Williams F, Freilich D, Luke T, Sedegah M, et al. Safety and clinical outcome of experimental challenge of human volunteers with plasmodium falciparum–Infected mosquitoes: An update. J Infect Dis. 2007 07/01;196(1):145-54.

9. Manson P. Experimental proof of the mosquito-malaria theory. 1900. - Yale J Biol Med.2002 Mar-Apr;75(2):107-12.

10. Shortt H. Recent advances in our knowledge of the malaria parasite. Br Med J.1950 Sep 9;2(4679):606-8.).

11. Vanderberg JP. Reflections on early malaria vaccine studies, the first successful human malaria vaccination, and beyond. Vaccine. 2009 1/1;27(1):2-9.

12. Collins WE, Jeffery GM. A retrospective examination of sporozoite- and trophozoite-induced infections with plasmodium falciparum: Development of parasitologic and clinical immunity during primary infection. - Am J Trop Med Hyg.1999 Jul;61(1 Suppl):4-19..

13. Clyde DF, Most H, McCarthy VC, Vanderberg JP, et al. Immunization of man against sporozite-induced falciparum malaria. - Am J Med Sci.1973 Sep;266(3):169-77.

14. Rieckmann KH. Human immunization with attenuated sporozoites. - Bull World Health Organ.1990;68 Suppl:13-6.

15. Rieckmann KH, Beaudoin RL, Cassells JS, Sell KW, et al. Use of attenuated sporozoites in the immunization of human volunteers against falciparum malaria. - Bull World Health Organ.1979;57 Suppl 1:261-5..

16. Hoffman S, Goh L , Luke T, Schneider I, Le T, Doolan D, et al. Protection of humans against malaria by immunization with Radiation- Attenuated plasmodium falciparum sporozoites. J Infect Dis. 2002 04/15;185(8):1155-64.

17. Church LW, Le TP, Bryan JP, Gordon DM, et al. Clinical manifestations of plasmodium falciparum malaria experimentally induced by mosquito challenge. - J Infect Dis.1997 Apr;175(4):915-20.

18. Verhage DF, Telgt DS, Bousema JT, Hermsen CC, et al. Clinical outcome of experimental human malaria induced by plasmodium falciparum-infected mosquitoes. - Neth J Med.2005 Feb;63(2):52-8.

19. Ehrhardt S, Mockenhaupt FP, Anemana SD, Otchwemah RN, Wichmann D, Cramer JP, et al. High levels of circulating cardiac proteins indicate cardiac impairment in african children with severe plasmodium falciparum malaria. Microb Infect. 2005 8;7(11-12):1204-10.

20. Bethell DB, Phuong PT, Phuong CX, Nosten F, et al. Electrocardiographic monitoring in severe falciparum malaria. - Trans R Soc Trop Med Hyg.1996 May-Jun;90(3):266-9.).

21. Bregani ER, Tien TV, Cabibbe M, Figini G, et al. Holter monitoring in children with severe plasmodium falciparum malaria during i.v. quinine treatment. - J Trop Pediatr.2004 Feb;50(1):61.

22. Gaziano TA, Young CR, Fitzmaurice G, Atwood S, et al. Laboratory-based versus non-laboratory-based method for assessment of cardiovascular disease risk: The NHANES I follow-up study cohort. - Lancet.2008 Mar 15;371(9616):923-31.

23. U.S. Department of Health and Human Services, Food and Drug Administration. Guidance for clinical investigators, sponsors, and IRBs adverse event reporting to IRBs improving human subject protection. January 2009.

24. Division of Acquired Immunodeficiency Syndrome (DAIDS), NIAID, NIH. Division of AIDS table for grading the severity of adult and pediatric adverse events. 2004 Version 1.0, December 2004.

25. Division of Microbiology and Infectious Diseases (DMID), NIAID, NIH. Division of microbiology and infectious diseases adult toxicity Tables—DRAFT November 21, 2007.

26. U.S. Department of Health and Human Services, Food and Drug Administration Center for Biologics Evaluation and Research. Guidance for industry: Toxicity grading scale for healthy adult and adolescent volunteers enrolled in preventive vaccine clinical trials. September 2007.

27. Nieman A-E, de Mast Q, Roestenberg M, Wiersma J, Pop G, Stalenhoef A, Druilhe P, Sauerwein R, van der Ven A. Cardiac complication after experimental malaria infection: a case report. - Malaria Journal 2009; 8:277

Appendix A: Study Visit Procedures

Visit:	**01**[1]	**02**	**03**	**04-07**	**08-17**	**18**	**19**	**20**	**21**	**22**	**23**	**P1**[2]	**P2**[2]
Day:	-56 to -1	D0	D1	D5-8	D9-18	3D p d/c	D28	D35	D42	D49	D56	3M	6M
Procedure	Screening	Chall.			Hotel Phase	Phone F/U						Post-study f/u	
**Study Procedures**													
Informed Consent	X	—	—	—	—	—	—	—	—	—	—	—	—
Assessment of Understanding	X	—	—	—	—	—	—	—	—	—	—	—	—
Medical History	X	—	—	—	—	—	—	—	—	—	—	X	X
Complete Physical Exam	X	—	—	—	—	—	—	—	—	—	—	—	—
Abbreviated Physical Exam	—	X	X	X	X	—	X	X	X	X	X	—	—
Electrocardiogram (ECG)[3]	X	—	—	—	—	—	—	—	—	—	—	—	—
Pregnancy Prevention Counseling/Compliance[4]	X	X	—	—	X	—	—	—	—	—	—	—	—
Laboratory Assessment (see below)	X	X	X	X	X	—	X	X	—	—	X	X	X
HIV pre/post test Counseling and Assessment	X	—	—	—	—	—	—	—	—	—	—	—	—
Confirm Eligibility	X	—	—	—	—	—	—	—	—	—	—	X	X
Concomitant Medications	X	X	X	X	X	X	X	X	X	X	X	—	—
Intercurrent Illness / Adverse Experience	—	X	X	X	X	X	X	X	X	X	X	X	X
**Laboratory Assessment**													
Urine dipstick	X	—	—	—	—	—	—	—	—	—	—	—	—
Pregnancy testing (urine or serum HCG) [5]	X	X	—	—	—	—	—	—	—	—	—	—	—
CBC with WBC differential	X	—	—	—	—	—	—	—	—	—	—	—	—
Chemistry panel[6]	X	—	—	—	—	—	—	—	—	—	—	—	—
HIV ELISA +/- WB	X	—	—	—	—	—	—	—	—	—	—	—	—
Hepatitis BsAg, anti Hepatitis C Antibody	X	—	—	—	—	—	—	—	—	—	—	—	—
Immunology Labs (per Appendix B)[7]	X	X	X	X	—	—	—	X	—	—	X	X	X
Safety labs[8]	—	—	—	—	X	—	—	X	—	—	X	—	—
RT-PCR[9]	—	—	—	X	X	—	X	—	—	—	—	—	—
**Challenge Procedures**													
Challenge	—	X	—	—	—	—	—	—	—	—	—	—	—
Reactogenicity assessments[10]	—	X	X	X	—	—	—	—	—	—	—	—	—
Peripheral Blood Smear	—	—	—	X	X	—	X	—	—	—	—	—	—

1 Screening may occur over the course of several visits up to and including Day 0, but prior to challenge. See Table 5 for study visit windows.
2 Performed as part of periodic long term follow up observation for immune assessment under separate consent; phone call to all enrolled participants at 6 months post challenge
3 Done at screening and as needed throughout the study for symptomatic evaluation
4 For female participants, will occur Visit 01, 02 and 08
5 For female participants, may be performed during screening but will also be done on the day of challenge D0, prior to the challenge
6 Per Table 1-1 (includes AST/ALT, total bilirubin, alkaline phosphatase and serum Cr).
7 Baseline immunology labs may be drawn during screening or at enrollment prior to challenge
8 Per Table 1-2 (includes CBC, AST/ALT, Cr) performed on day of first positive blood smear, 35, 56 and as needed for AE assessment and follow-up
9 RT-PCR performed twice daily from day 5 until 1st positive blood smear then daily until hotel discharge and at visit 19
10 Recorded daily for 5 days post challenge; participant diary collected at D5 visit

Appendix B: Laboratory Procedures

Volume (ml)																
			Visit:	**1**	**2**	**3**	**4-7**	**8-17**	**18**	**19**	**20**	**21**	**22**	**23**	**P1**[1]	**P2**
	**Assay**		Day:	-56 to -1	D0	D1	D5-8	D9-18	D3 p d/c	D28	D35	D42	D49	D56	3M	6M
**Blood Collection**	**Location**	**Tube**														
**Screening Assays**[2]	CLIA Lab															
CBC with differential		EDTA		3												
Chemistry Panel[3] and HBsAg/anti-HCV		SST		7.5												
HIV EIA/WB		SST		7.5												
**Malaria Infection Assays**	MCTC & UW															
Peripheral Smear & RT-PCR[4]		EDTA					24	60		3						
**Safety Labs**[5]	CLIA Lab															
Complete Blood Count		EDTA						3			3			3		
Chemistry Panel		SST						5			5			5		
**Immunology Labs**	MCTC Core Lab															
Humoral Assays																
ELISA[6]		SST			5	5	5	5			5				5	5
Cellular Assays																
ELISpot[7]		NaHep			20		20	20			20			20	20	20
**Specimen Storage**[8]	Core Repository															
PBMC		NaHep		40		10	50	50			40			40	40	40
Serum		SST		5		5	5	5			5			5	5	5
**Total**				**63**	**25**	**20**	**104**	**148**	**0**	**3**	**78**	**0**	**0**	**73**	**70**	**70**
**56 day cumulative total**				**63**	**88**	**108**	**212**	**360**	**360**	**363**	**441**	**441**	**441**	**451**	**221**	**70**
**Urine collection**																
Urine dipstick, U/A, Umicro				X	—	—	—	—	—	—	—	—	—	—	—	—
Urine pregnancy				X	X	—	—	—	—	—	—	—	—	—	—	—

[1] P1 and P2 visits performed up under separate consent for long-term immunology follow up; otherwise 6 month phone call to all enrolled participants
[2] Screening may occur over multiple visits up to and including D0, prior to challenge
[3] Screening chemistry panel includes AST/ALT, total bilirubin, alkaline phosphatase, and serum creatinine
[4] Twice daily 3ml sample for: AM:BS and RT-PCR, PM: RT-PCR
[5] Safety labs drawn on day of 1st +BS, D35, D56
[6] ELISA done D0, D1, D5, day of 1st + BS, D35
[7] ELISpot done D0, D5, day of 1st +BS, D35 and D56
[8] Specimens for long term storage collected during screening, D1, D5, day of 1st + BS, D35 and D56

Appendix C: Adverse Event Toxicity Grading Scales for Clinical and Laboratory Abnormalities

Table C-1: Toxicity Grading Scale for Local Adverse Reactions

**LOCAL REACTIONS (refers to site of sporozoite inoculation)**				
**ADVERSE EVENT**	**GRADE 1 MILD**	**GRADE 2 MODERATE**	**GRADE 3 SEVERE**	**GRADE 4 POTENTIALLY LIFE-THREATENING**
Pain (pain without touching)<br>Or<br>Tenderness (pain when area is touched)	Mild discomfort; no or minimal interference with usual activities	Discomfort with movement; greater than minimal interference with usual activities; Repeated use of non-narcotic pain reliever >24 hours	Significant discomfort at rest; inability to perform usual activities; Any use of narcotic pain reliever	Pain/tenderness causing inability to perform basic self care; or hospitalization indicated for management of pain/tenderness
Erythema/Redness (largest confluent diameter)*	5.5 – 9 cm	>9 cm but limited to the forearm	Extends to upper arm, axilla or hand.	Necrosis or exfoliative dermatitis
Induration/Swelling*	2.5 – 5 cm; no or minimal interference with usual activities	5.1 – 10 cm or greater than minimal interference with usual activities	> 10 cm or inability to perform usual activities; or ulceration, secondary infection, drainage	Necrosis (involving dermis and deeper tissue)
Pruritis	Itching localized to inoculation site; no or minimal interference with usual activities; improves with topical medications	Itching beyond the injection site but not generalized; greater than minimal interference with usual activities; oral medication required for relief	Generalized itching or inability to perform usual activities; requires repeated, maximal dose oral medication(s) for relief	N/A

* In addition to grading the measured local reaction at the greatest single diameter, the measurement should be recorded as a continuous variable

** Induration/Swelling should be evaluated and graded using the functional scale as well as the actual measurement

Table C-2: Toxicity Grading Scale for Systemic Adverse Reactions

**SYSTEMIC SOLICITED SIGNS/SYMPTOMS**				
**ADVERSE EVENT**	**GRADE 1 MILD**	**GRADE 2 MODERATE**	**GRADE 3 SEVERE**	**GRADE 4 POTENTIALLY LIFE-THREATENING**
Fever (°C)*<br>(°F)*	38.0 – 38.6<br>100.4 – 101.5	38.7 – 39.3<br>101.6 – 102.7	39.4 – 40.5<br>102.9 – 104.9	>40.5<br>>105
Chills/Rigors	No or minimal interference with usual activities	Greater than minimal interference with usual activities	Inability to perform usual activities	NA
Headache	No or minimal interference with usual activities	Greater than minimal interference with usual activities	Inability to perform usual activities; any use of narcotic pain reliever	ER visit or hospitalization
Fatigue/malaise	No or minimal interference with usual activities	Greater than minimal interference with usual activities	Inability to perform usual activities	ER visit or hospitalization
Myalgia (including low back pain)	No or minimal interference with usual activities	Greater than minimal interference with usual activities	Inability to perform usual activities	ER visit or hospitalization
Nausea/vomiting	No or minimal interference with usual activities and/or 1-2 episodes/24 hours	Greater than minimal interference with usual activities and/or >2 episodes/24 hours	Inability to perform usual activities, requires outpatient IV hydration	ER visit or hospitalization for hypotensive shock
Diarrhea	2-3 loose stools or <400 gms/24 hours	4-5 loose stools or 400 – 800 gms/24 hours	6 or more watery stools or >800 gms/24 hours or requires outpatient IV hydration	ER visit or hospitalization
Abdominal pain	No or minimal interference with usual activities	Greater than minimal interference with usual activities	Inability to perform usual activities	ER visit or hospitalization
Arthralgia	Joint pain causing no or minimal interference with usual activities	Joint pain causing greater than minimal interference with usual activities	Joint pain causing inability to perform usual activities	Disabling joint pain causing inability to perform basic self care; ER visit or hospitalization

***** Oral temperature, no recent hot or cold beverages or smoking

Table C-3: Toxicty Grading Scale for Unsolicited Adverse Events

OTHER CLINICAL ILLNESS OR ADVERSE EVENT				
**PARAMETER**	**GRADE 1 MILD**	**GRADE 2 MODERATE**	**GRADE 3 SEVERE**	**GRADE 4 POTENTIALLY LIFE-THREATENING**
Any clinical illness or adverse event not otherwise specified in protocol defined grading table	No or minimal interference with usual activities	Greater than minimal interference with usual activities; may require limited medical intervention	Inability to perform usual activities; requires medical intervention and close follow-up	Inability to perform basic self-care functions OR Medical or operative intervention indicated to prevent permanent impairment, persistent disability, or death

Table C-4: Toxicity Grading Scale for Vital Sign Abnormalities

VITAL SIGN ABNORMALITIES*				
**PARAMETER**	**GRADE 1 MILD**	**GRADE 2 MODERATE**	**GRADE 3 SEVERE**	**GRADE 4 POTENTIALLY LIFE-THREATENING**
Tachycardia – beats per minute	101 -115	116 – 130	>130	ER visit or hospitalization for arrhythmia
Bradycardia – beats per minute**	50 – 54	45 – 49	<45	ER visit or hospitalization for arrhythmia
Hypertension (systolic) mm Hg	141 – 150	151 – 155	>155	ER visit or hospitalization for malignant hypertension
Hypertension (diastolic) mm Hg	91 – 95	96 -100	>100	ER visit or hospitalization for malignant hypertension
Hypotension (systolic) mm Hg	85 – 89	80 – 84	<80	ER visit or hospitalization for hypotensive shock
Respiratory Rate – breaths per minute	17 – 20	21 – 25	>25	Intubation for respiratory distress

* Subject should be at rest for all vital sign measurements.
** When resting heart rate is between 60-100 beats per minute. Use clinical judgment when characterizing bradycardia among healthy subject populations, for example, conditioned athletes.

Table C-5: Toxicity Grading Scale for Laboratory Adverse Events

**LABORATORY ABNORMALITIES**				
**PARAMETER***	**GRADE 1 MILD**	**GRADE 2 MODERATE**	**GRADE 3 SEVERE**	**GRADE 4*** POTENTIALLY LIFE-THREATENING**
Serum Creatinine (mg/dL) – increase by factor	1.1 – 1.3 x ULN**	1.4 – 1.8 x ULN	1.9 – 3.4 x ULN	≥ 3.5 x ULN or requires dialysis
Liver Function Tests: ALT, AST -increase by factor	1.25 – 2.5 x ULN	2.6 – 5.0 x ULN	5.1 – 10.0 x ULN	>10 x ULN
Hemoglobin (Female) gm/dL<br>Normal range:<br>11.7-15.5 gm/dL	10.0 – 11.4	8.5 – 9.9	7.0– 8.4	<7.0
Hemoglobin (Male) gm/dL<br>Normal range:<br>13.2-17.1 gm/dL	11.5 – 12.9	9.5 – 11.4	7.5 – 9.4	<7.5
Hemoglobin change from baseline value gm/dL (male or female)	Any decrease 1.5-2.0 gm/dL	Any decrease 2.1-3.5 gm/dL	Any decrease 3.5 – 5.0 gm/dL	Any decrease >5.0 gm/dL
WBC (elevated) – K/uL<br>Normal range:<br>3.8-10.8 K/uL	10.9 – 15.0	15.1 – 20.0	20.1 – 25.0	>25.0
WBC (decreased) – cell/mm^3	2.0 – 2.5	1.5 – 1.9	1.0 – 1.4	<1.0
Platelets (decreased) - K/uL<br>Normal range:<br>140 – 400 K/uL	100 – 125	50 – 99	25 – 49	<25

* The laboratory values provided are based on Quest Diagnostics normal parameters and critical values.
** "ULN" refers to the institutional the upper limit of the normal range established for the diagnostic laboratory.
*** The clinical signs or symptoms associated with laboratory abnormalities might result in characterization of the laboratory abnormalities as Potentially Life Threatening (Grade 4). For example, a creatinine value that falls within a grade 3 parameter (1.9-3.4 mg/dL) should be recorded as a grade 4 event if the subject required dialysis or hospital admission for renal failure.

Appendix D: Cardiovascular Risk Assessment

As part of the eligibility determination, participants will be screened for cardiac risk based on the NHANES I study criteria (22), and screening electrocardiogram. Results will be documented in the participant source documentation and Cardiac Risk Assessment CRF.

NHANES cardiovascular risk assessment includes the following assessments:

- Evaluation of **risk factors:** Calculated BMI [weight (kg)/height (m^2)], measured Systolic Blood Pressure, smoking status and known diabetes status as reported by the participant on review of medical history
- Evaluation of **5-year cardiovascular risk** using Table D-1 (females) or Table D-2 (males): Low, Moderate, High
- Note: participants under the age of 35 are considered low risk by the NHANES I risk assessment

Only participants classified as low risk by the NHANES I criteria (green and blue categorization below) and a non-significant ECG, as determined by the study cardiologist, are eligible to participate in the study.

Table D-1:

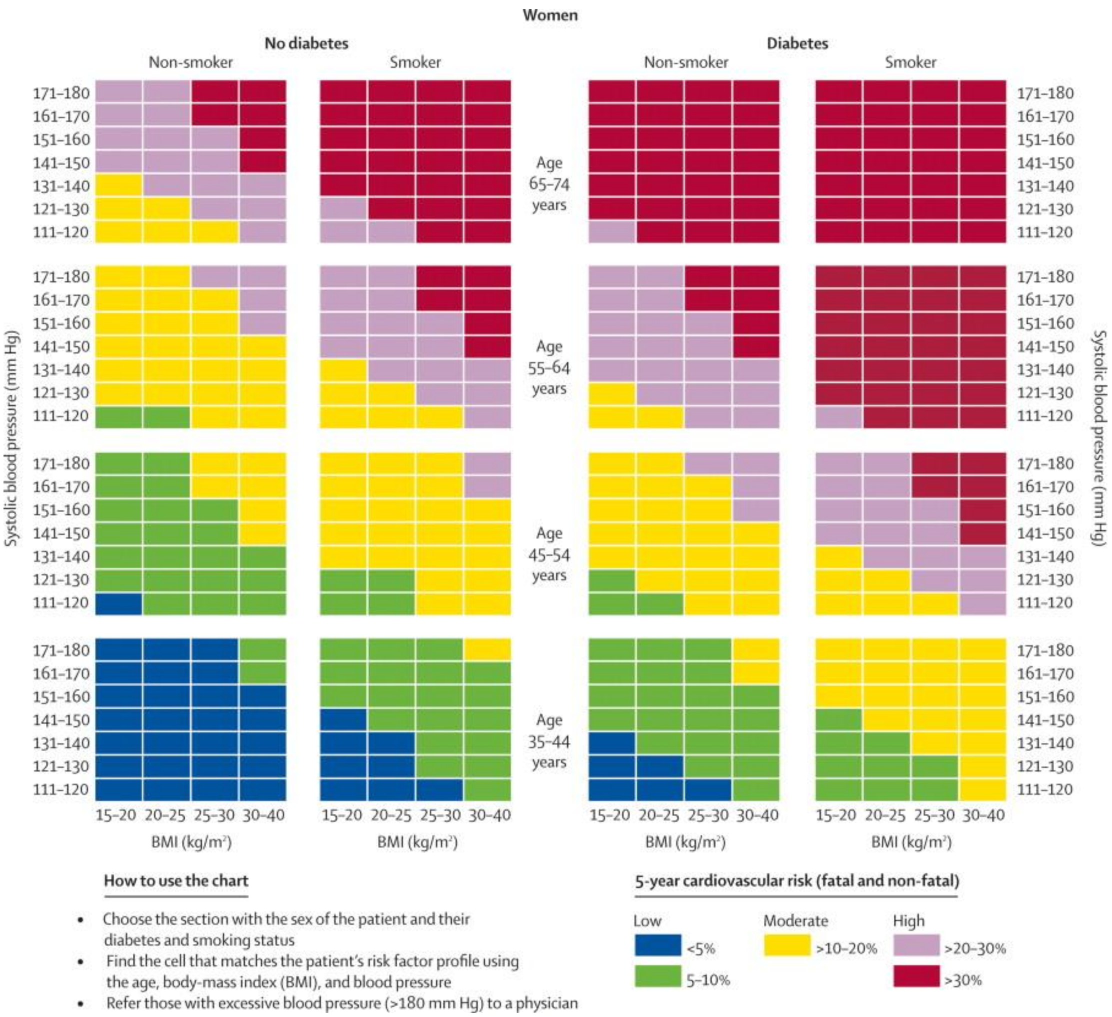
Women
No diabetes
Diabetes
Non-smoker
Smoker
Non-smoker
Smoker
Age 65–74 years
Age 55–64 years
Age 45–54 years
Age 35–44 years
171–180
161–170
151–160
141–150
131–140
121–130
111–120
Systolic blood pressure (mm Hg)
15–20 20–25 25–30 30–40
BMI (kg/m²)
How to use the chart
• Choose the section with the sex of the patient and their diabetes and smoking status
• Find the cell that matches the patient's risk factor profile using the age, body-mass index (BMI), and blood pressure
• Refer those with excessive blood pressure (>180 mm Hg) to a physician
5-year cardiovascular risk (fatal and non-fatal)
Low
<5%
5–10%
Moderate
>10–20%
High
>20–30%
>30%

Table D-2:

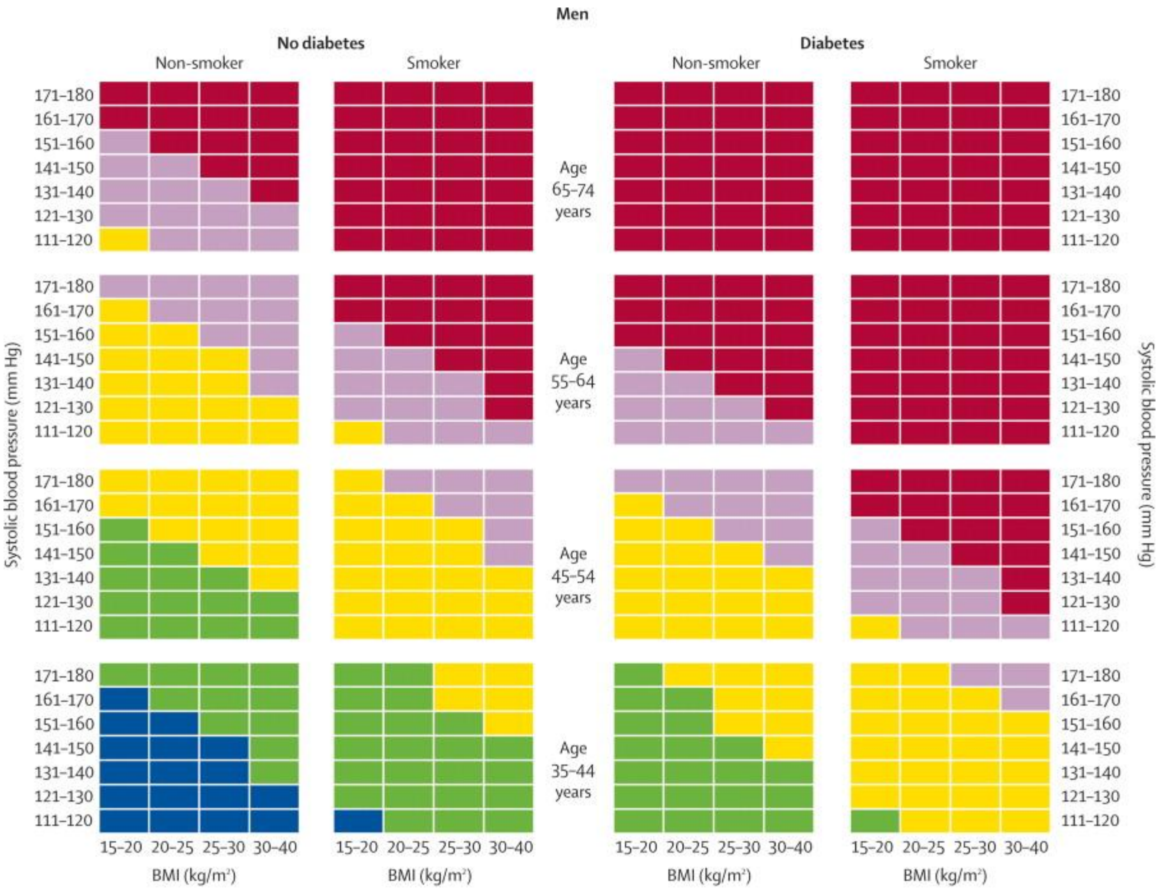

How to use the chart

- Choose the section with the sex of the patient, and their diabetes and smoking status
- Find the cell that matches the patient's risk factor profile using the age, body-mass index (BMI), and blood pressure
- Refer those with excessive blood pressure (>180 mm Hg) to a physician

5-year cardiovascular risk (fatal and non-fatal)

Low: <5%; 5–10%
Moderate: >10–20%
High: >20–30%; >30%

Appendix E: Volunteer Compensation Schedule

Visit #	Study Day	Scheduled Visit	***Approximate*** **Time Allotment**	Visit Activity	Compensation
01	-56 to Day 0	Screening	2 hours	Study explanation, informed consent, medical history/exam, screening blood draw, and ECG	$25
02	Day 0	Challenge	3-4 hours	Challenge day/enrollment, verify eligibility, blood draw, pregnancy testing	$150
03	Day 1	Reactogenicity Assessment	1 hour	Brief physical exam, medication and symptom review, dispense diary and instructions, blood draw	$50
04-07	Day 5-8	Prepatency Clinical Assessment	2 visits/day x 30-40 minutes	AM: Brief physical exam, review of medications and/or symptoms, blood draw, PM: Blood draw	$50/day=$200
08-17	Day 9-18	Hotel Phase	Overnight (up to 10 days)	Overnight stay at hotel, daily physical exam, symptom and medication review, AM/PM blood draw	$100/day up to $1000
18	3 days post hotel discharge	Phone call	15 minutes	Brief follow-up phone call for medication and symptom review	none
19	Day 28	Clinical Assessment Visit	1 hour	Brief physical exam, medication and symptom review, blood draw	$50
20	Day 35	Clinical Assessment Visit	1 hour	Brief physical exam, medication and symptom review, and blood draw	$50
21	Day 42	Clinical Assessment Visit	30 minutes	Brief physical exam, medication and symptom review	$25
22	Day 49	Clinical Assessment Visit	30 minutes	Brief physical exam, medication and symptom review	$25
23	Day 56	Final Visit	1 hour	Final visit: Brief physical exam, medication and symptom review, and blood draw	$100
P1	Month 3	Extension Visit (*optional*)	30 minutes	Blood draw and review of medical history	$50
P1	Month 6	Extension Visit (*optional*)	30 minutes	Blood draw and review of medical history	$50

Total possible compensation: up to $1775 *

* Compensation for interim visits, up to $50, will be provided at the discretion of the Investigator.
